# Influence of the second layer on geometry and spectral properties of doped two-dimensional hexagonal boron nitride

**DOI:** 10.1007/s00894-020-04456-8

**Published:** 2020-07-27

**Authors:** Michał Chojecki, Ewa Lewandowska, Tatiana Korona

**Affiliations:** 1 Faculty of Chemistry, University of Warsaw, ul. Pasteura 1, 02-093 Warsaw, Poland; 2 V LO im. ksiȩcia Józefa Poniatowskiego, ul. Nowolipie 8, 00-150 Warsaw, Poland

**Keywords:** Hexagonal boron nitride, Point defects, Bilayer, TD-DFT

## Abstract

Influence of the additional layer of hexagonal boron nitride (h-BN) on structure, energetics, and electronic spectra of a layer doped with magnesium, silicon, phosphorus, aluminum, or carbon atoms has been examined by theoretical methods. The h-BN layers are modeled as BN clusters of over thirty atoms with the defect in the center. The calculations show that atom positions undergo some modifications in the presence of the second layer, which in several cases lead to significant changes in electronic spectra, like (i) modifications of the character of some states from local excitation to a partial charge transfer; (ii) redshift of the majority of lowest excitations; (iii) absence or appearance of new states in comparison with the monolayers. For instance, a zero-intensity excitation below 4 eV for the carbon atom in place of boron transforms into a dipole-allowed one in the presence of the second layer. A comparison of the interaction energies of doped and undoped clusters shows a strong dependence of the stabilizing of destabilizing effect on the dopant atom, the replaced atom, and in some cases also on the stacking type (AA’ or AB). The stabilization energy per BN pair, calculated for two undoped clusters, is equal to − 31 and − 28 meV for the AA’ and AB stacking, respectively, thus confirming a larger stability of the AA’ stacking for the h-BN case.

**Electronic supplementary material:**

The online version of this article (10.1007/s00894-020-04456-8) contains supplementary material, which is available to authorized users.

## Introduction

The two-dimensional (2D) hexagonal form of boron nitride (h-BN) [1] has gained a lot of attention in the past few years because of its structural similarity to graphene [2]. Monolayers of h-BN can be produced by e.g. the exfoliation method [3]; see also, the review paper of Zhang et al. [4]. Other than in graphene, the h-BN has a large band gap of about 6 eV [5], which may be an attractive feature in various applications. There are many studies of photoexcitation properties of h-BN, which reveal that their spectra heavily depend on the samples’ origin. Such dependence can be explained by the existence of various defects, among which simple substitutional defects, as well as more complex ones (e.g., a combination of a vacancy and a substitution), were proposed in order to explain numerous features detected in the electronic spectra. Among substitutional defects, those involving carbon are the most popular, since the organic carrier for boron introduces carbon into the reaction pot during the molecular epitaxy process [6, 7]. Substitutional defects made of other atoms, e.g., with oxygen, nitrogen, silicon, phosphorus, aluminum, and magnesium, as well as boron and nitrogen vacancies, were examined as well, both experimentally and theoretically [7–21].

Undoped bilayers of h-BN were studied by several research groups; see, e.g., DFT+vdW studies of Rydberg et al. [22] and Marom et al. [23] giving the interlayer binding energy of 26 meV per atom of the monolayer for the former paper and 86 meV for the latter (for the AA’ stacking, see below), and the Diffusion Monte Carlo (DMC) investigation of Hsing et al. [24], where the value of 81 meV per 2BN (i.e., about 40 meV per B−N bond) was reported. Carbon-doped h-BN bilayers were studied by Xie et al. with the DFT method (with no dispersion included) [25]. Much more studies were performed for graphene bi- and multilayers [22, 26, 27], usually with DFT-based approaches, and also with quantum Monte-Carlo or even with symmetry-adapted perturbation theory (SAPT) [28, 29] with the DFT description of monomers [30, 31].

In our recent publication [32], we presented a study of defect-induced changes in geometry and spectral properties of the h-BN monolayer with an emphasis on carbon defects. In that paper, we also studied vacancy and oxygen substitutional defects, as well as close spatial combinations of carbon defects. The most interesting findings of Ref. [32] are the existence of a large variety of low-energy electronic transitions, which critically depend on a number and motifs of carbon atoms, as well as the spatial localization of the lowest electron transitions for combined defects, like C_B_C_N_, in the region of the defect. The localized character of the excitation explains some spectral features seen for some h-BN samples, like a sharp strong peak with phonon replicas in the 4-eV region [10, 17, 33]. Additionally, for spatially close carbon atoms, at least one vibrational frequency corresponds to an oscillation of large amplitudes for both carbon atoms, which is at least 100 cm^− 1^ above the highest collective h-BN frequency.

Although the results for doped h-BN monolayers are encouraging, usually at least several layers are present in real samples, even if monolayers of h-BN are intended to be studied. Therefore, the natural extension of the monolayers’ study is the examination of how two (or more) layers interact with each other and how the presence of another layer modifies the defect-induced features of geometry and the spectrum of a monolayer. The present work is intended to shed some light on this interesting topic by performing a theoretical study of the doped h-BN. Additionally, for one case of the most common impurity of the h-BN, i.e., carbon, a spectrum resulting from two layers with two point defects residing each on one layer will be presented, which has also been studied recently in Ref. [25].

## Computational details

In the theoretical study of layers of 2D structures, each layer is represented as a finite fragment of the BN surface, called hereafter *a cluster*, while a bilayer is represented as a complex of two clusters (the doped and undoped ones). (One should note parenthetically that in the further discussion of the results we will also use the nomenclature *a layer* or *a bilayer* for a cluster or its complex, since the results are thought to be valid for infinite h-BN surfaces, too.) In our previous work [32], we have examined errors introduced by the finite size of the system by performing a series of calculations with clusters of different sizes, obtained by an encircling of a defect by one, two, or three hexagonal BN rings. The edge boron and nitrogen atoms are saturated with hydrogen atoms, which is a known practice in molecular fragmentation methods (see e.g. Ref. [34]) and which has been used with success in other theoretical works on h-BN (see, e.g., Refs. [18, 19]). Based on our previous experience, we selected clusters with a defect encircled with two rings of BN hexagons as representatives of doped h-BN layers. Note that depending on the defect type (X_N_ or X_B_) two clusters were used for this doping procedure: one with the nitrogen, and second with boron atom in the center (B_18_*N*_19_*H*_15_ and B_19_*N*_18_*H*_15_).

For the case of bilayers, several ways of positioning of layers with respect to each other are possible. These are (i) structures, where atoms of the first layer are placed directly under the atoms of the second layer; and (ii) structures, where the second layer is parallelly shifted with respect to the first one, so that only half of the atoms is placed directly under the atoms of the second layer, while the remaining atoms reside under the ring centers. For the first case, we have the AA or AA’ stacking depending on whether the same or different atom types are placed one under another, respectively, while for the second case the same criterion gives us the AB’ and AB stacking (see e.g. Ref. [35]).

The optimization of geometries of mono- and bilayers has been performed on the density-functional theory (DFT) level with a dispersion-corrected B97-D3 functional [36, 37]. This functional has been utilized by us in our previous study of the doped h-BN monolayers [32] and it has been selected based on the reliability for geometry optimization, especially for intermolecular complexes with a large component of nonpolar attraction (dispersion energy), which is accounted for with help of a dispersion correction in the B97-D3 functional. The SVP [38] basis set has been utilized. A larger def2-TZVP [39] basis was used in our previous work for monolayers, but it is computationally too expensive for bilayers. We have verified, however, that the SVP and TZVP basis sets provide very similar results for geometry optimizations for monolayers. Additionally, the influence of the usage of the larger basis set for the geometric parameters of the h-BN complexes (bilayers) has been verified by performing calculations for the AA’ stacked structure (the “sandwich” structure) of two borazine molecules. The resulting distance between layers has increased by 0.04 Å with the TZVP basis, what is an acceptable difference for intermolecular complexes. The analysis of harmonic frequencies at stationary points confirmed that the obtained structures represent indeed minima on the potential energy hypersurface. Additionally, it turns out that only AA’ and AB stacking for bilayers are energetically close enough to be considered in this study, in agreement with Ref. [35]. The optimized Cartesian coordinates of mono- and bilayer models are available in the Supplementary Information. The interaction energies were obtained with the B97+D3, spin-component-scaled (SCS) MP2 [36, 37, 40], and SAPT(DFT) methods for the optimized geometries of complexes. Additionally, the zero-point vibrational energies (ZPVEs) and deformation energies of clusters in the complex were obtained on the B97+D3/SVP level in order to calculate stabilization energies.

For each one from point defects considered in this study, three starting points were considered for the geometry optimization: one for the planar geometry of the first layer, and two for the out-of-plane geometry—where for the first case the defect pointed towards, and in the second case outside another layer. Additionally, the second layer has been placed either according to the AA’, or according to the AB stacking. From these starting points, the lowest minima have been considered for further analysis (one for AA’, one for AB case).

Time-dependent DFT (TD-DFT) with the Coulomb-attenuating method (CAM) CAM-B3LYP functional [41], with a modified ratio of the exact exchange, as proposed in Ref. [42], has been utilized. Modifications from Ref. [42] allow for a more reliable account of long-range charge-transfer (CT) excitations, which is especially important for bilayer cases, even if this improvement in the description of CT often goes in line with a slight deterioration of excitation energies accuracy for valence excitations. The modified CAM-B3LYP will be denoted as CAM-B3LYP-mod in this paper. The so-called jun-cc-pVDZ basis, which is a truncated aug-cc-pVDZ basis of Dunning [43]—with some highest diffuse functions removed [44]—was used in these calculations. Ten states (in some cases up to twenty states) with the same multiplicity as the ground state were requested. A comparison of CAM-B3LYP-mod with more advanced ab initio methods for a stacked dimer of borazine (see Supplementary Information) allows to conclude that the presented excitation energies for the lowest states are systematically blue-shifted by 0.35 to 0.5 eV.

For the case of open-shell systems, the unrestricted version of the TD-DFT was used. In these cases, a spin contamination of the calculated states was examined in a spirit of Ref. [45] and states with a large spin contamination (which we set to more than 20% for 〈*S*^2^〉) were not considered in further discussion. Excited states were also analyzed with the transition-density partitioning method of Plasser and Lischka [46], where a matrix of CT numbers between fragments is obtained in order to establish, which regions of the molecule are active in the excitation. In this particular case, the analysis was performed by dividing the cluster into the defect and the remaining part of the cluster and by mirroring this partition for the undoped layer. It should be noted that since layers are represented by finite-size clusters, one should be aware of some artifacts resulting from the applied model. In particular, clusters of a slightly different stoichiometry are used for the X_N_ and X_B_ defects: B_18_*N*_19_*H*_15_ with the N atom in the center, and B_19_*N*_18_*H*_15_ with the B atom in the center, respectively. Our previous study [32] shows that this distinction becomes unimportant when the defect is encircled with three shells of hexagonal BN rings, but because of a smaller cluster size utilized in the present study, we still see differences in excitation energies up to 0.2 eV for the corresponding excitations of undoped N- or B-centered clusters. Another artifact, like excitations involving hydrogen atoms, is minimized through utilization of the jun-cc-pVDZ basis set.)

Additionally, the interaction energies between pristine and doped layers were calculated both by the supermolecular (B97+D3 and SCS-MP2) and perturbational SAPT(DFT) methods. The partitioning of the SAPT(DFT) interaction energy allows analyzing the importance of various physical components of this energy, like electrostatics, induction, dispersion, and their exchange counterparts. The asymptotically corrected [47] PBE0 functional [48] and the SVP and TZVP [49] basis were utilized in the latter case. Additionally, the stabilization energies were calculated by adding the deformation energies of the monolayers and the difference between the zero-point vibrational energies of bi- and monolayers to the interaction energy. The correlation part of the supermolecular SCS-MP2 interaction energy and the dispersion component of the SAPT(DFT) interaction energy were calculated at Complete-Basis-Set (CBS) limit by utilizing the Helgaker et al. [50–52] formula for the series of def2-SVP and def2-TZVP basis sets, as it was done previously e.g. in Refs. [53–55]. Density fitting has been used for the DFT, TD-DFT, SAPT(DFT), and MP2 calculations with the corresponding auxiliary basis sets [56, 57].

The calculations were performed with Gaussian [58] (geometry optimizations, TD-DFT spectra), Molpro [59] (SAPT(DFT), EOM-CCSD, ADC(2) calculations), and THEODORE [60] (CT analysis).

## Results and discussion

Let us first discuss the results obtained for the undoped case. The first two lines of Table 1 contain the interaction and stabilization energies of the AA’ and AB-type complexes of pristine clusters. The interaction energies obtained by by DFT+D, SCS-MP2, and SAPT(DFT) methods predict higher stability of the AA’ stacking over the AB one. The contributions from the ΔZPVE and deformation energies are small in this case and the calculated stabilization energy also predicts higher stability of the AA’ case with the stabilization energy per BN pair equal to 31.1 meV (AA’) and 27.8 meV (AB) stacking over the AB one.

**Table 1:** Interaction energies (E_int_), ΔZPVE, deformation energies *E*_*def*_, and stabilization energies E_stab_ of the complexes under study, as well as differences between the AB and AA’ stability energies (ΔE_stab_)

		E_int_							
Complex	Stoichiometry	B97-D3	SCS-MP2	SAPT(DFT)[1]	ΔZPVE	*E*_*def*_	E_stab_[2]	ΔE_stab_	E_stab_/pair[3]
Layer(AB)	B_37_*N*_37_*H*_30_	− 134.6	− 105.3	− 108.4	4.7	1.3	− 99.3	11.7	− 27.8
Layer(AA’)	B_37_*N*_37_*H*_30_	− 153.4	− 119.0	− 122.0	5.5	2.5	− 111.0		− 31.1
Al_B_(AB)	AlB_36_*N*_37_*H*_30_	− 220.5	− 201.2	− 191.1	3.2	59.6	− 138.4	11.0	− 38.8
Al_B_(AA’)	AlB_36_*N*_37_*H*_30_	− 240.0	− 217.5	− 205.7	3.4	64.7	− 149.3		− 41.8
Al_N_(AB)	AlB_37_*N*_36_*H*_30_	− 113.5	− 82.8	− 91.7	5.0	4.7	− 73.1	13.7	− 20.5
Al_N_(AA’)	AlB_37_*N*_36_*H*_30_	− 135.9	− 99.2	− 110.2	6.7	5.7	− 86.8		− 24.3
C_B_(AB)	CB_36_*N*_37_*H*_30_	− 134.8	− 105.2		5.1	1.5	− 98.7	12.5	− 27.6
C_B_(AA’)	CB_36_*N*_37_*H*_30_	− 155.0	− 120.2		6.4	2.6	− 111.2		− 31.1
C_N_(AB)	CB_37_*N*_36_*H*_30_	− 135.2	− 105.5		4.1	1.4	− 100.0	11.9	− 28.0
C_N_(AA’)	CB_37_*N*_36_*H*_30_	− 155.2	− 119.8		5.6	2.2	− 111.9		− 31.3
C_B_–C_N_(AB)	C_2_*B*_36_*N*_36_*H*_30_	− 328.2	− 353.9		13.2	34.8	− 305.9	5.9	− 85.7
C_B_–C_N_(AA’)	C_2_*B*_36_*N*_36_*H*_30_	− 340.7	− 370.1		13.0	45.3	− 311.9		− 87.4
C_B_C_N_(AB)	C_2_*B*_41_*N*_41_*H*_32_	− 151.1		− 122.9	4.5	1.7	− 116.7	17.3	− 28.8
C_B_C_N_(AA’)	C_2_*B*_41_*N*_41_*H*_32_	− 176.8		− 142.1	5.3	2.8	− 134.0		− 33.1
P_B_(AB)	PB_36_*N*_37_*H*_30_	− 131.3	− 102.2	− 105.4	5.3	5.1	− 91.9	13.7	− 25.7
P_B_(AA’)	PB_36_*N*_37_*H*_30_	− 150.6	− 115.9	− 120.2	6.3	3.9	− 105.6		− 29.6
P_N_(AB)	PB_37_*N*_36_*H*_30_	− 124.2	− 94.4	− 100.4	4.8	4.2	− 85.4	14.1	− 23.9
P_N_(AA’)	PB_37_*N*_36_*H*_30_	− 144.9	− 110.1	− 116.9	6.4	4.2	− 99.5		− 27.9
Si_B_(AB)	SiB_36_*N*_37_*H*_30_	− 130.9	− 100.8		4.9	6.8	− 89.1	13.9	− 24.9
Si_B_(AA’)	SiB_36_*N*_37_*H*_30_	− 149.9	− 114.1		6.3	4.8	− 103.0		− 28.9
Si_N_(AB)	SiB_37_*N*_36_*H*_30_	− 119.1	− 88.2		5.1	4.8	− 78.3	14.2	− 21.9
Si_N_(AA’)	SiB_37_*N*_36_*H*_30_	− 140.7	− 104.5		6.7	5.3	− 92.5		− 25.9
Mg_B_(AB)	MgB_36_*N*_37_*H*_30_	− 205.4	− 167.0		4.4	43.2	− 119.3	6.4	− 33.4
Mg_B_(AA’)	MgB_36_*N*_37_*H*_30_	− 224.4	− 179.0		4.4	48.9	− 125.7		− 35.2
V_B_(AB)	B_36_*N*_37_*H*_30_	− 128.9	− 106.8		5.1	1.3	− 100.4	13.1	− 28.1
V_B_(AA’)	B_36_*N*_37_*H*_30_	− 148.4	− 121.2		5.4	2.4	− 113.5		− 31.8
V_N_(AB)	B_37_*N*_36_*H*_30_	− 126.6	− 97.8		3.8	21.9	− 72.1	12.1	− 20.2
V_N_(AA’)	B_37_*N*_36_*H*_30_	− 146.2	− 111.7		4.7	22.8	− 84.2		− 23.6

^1^ SAPT(DFT) interaction energies were calculated for the interaction of two closed-shell monomers only

^2^ The stabilization energy is a sum of the SCS-MP2 interaction energy, ΔZPVE, and deformation energies from the B97-D3 calculations for all complexes but the largest ones (C_B_C_N_(AA’), C_B_C_N_(AB)), for which the the SAPT(DFT) interaction energy has been used

^3^ E_stab_ per one BN pair is expressed in meV, all other quantities are expressed in kJ/mol

The geometry-optimized structures of both types of clusters (boron- and nitrogen-centered) have a bond length of 1.457 Å for a bond with the central atom, in a good agreement with Lebedev et al. [35], who reported 1.455 Å as the bond distance within the layer. The change in the bond length within the layer resulting from an addition of the second cluster turns out to be negligible (not larger than 0.001 Å). The distances between the layers are larger for the AA’ stacking, e.g., a distance between central atoms of the clusters composing the complex is equal to 3.353 Å and becomes 0.01 and 0.02 Å smaller when moving one or two bonds away from the center, again in good agreement with Ref. [35], where the experimental value of 3.33 Å [8] was utilized. The distance between central atoms for the AB stacking is equal to 3.282 Å; i.e., it is about 0.007 Å shorter than that for the AA’ case. (One should note parenthetically that differences in B−N lengths for atoms lying one over another do not exceed 0.015 Å, and on average they are below 0.01 Å. Of course, for the periodic case, these distances should be all the same.) These distances are only slightly shorter than the sum (3.47 Å) of van der Waals radii of boron (1.92 Å) and nitrogen (1.55 Å), which confirms that the layers should be bound noncovalently in this case.

### Geometry of doped mono- and bilayers of h-BN

In this study, we consider single-atom substitutional defects with C, Si, Al, P, and Mg atoms, which gives rise to ten defect types: C_B_, C_N_, Si_B_, Si_N_, Al_B_, Al_N_, P_B_, P_N_, Mg_B_, and Mg_N_ (where *X*_*Y*_ denotes the X atom replacing the Y atom). Additionally, we examine single-atom vacancies denoted as V_B_ and V_N_ for boron and nitrogen vacancy, respectively. For all these defects, we consider the monolayer, i.e., one h-BN cluster with a defect in the center, and the bilayer case, where we include the second (pristine) layer in the AA’ or AB position with respect to the first layer, taking the N- or B- centered undoped cluster for the X_B_ or X_N_ defect types, respectively. As noted above, the AA’ stacking is the most favorable energetically and a small number of defects should not change this situation. The AB stacking is considered for the situation when the dopant atom (or vacancy) resides directly above the atom of the pristine layer. It should be noted in passing that there is another possible configuration for the AB stacking, with the dopant atom (or vacancy) residing above the center of the BN ring of the second layer; however, we have chosen to focus on the former structure only, since a close distance of the dopant atom and the atom from the second layer has a larger potential to trigger changes in geometry and spectral features of the layers. Because of the popularity of carbon as a h-BN dopant, we also include two special cases for this element: (i) two adjacent carbon atoms replacing boron and nitrogen in one layer (C˙BC˙N) and (ii) one C_B_ defect in the upper layer and one C_N_ defect in the lower layer denoted as C_B_–C_N_ with both carbon atoms one over another. The monolayer C˙BC˙N defect has been studied recently by us [32] as a possible candidate for the explanation of the low-energy photoluminescence peak close to 4 eV, while the latter has been considered in Ref. [25]. Note that in order to conform to our modeling strategy of enveloping the defect by two layers of BN rings, clusters in the C˙BC˙N case have a different shape than for one-atom defect (the cluster mimicking a monolayer consists of 42 non-hydrogen atoms instead of 37).

Since from this moment, we will be describing various quantities calculated with either AA’, or AB stacking, we will adhere to the notation where these quantities are followed by the stacking symbol AA’ or AB in parentheses.

The examination of the optimized geometries for monolayers shows that the h-BN network remain planar only for carbon and V_B_ defects, while for all other cases various deviations from planarity occur. Two types of nonplanar structures have been found: either a folded surface, or a cone shape with the dopant atom at the top, with the latter type being the most popular and with the folding occurring for the Mg_N_ and V_N_ defects only.

**Fig. 1:**
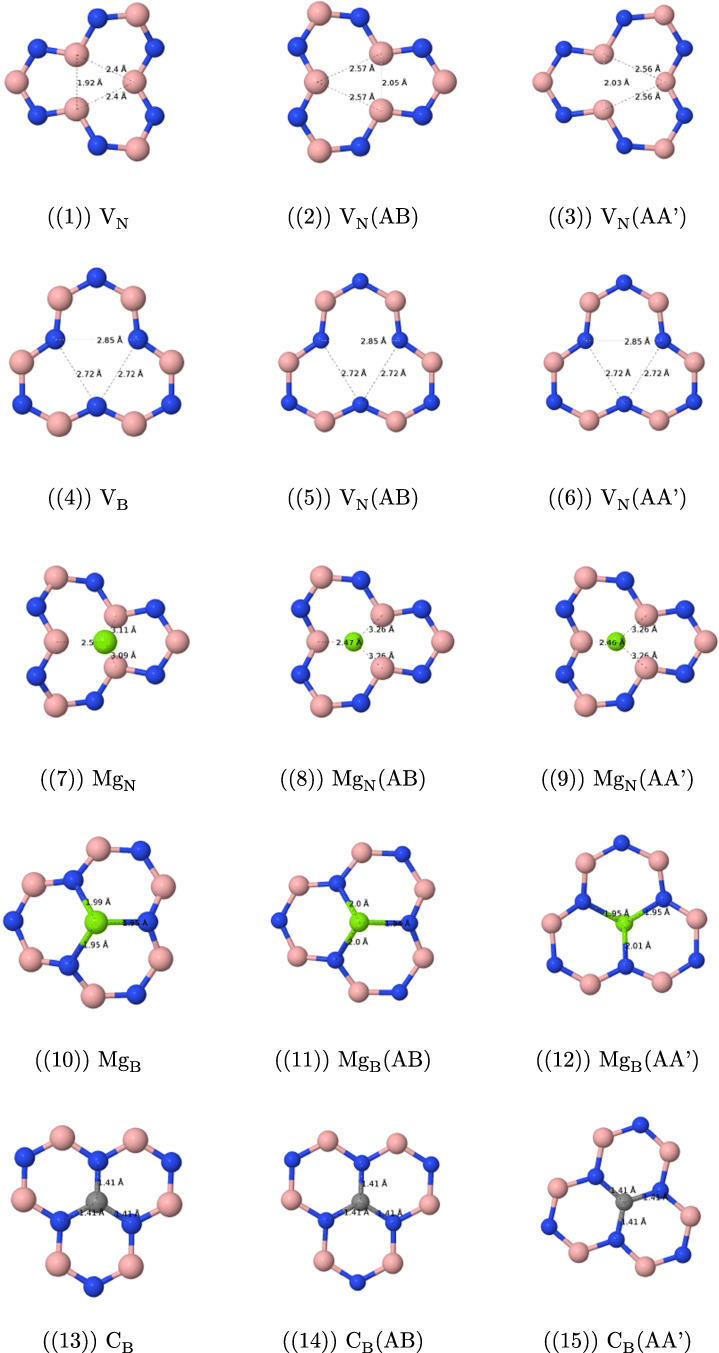

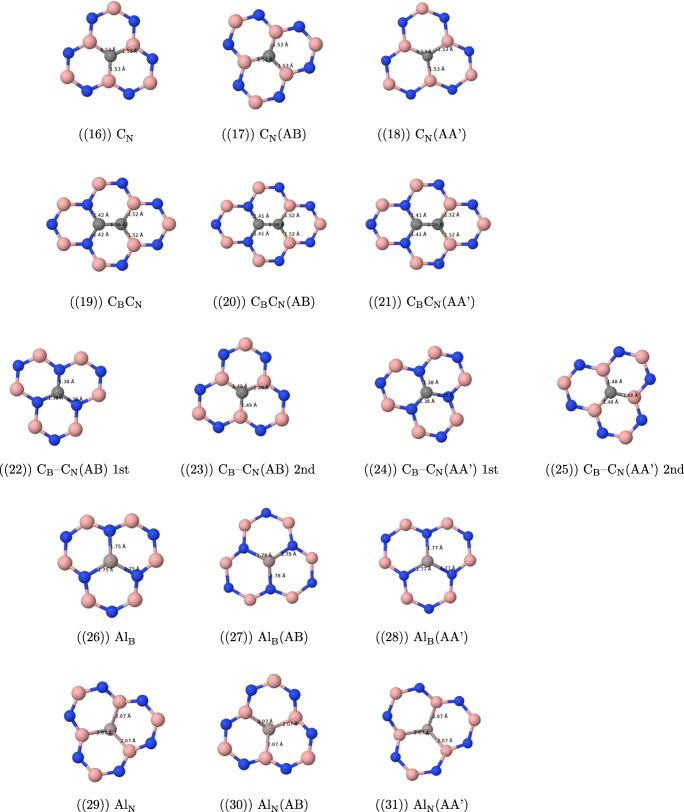

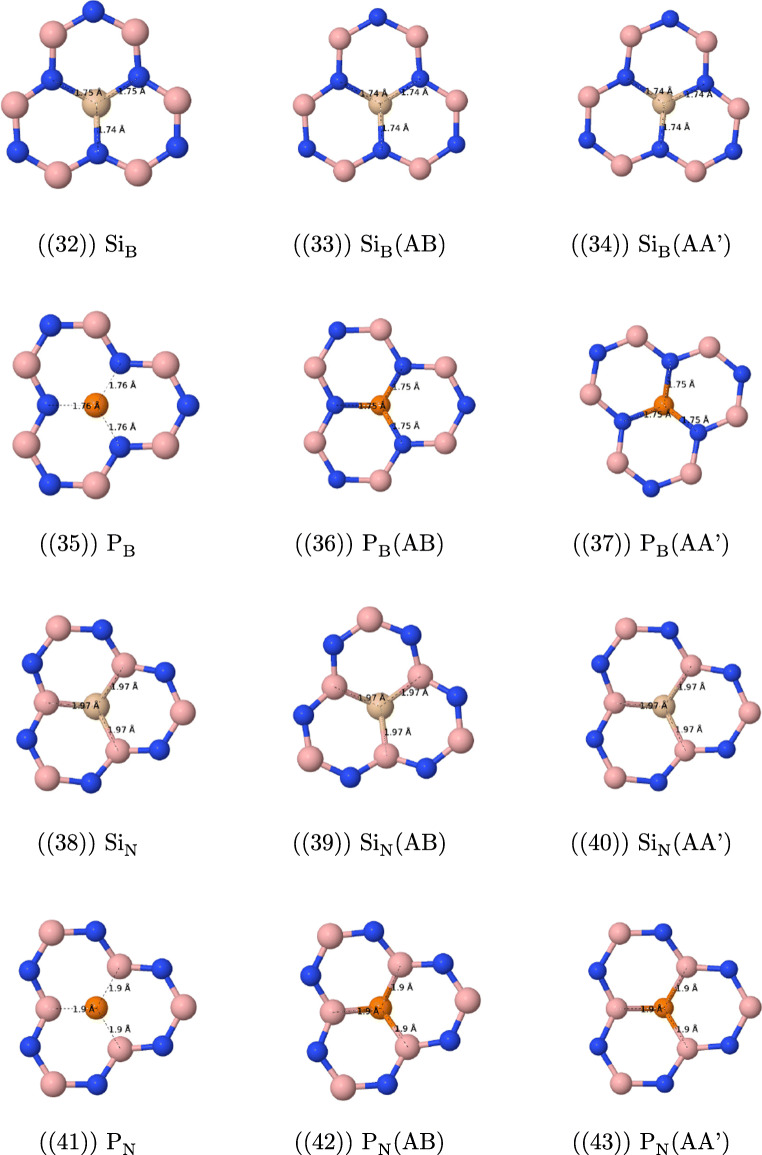
Optimized geometries (solely the core part of the layer containing the point defect) of the structures under study with selected bond lengths

The most important features of the geometries of the studied defects are presented in Fig. 1.

#### Geometric features of vacancy defects

The removal of the nitrogen atom (the V_N_ defect, see Fig. 1(1)–(3)) results in a folding of the cluster and lowers its symmetry around the missing nitrogen atom—instead of three equidistant boron atoms as in the undoped case (with the B−B distance of 2.52 Å), three boron atoms around the hole form an isosceles triangle with a base of length of 1.92 Å and the remaining sides of length 2.41 Å. The former distance is only 0.2 Å longer than a typical single B−B bond (1.72 Å). The addition of the second layer leads to a visible reduction of folding. Additionally, the sides’ lengths of the central boron triangle become 2.03 Å (AA’) and 2.05 Å (AB) for the triangle base and are almost back to the original lengths for two remaining triangle sides, 2.56 Å (AA’) and 2.57 Å (AB). For the case of the AA’ stacking, one from three boron atoms around the hole points toward the second cluster, while for the AB case in the opposite direction.

The removal of the B atom (the V_B_ case, see Fig. 1(4)–(6)) leads again to the symmetry lowering and the equilateral NNN triangle around the hole transforms into the isosceles one, but—contrary to the V_N_ case—the planarity of the cluster is preserved. Distances between three central nitrogen atoms change from 2.52 Å for the undoped case to 2.85 Å (base) and 2.72 Å (two remaining sides of the three-nitrogen triangle). The elongation of these distances can be easily explained by the increased repulsion between three electronegative atoms, which is not counterbalanced anymore by an electropositive boron. The addition of the second layer does not change the geometry of the V_B_ defect neither in the AA’, nor in the AB stacking.

#### Geometric features of magnesium defects

The replacement of the nitrogen atom through magnesium (Mg_N_, see Fig. 1(7)-(9)) turns out to be unsuccessful in a sense that the optimized position of the Mg atom is outside the cluster, so that the closest distance between Mg and B is equal to 2.50 Å for the monolayer and 2.46 (AA’) and 2.47 (AB) Å for the bilayer, while the two remaining boron atoms lie at distances larger than 3.1 Å (3.2 Å for bilayer) from the “dopant” atom. This distance is about 0.2 Å larger than the reported experimental bond length of Mg−B, equal to 2.32 Å [61], and is the same as the reported distance in the crystal structure of the MgB_2_ superconducting material [62]. MgB_2_ is composed of boron honeycomb layers with magnesium atoms placed between the boron layers [63], and it appears that three neighboring boron atoms in the present case “initiate” the removal of the magnesium atom from the layer and its placement as a begin of the second Mg layer. The cluster is folded in a way similar to the V_B_ defect, and actually the present case can be described as the V_B_ defect with an Mg atom placed on top of the boron isosceles triangle. The second h-BN layer significantly reduces the folding, similarly to the V_N_ case.

The Mg_B_ defect (see Fig. 1(10)–(12)) has a cone shape with the Mg−N distances equal to 1.95 (two bonds) and 1.99 Å (one bond) for the monolayer. These lengths are even smaller than reported Mg−N lengths (2.08–2.10 Å) from the crystallographical data [61]; therefore, a covalent binding is expected between Mg and all three nitrogen atoms. The Mg-decorated cone points towards the second layer for both AA’ and AB stackings with the closest interlayer Mg−N distance equal to 2.25 (AA’) and 2.26 Å (AB). Such a small distance indicates that a a sort of weak covalent bond *between* both layers should be present, since the sum of van der Waals radii of Mg (1.73 Å) and N is equal to 3.28 Å, which is about 1 Å larger than the calculated Mg−N distance. The influence of a second layer on the *intra* layer Mg− distances is small for the AA’ case (two bonds of 1.95 and one of 2.01 Å), while changes for the AB stacking are larger, since in this case the two equal bonds are longer than the remaining bond (there are two bonds of 2.00 and one of 1.94 Å). This finding turns out to be important in the further analysis of the electronic spectra. The Mg_B_ defect weakens the structure of the layer closest to the defect: distances between nitrogen atoms forming a cone base are longer by about 0.5 Å compared with the undoped case. The addition of the second layer partially improves the situation—the N−N distances of the cone base become shorter by 0.1–0.2 Å in comparison with the monolayer. Therefore, the geometry analysis indicates that the addition of the second layer stabilizes the doped layer. Because of the surface deformation caused by the Mg_B_ defect, the interlayer distances between B and N become longer in the vicinity of the defect—the B−N noncovalent bondings next to the defect are about 0.2–0.25 Å longer than for the undoped bilayer.

#### Geometric features of carbon defects

The C_B_ and C_N_ defects (see Fig. 1(1)–(18)) do not destroy the planarity and the *C*_3_ axial symmetry of the h-BN surface and the geometry modifications resulting from an addition of the second layer are very small (at most a couple of thousandths of Å). For the case of the C_B_ defect, the C−N bond length is equal to 1.41 Å for the monolayer, while the distance between the carbon atom and the nearest nitrogen atom of the second layer is equal to 3.29 (AA’) and 3.25 (AB) Å, which is only a couple of hundredths of Å less than the average interlayer distance between nitrogen and boron atoms for the undoped complex. For the C_N_ defect, the intralayer C−B bond lengths are equal to 1.52 Å and distances between the carbon atom and the nearest boron atom of the second layer amount to 3.31 Å (AA’) and 3.26 Å (AB).

As mentioned in “Introduction,” because of the popularity of carbon defects, we decided to enhance our study by adding two selected cases of two neighboring carbon atoms. The first situation occurs when both atoms replace neighboring boron and nitrogen in the same layer (C˙BC˙N, see Fig. 1(19)–(21)). The C−C distance is equal to 1.38 Å, which is a typical value for a carbon bond in aromatic systems. The lengths of the C−N and C−B bonds, equal to 1.42 and 1.52 Å, respectively, are very similar to those of isolated C_B_ and C_N_ defects, and they do not change when the second layer is present. The length of the C−B bond is typical for aromatic systems like 1,2-dihydro-1,2-, 1,3-dihydro-1,3-, and 1,4-dihydro-1,4-azaborines (see e.g. Electronic Supplementary Information of Ref. [64]) where a value of 1.51 Å was found from the MP2 optimization. However, the present value of the C−N bond is about 0.05 Å longer than the value of 1.36 Å found in azaborines, which indicates the single-bonded character of the C−N bond in the present case.

Another interesting defect is represented by two carbon atoms one over another, denoted in the following as C_B_–C_N_ (see Fig. 1(22)–(25)). Both AA’ and AB stackings give rise to a composite defect with almost flat layers: the distance between carbon atoms is equal to 3.12 (AA’) and 2.94 Å (AB), so it is only about 0.2 Å shorter than the average distance between nearest B−N interlayer pairs, so again the deviations from the planar structure are small. The defect is characterized by shorter C−B and C−N bond lengths than for the isolated C_N_ and C_B_ defects: for the C−B bonds, by about 0.03 Å, and for C−N bonds, by about 0.04 Å, so that the latter bond length is similar to the azaborine value, while the former one becomes even shorter than for these prototypical aromatic BN-containing benzene analogs (the AB values are about 0.005 Å longer than the AA’ ones). Interestingly, the geometry optimization detects in the C_B_–C_N_ another minimum, which lies about 13 (AA’) and 7 (AB) kJ/mol higher in energy for the cluster dimer, and for which an extra long C−C bond of 1.68 Å (AA’) and 1.69 Å (AB) has been created. The existence of such a bond leads necessarily to a significant distortion of the C−B bonds, which are elongated up to 1.59 Å (AA’) and 1.58 Å (AB), and of the C−N bonds (1.49 Å and 1.49 Å for AA’ and AB, respectively). The latter minimum was found by Xie et al. [25] as the only one in this publication. A possible explanation of the discrepancy between the results of Xie et al. and our optimization is the lack of dispersion in the PBE functional utilized in Ref. [25]. In fact, we have checked that the minima order change if the PBE or *ω* B97 functionals (both are functionals without a long-range dispersion correction) are utilized. Therefore, the utilization of functionals with a correct description of long-range dispersion is of a primary importance for stacked *π* structures.

#### Geometric features of selected III row atomic defects

Aluminum, silicon, and phosphorus atoms belong to the third row of the periodic table and have therefore larger numbers of electrons than boron or nitrogen, which they are going to replace. Therefore, it can be expected that they do not “fit” into the cavity left after the removal of B or N and will form a cone with a dopant atom at the top. When the second layer is added, the dopant atom moves outside the plane for all cases but Al_B_, where it points towards the nitrogen atom of the second layer.

Let us start a detailed analysis of the geometries from the latter case (see Fig. 1(26)–(28)). The interlayer Al−N distance is equal to 2.16 Å (AA’) and 2.17 Å (AB), which is larger than the experimental value for the Al−N bond, equal to 1.786 Å (https://cccbdb.nist.gov/), but which is nonetheless much smaller than the sum of van der Waals radii of Al and N equal to 3.39 Å (taking 1.84 Å as the radius of Al). Therefore, one can expect the presence of at least partial covalent bonding for this case. Such bondings occur for all three nitrogen atoms in the doped layer (all three Al−N bonds are 1.75 Å long for the monolayer and become about 0.025 Å longer for bilayers). Note that no *C*_3_ symmetry distortion occurs in the Al_B_ case. The distances between three nitrogen atoms around the dopant Al, which can serve as a measure of the deformation of the h-BN surface, are equal to 2.99 Å for monolayer and become 0.09–0.08 Å shorter for bilayers, which indicates that the second layer has a stabilizing effect on the doped layer. Additionally, the distances between the three nitrogen atoms surrounding Al and the closest boron atoms of the second layer are significantly smaller for the AA’ stacking (2.99 Å) than for the undoped AA’ case, so—since no covalent bonding can be created between these atoms—signify that most probably those three N⋯B pairs enter the valence repulsion region and destabilize the AA’ structure in comparison with the AB one.

For the Al_N_ defect (see Fig. 1(29)–(31)), the introduction of the second layer has a weaker effect on geometry parameters around Al: the Al−B distance (equal to 2.07 Å for the monolayer) inside the layer changes for a few thousandths of Å and the side of the boron triangle changes by about 0.015 Å only. As noted above the Al cone points outside the plane, and the distance between Al and the closest B atom of the second layer is as large as 5.3 Å.

The distortion of the h-BN monolayer for the Si_B_ and P_B_ defects is smaller than for the Al_B_ case, e.g., the N−N distance is in the range of 2.8–2.7 Å, which should be compared with the value of 2.5 Å for the undoped cluster (see Fig. 1(32)–(37)). Geometry modifications introduced by the second layer for the silicon and phosphorus defects are quite small, too. For Si_B_ (P_B_), the Si−N (P−N) bond length is equal to 1.75 Å (1.76 Å) for the single cluster and becomes shorter by only several thousandths of Å, when the second layer is present. Similarly, the length of the side of the nitrogen triangle changes by about 0.01 Å only. The closest distance between the dopant atom and the nitrogen atom of the second layer is equal to 4.3–4.4 Å, which precludes any bond formation.

Similarly to the Si_B_ and P_B_ cases, the Si_N_ and P_N_ defects are not sensitive to the addition of the second layer, as far as the geometry is concerned (see Fig. 1(38)–(43)). The Si−B and P−B bonds, which for a single layer have the length of 1.97 and 1.90 Å, respectively, become slightly shorter (by less than 0.005 Å).

#### IR spectra

Before we discuss changes in the IR spectra introduced by the interaction with the second layer, we will consider characteristic features of the pristine mono- and bilayers as simulated by the clusters. It turns out that the characteristic stretching frequencies of the BN network appear at about 1350–1500 cm^− 1^, with the highest frequency rising by about 10 cm^− 1^ for a bilayer. The appearance of higher frequencies for a cluster dimer is in line with the existence of a frequency of about 1600 cm^− 1^ for bulk h-BN [65]. We have investigated this issue a little further for the AA’ stacking type and have found that for three and four clusters one over another, a further increase of the highest lattice frequency is observed, so that the series for *n* clusters one has the frequencies 1498, 1508, 1517, and 1525 cm^− 1^, for *n*= 1, 2, 3, 4, respectively. In addition to in-plane oscillations, one can also find out-of-plane modes, from which one has a nonzero intensity. The frequency of this mode decreases with the number of clusters in the following way: 783, 770, 766, and 760 cm^− 1^, which is also consistent with the experimental findings for bulk h-BN.

Local modifications of the geometry of the layer introduced by defects under study, which in some cases can be quite substantial, allow the assumption that similarly large changes should be observed in the IR spectra of the clusters and their complexes with an undoped cluster. In the previous paper [32], we explored this subject for selected carbon defects for a monolayer case and found out that even if for the single-atom defect one can identify the vibration mode with a large contribution of the dopant atoms, such a vibration has usually a small intensity and cannot be used for the detection of the defect. These observations have been reproduced in the present paper for the C_B_ and C_N_ defects and confirmed for other one-atom defects. The situation looks differently for the case of two neighboring carbon atoms in the same h-BN plane. For this case, a characteristic C−C in-plane stretch vibration of a high intensity appears in the spectrum more than 100 cm^− 1^ above the lattice-stretch frequencies of h-BN. This feature is reproduced in our current study and the calculated frequency is virtually identical for mono- and bilayer cases. The lack of sensitivity of this frequency to the presence of the second layer can be explained by the in-plane character of this oscillation. Its frequency value (equal to 1589 cm^− 1^) is 21 cm^− 1^ higher than the value reported in Ref. [32], which is caused by different basis sets utilized in both studies. There is also an out-of-plane vibration dominated by carbon motions, of energy 811 cm^− 1^ for a single cluster and 807 cm^− 1^ for a dimer (independently on the stacking), which has however a much lower intensity than the C−C stretching. This out-of-plane C−C motion lies about 25 cm^− 1^ above the high-intensity out-of-plane mode of the lattice in the bilayer case. Apart from the localized C−C stretching mode, there is a set of lattice stretching vibrations with the highest one at about 1490 cm^− 1^ for a single cluster and at about 1510 cm^− 1^ for a cluster dimer, thus recovering the behavior of the undoped dimer.

The full set of the simulated IR spectra can be found in the Electronic Supplementary Information.

### Energetic stabilization of bilayers

The interaction energies for AA’ and AB-stacked complexes, computed with various methods (B97+D3, SCS-MP2, and SAPT(DFT)), are presented in Table 1 together with the ZPVE differences (ΔZPVE) and deformation energies *E*_*def*_, and stabilization energies calculated as a sum of SCS-MP2 interaction energies and ΔZPVE and *E*_*def*_ obtained on the B97+D3/SVP level. The complexes can be distinguished by their description in the first column of this table, where the defect type is followed by the stacking type given in parentheses. The SAPT(DFT) energies are available for closed-shell monomer cases only, since although the open-shell SAPT method does exist [66], its application to such large molecules is problematic. Additionally, the stabilization energy per atomic pair is presented (for the V_B_ and V_N_ cases the number of pairs is taken from the parent, i.e., unmodified, molecule), as well as the difference between the stabilization energy calculated with the AB and AA’ stacking. The analysis of the table allows to make a conclusion that the SCS-MP2 and SAPT(DFT) interaction energies agree quite well with each other, with the SCS-MP2 energies systematically higher than SAPT(DFT). A sole exception from this correlation (the Al_B_ defect) has the Al atom close enough to the second layer to create a partially covalent bonding, so the relative accuracy of the perturbation theory is expected to be worse than for the interaction of two closed-shell molecules. In any case, the relative difference between these two approaches is usually well below 10% with only two exceptions (another Al-containing defect). It should be noted that such an agreement between two different methods supports the observations made for other large molecular complexes [67, 68]. Therefore, we utilize the SCS-MP2 interaction energy to calculate the total stabilization energy of the complexes under study. Note that the MP2 interaction energy often strongly overbinds systems with stacked *π* molecules [69]. It should be noted, however, that the perturbation-type models often experience convergence problems if small energy denominators, present in the formulas starting from the second order, appear, which can happen more often in the open-shell cases. Therefore, the interaction energies obtained for the open-shell clusters can be somewhat less accurate than for the closed-shell case.

The perusal of Table 1 allows to make a conclusion that the AA’ stacking is always more stable than the AB one, independently of the point-defect type. The difference in stabilization energies (Δ*E*_stab_) for the AB- and AA’-stacked complexes for the undoped case amounts to 11.7 kJ/mol, while for the doped cases these differences in E_stab_ range from 6 up to 14 kJ/mol. Therefore, some defects make ΔE_stab_ smaller (the Al_B_, Mg_B_, and C_B_–C_N_ defects), while other defects enhance ΔE_stab_ (the remaining defects from this table). This behavior of the ΔE_stab_ is in line with geometrical features of doped bilayers, since three cases, which make the stability gap smaller, have peculiar geometry characteristics with respect to the remaining defects, as discussed in the previous section.

Finally, the stabilization energy per atom of the cluster can be analyzed. This energy corresponds to the stability measure of bilayers with respect to monolayers, which is often presented in the literature. The absolute value of the SCS-MP2 stabilization energy per one BN pair for the undoped AA’ and AB cases is equal to 31.1 and 27.8 meV (we switch to the meV units, since it is usually utilized in the literature). These values are smaller than the results of Rydberg et al. [22] for the AA’ stacking by 20 meV. However, the SCS-MP2 values presented by us are confirmed by the results of the SAPT(DFT) calculations, also listed in Table 1—the SAPT(DFT) and SCS-MP2 interaction energies differ only 0.9 meV per one interacting BN pair. It should be also noted that the extrapolation to the infinite cluster is expected to increase the absolute values of the E_stab_/pair.

A comparison of the stabilization energies per BN pair for doped complexes is less instructive because one from these pairs is different from others, but nonetheless one can note that at least one interesting result: the absolute value of this energy is significantly higher for the C_B_–C_N_ case than that for other complexes, including pristine ones. Since for the C_B_–C_N_ defect, there exists a direct interlayer interaction of two carbon atoms; this result is in line with a higher stabilization energy per pair for the graphene bilayer [26] in comparison with the h-BN bilayer. A more detailed analysis of the stabilization energies and the presentation of the components of the SAPT(DFT) energy has been shifted to the Supplementary Information.

### Electronic spectra

**Fig. 2:**
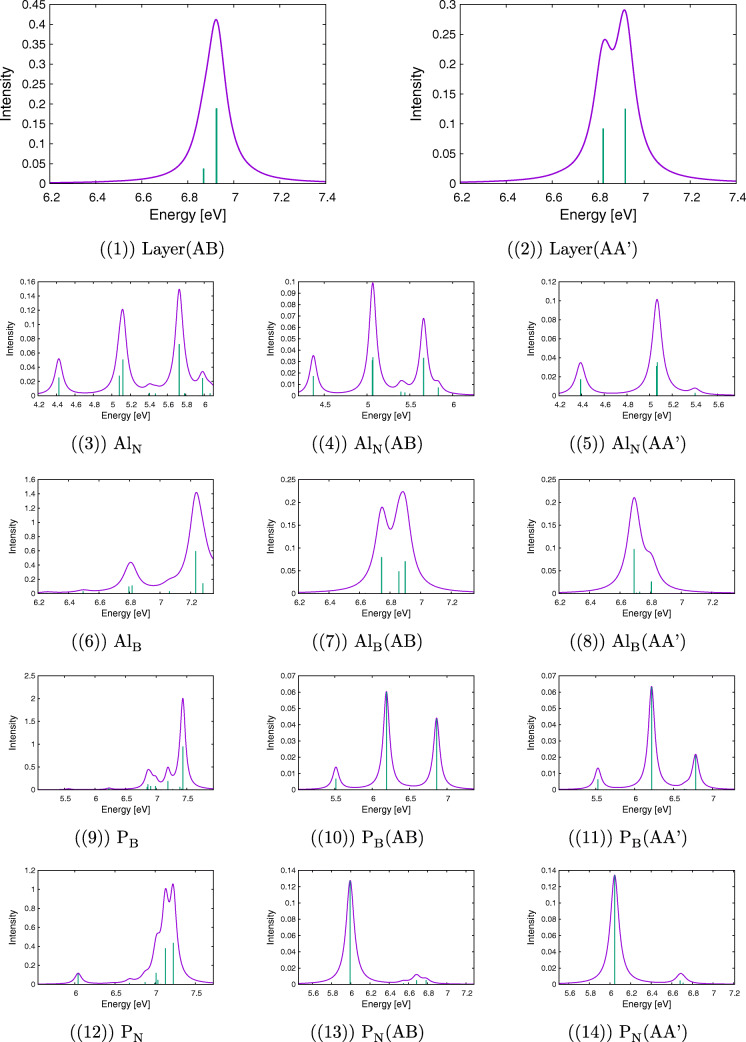

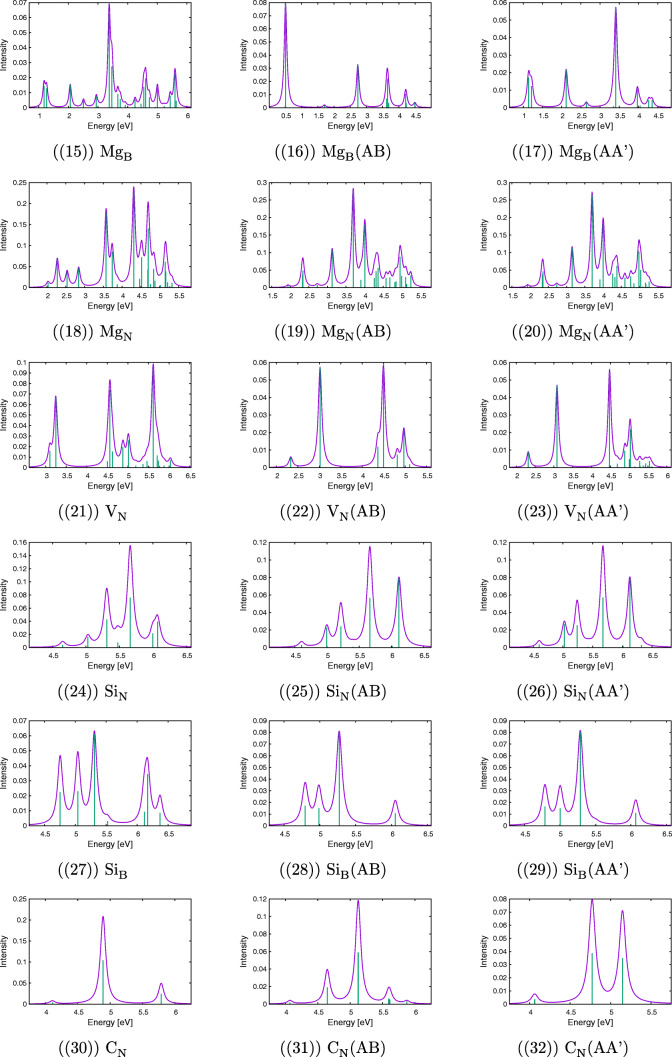

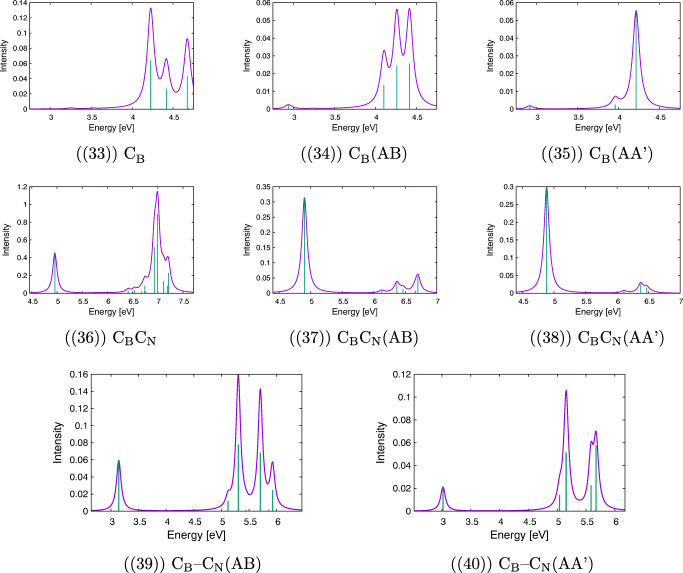
Simulated UV-Vis spectra for the cluster (right) and its complex with another cluster calculated with the modified CAM-B3LYP functional in the jun-cc-pVDZ basis set

Calculated electronic spectra for h-BN bilayers together with spectra for monolayers for defects under study are presented in Fig. 2. A comparison of spectra obtained for clusters and for AA’ or AB complexes allows making various predictions about possible changes in lines’ positions, their intensity, as well as changes in the excitation character, which are caused by interactions between clusters. Of special interest are differences between AA’ and AB stackings, which for the undoped case originate from two mechanisms. On the one hand, the electron density per atom has a lesser overlap with its counterpart on the second layer for the AA’ complex because clusters are farther from each other in this case. On the other hand, only every second atom in the AB complex experiences a significant overlap with the atom from the second layer because of the layer shift in the AB stacking. For the case of doped complexes, modifications of complexes’ geometry and a different number of electrons in comparison with the undoped case introduce the next level of complication in the spectra. Therefore, in view of these (often opposing) effects, it is difficult to predict *a priori* which effect will dominate dimers’ spectra. It should be noted, however, that the comparative analysis of SAPT(DFT) exchange components (see the Supplementary Information) strongly suggests that the AA’ stacking provides an overall higher electron density overlap relative to the AB stacking.

New features of electronic excitations resulting from point defects of h-BN may be explained as resulting from several factors. First factor is the number of valence electrons of the dopant atom, which can be the same or different (smaller or larger) than the number of valence electrons of the replaced atom. In the latter case, one obtains the *p*- or *n*-type defects, respectively. (Note parenthetically that among the considered defects there are some exotic defects, like a *p*-type defect in place of boron, Mg_B_, or a defect, which differs by more than one valence electron from the replaced atom, like Mg_N_ and P_B_.)

For the *p*-type, one can formally treat the process of doping as a removal of one electron from the old HOMO, followed by a spin-unrestricted relaxation of orbitals with help of the self-consistent field (SCF) procedure, which gives a new singly occupied HOMO and a low-energy LUMO. Therefore, one awaits the presence of several low-energy excitations which involve these orbitals (especially the LUMO). Quite on the contrary, for the *n*-type defect, the excess electron is placed on the old LUMO, and although the SCF adapts the orbitals to the new situation, no low-energy excitations are expected in this case. The second factor is the distortion from planarity of the h-BN lattice around defects, which creates an extra possibility for a separation of defect-localized excitations. This effect can be seen as the local character of not only the *p*_*z*_-type orbital of the defect, but also of the *p*_*x*_ and *p*_*y*_ orbitals, which are parallel to the h-BN plane. Such a spatial separation may result in an emergence of strictly or partially local excitations involving these orbitals. Possible spatial symmetry breaking caused by a defect is a third factor, which may contribute to e.g. changes in the energetic order of orbitals, which in turn may translate into modifications of the character and energetics of electronic excitations.

If the second layer is added below the doped layer, additional factors appear, contributing to the already complicated picture of the excitations. Firstly, the orbitals of the bilayer can be partially delocalized over both layers, so if main configurations utilize these orbitals, changes of the energy and character of excitations are expected. Secondly, if the second layer causes a partial restoration of the planar character of the first (doped) layer, a larger mixing (i.e., delocalization) of the dopant and the lattice orbitals can be observed than for the monolayer. Finally, the geometry and symmetry distortion may be different for the monolayer, the AA’ or the AB stacking, which may result in a different orbital order for these three situations.

#### Pristine layers

Let us start the analysis of the electronic states from the undoped clusters and complexes (see Fig. 2(1)–(2)). For the B_18_*N*_19_*H*_15_ cluster, the lowest excited state has the excitation energy equal to 6.4 eV. However, this transition is dipole-forbidden, similarly to the first excitation of 6.6 eV for the B_19_*N*_18_*H*_15_ cluster. Both values agree with the h-BN energy gap of 6 eV [5], especially after a systematic blueshift of the CAM-B3LYP-mod functional with respect to more accurate theories is taken into account (see test calculations for the borazine dimer in the Supplementary Information). The first excitation with nonzero intensity is twofold degenerate and has the excitation energy equal to 6.9 and 7.0 eV for these two clusters, respectively. As expected, it has the $\pi \rightarrow \pi ^{*}$ character, with both occupied and virtual orbitals delocalized over the whole cluster. The spectrum of a dimer formed from B_19_*N*_18_*H*_15_ and B_18_*N*_19_*H*_15_ contains both these lines, but they are redshifted in comparison with the cluster lines. This shift depends on the cluster type AA’ or AB, namely, it amounts to 0.06 (AA’) or 0.05 (AB) eV for the higher line and by 0.06 (AA’) or 0.01 (AB) eV for the lower one. Relative intensities of these lines also depend on the stacking type. The perusal of the first ten states of both complexes reveals that for the AB case there are several states of a significant charge transfer (CT) character from one cluster to the other, while the character of the analogous excitations for the AA’ complex is inherently local. It should be noted that already the first excited state of the AB complex has a partial CT character.

#### Al_N_ defect

The Al_N_ defect is twice-electron deficient (the III group element replaces the V group one); therefore, one can expect some unusual features in the spectra resulting from “freeing” the HOMO. In fact, several low-energy excitations have been obtained from the TD-DFT calculations in this case. These excitations form several groups placed in the 1.6 eV, 4.4 eV, 5.1 eV, and 5.4 eV energy regions (see Fig. 2(3)). This pattern is repeated for bilayers with small systematic redshifts of excitation energies (see Fig. 2(4) and (5)). The largest difference between mono- and bilayers occurs for the first excited state, where both AA’ and AB energies are redshifted by 0.07 eV, while for other states such differences are smaller than 0.05 eV. Therefore, concluding from these—rather small—energetic changes, one could assume that the second layer plays a secondary role in the Al_N_-induced spectrum. However, a different picture arises from the analysis of the excitation character of these states. First, one should note that the position of the Al atom above the h-BN surface prevents mixing of its 3*p*_*x*_ and 3*p*_*y*_ orbitals with the 2*p*_*x*_ and 2*p*_*y*_ orbitals of lattice atoms (here and in the following the *z* axis is assumed to be perpendicular to the layer) and leads to appearance of new types of excitations, which can be crudely characterized as promotions from or into the 3*p*_*m*_ (m=x,y,z) orbitals of Al. In a fact, these orbitals constitute the major part of the new HOMO and HOMO− 1 (3*p*_*x*_ and 3*p*_*y*_), and LUMO (3*p*_*z*_). In particular, the first excited state for the Al_N_ defect is dominated by the promotion from the HOMO or HOMO− 1 into the LUMO. This doubly-degenerate state lies only 1.64 eV above the ground state, but because of its character (e.g., HCode $\mathrm {p_{x}}\rightarrow \mathrm {p_{z}}$, which is forbidden in the C_3*v*_ point group), it has zero intensity.

Also, the next single-cluster state (4.41 eV), characterized as the excitation from 2*p*_*z*_ of neighbor nitrogen atoms to LUMO, is dipole-forbidden, and the first double-degenerate transition, which is dipole-allowed (4.43 eV), can be described as a promotion from HOMO or HOMO− 1 to some higher virtual orbital, which is localized in the AlB_3_ group and represents a mixture of a lone pair on Al and orbitals from the closest boron atoms. The same order of states around 4.4 eV is preserved in the AA’ complex, but for the AB case the nondegenerate state lies higher in energy than two degenerate states. The order of the next group of three energetically close-lying states (5.1 eV) does not change upon complexation, and these states can be described as excitations either from the HOMO/HOMO− 1 or to the LUMO. Both lines are visible in the spectrum, but the oscillator strengths of two degenerate states (which are of higher energy) decrease for bilayers at cost of the lower state from this group. It should be noted that also the last two calculated excitations of 5.41 eV make use of the HOMO/HOMO− 1 in their dominant configuration functions.

Even more interesting features can be found from the analysis of the CT numbers (Ω) obtained from the transition density partitioning [46]. This analysis allows to conclude that the two lowest states are not sensitive to the second layer, since the respective CT numbers are similar to each other independently of the stacking type. This fact can be explained by a constatation that the major part of the excitation occurs outside the layer structure. For higher states, more differences between these two stacking types start to appear, however. First, one can note that the sum of Ω_*ij*_ inside the doped layer is smaller for the AB stacking than for the AA’ one for the excitations around 4.4 eV. For the next group (5.1 eV), the situation is more complicated, since the CT number is smaller for the first excitation from this group (0.28 for AB and 0.40 for AA’), but larger for the doubly-degenerate excitation (0.79 for AB and 0.75 for AA’). Therefore, although energy differences between stacking types are small, one can observe in some cases significant modifications of the excitation character.

#### Al_B_ defect

The Al_B_ defect is nonplanar, too, but for this case the number of valence electrons is the same for the dopant and the leaving atom; therefore, no changes in energetic structures are expected which would result from a removal of electrons and the following rearrangement as in the case of Al_N_. In a fact, for both mono- and bilayers, the lowest excitation energies start above 6 eV. Let us analyze the spectrum of the single cluster in the first step (see Fig. 2(6)). It has the doubly-degenerate first excitation with the excitation energy of 6.26 eV (with a small, but nonzero intensity), which can be described as a promotion from a delocalized *π*-type HOMO/HOMO− 1 to a LUMO, which in turn is mostly represented by the 3*p*_*z*_ orbital of Al. The next state of the excitation energy of 6.50 eV is completely localized since it is dominated by the transition from HOMO− 3 to LUMO, where the HOMO− 3 is a *π* type orbital centered on Al and three surrounding N atoms. This state is dipole-allowed and has an even larger intensity than the lower state. Next few states, starting from the state of the 6.54 eV excitation energy, are represented by promotions from delocalized occupied to delocalized virtual orbitals. Finally, two degenerate states above 6.5 eV of high intensity correspond to lowest dipole-allowed states for the undoped B_19_*N*_18_*H*_15_.

A completely different picture arises for the electronic excitations of bilayers (see Fig. 2(7) and (8)). The first difference is the absence of the two lowest excitations, which in the monolayer case were characterized as promotions to the 3*p*_*z*_ (Al). The latter orbital for the bilayer case is involved into bridging the aluminium atom and the nitrogen atom from the second layer. The calculated states of bilayers usually represent various promotions from and to delocalized orbitals within one cluster, and none of them is localized in the vicinity of the defect. Quite the opposite, they rather resemble the states of the undoped h-BN. For instance the first excited state of energy equal to 6.44 eV (AA’) or 6.49 eV (AB) has zero intensity, similarly to next several states of energies of about 6.5–6.7 eV. For the AB complex, a partial CT character can be found in some states according to the transition density analysis. First, dipole-allowed excitations appear at 6.69 and 6.81 eV (AA’) or 6.74 and 6.90 eV (AB). All these states resemble the corresponding AA’ or AB undoped cases. However, a more detailed analysis of the excitation character reveals that the influence of the Al_B_ defect appears through the selection of the cluster within the complex, which undergoes the excitation. For instance, if the first five excited states of the AA’ undoped layer are compared with these for the Al_B_-doped complex, it turns out that in the former case excitations are limited to the same cluster for states from second to fifth, while for the latter case the third and fourth excited states occur in the different layer than the second excitation.

#### P_B_ defect

The P_B_-doped cluster has a HOMO localized on the PN_3_ unit, from which originates several excitations, including the three lowest ones of 5.57, 6.23, and 6.27 eV (see Fig. 2(9)). The former two states have nonzero intensities, so they appear as two lines in the monolayer spectrum. However, the promotion of electron occurs to delocalized virtual orbitals; therefore, these states are not fully localized on the defect. Among the calculated states, one can also identify the states corresponding to the lowest excited states of the undoped h-BN. These are states with the excitation energies of 6.64 eV and 6.86 eV, for which the promotion of the electron originates from and into delocalized *π*-type orbitals. The first from these excited states with a zero oscillator strength corresponds to the first excited state of the undoped monolayer, while the second (twofold degenerate) state has a large intensity and corresponds to the 6.9 eV line discussed above for pristine layers. Interestingly, for the P_B_ monolayer, there is a state with almost the same energy (6.85 eV) and with a nonzero intensity which is dominated by excitations from the HOMO.

For both types of bilayers, the first four excited states have the same character as for the monolayer, i.e., they represent an excitation from the HOMO localized in the vicinity of the dopant atom to delocalized virtual orbitals from the same layer. Their excitation energies are slightly (by at most 0.06 eV) redshifted in comparison with the monolayer (see Fig. 2(10) and (11)). Starting from the fifth excited state, more differences between both bilayer cases begin to appear. For the AA’ stacking, the dipole-forbidden 6.62 eV excitation is still limited to the doped layer, while for the AB case this state has an energy lower by 0.14 eV and has a partial CT character. Finally, for both bilayer cases, one can recognize a high peak at about 6.8 eV, corresponding to the dipole-allowed excitation of the undoped h-BN.

#### P_N_ defect

The case of the phosphorus replacing nitrogen is analogous to the Al_B_ defect, since it provides the same number of valence electrons as the replaced atom. Again, the cone shape of the defect allows for a partial isolation of the 3p orbitals of the phosphorus atom and three orbitals (HOMO, HOMO− 2, HOMO− 3) represent a mixture of one from the 3*p*_*m*_ orbitals of phosphorus and orbitals placed on three neighboring boron atoms. The two lowest excitation energies for the P_N_-doped cluster (6.04 and 6.07 eV) correspond to three locally excited states (two of them are degenerate), which can be characterized as excitations from one of these orbitals into the LUMO+ 8, localized on the PB_3_ unit (see Fig. 2(12)). The third excitation energy of 6.29 eV corresponds to a state dominated by the excitation from a delocalized HOMO− 1 into the same virtual orbital. Among higher states, one can mention a dipole-allowed local excitation (6.68 eV) from the HOMO to localized virtual orbitals resembling higher *p*_*x*_ and *p*_*y*_ orbitals of phosphorus.

Spectra of bilayers in both AB and AA’ stacking (see Fig. 2(13) and (14)) are quite similar to the monolayer P_N_ case, which does not mean that there is no differences in the excitation character for mono- and bilayers. For instance, many orbitals of the AA’ and AB complexes are to some extent delocalized to the second layer; therefore, some states acquire a partial CT character. Nevertheless, the three lowest excited states have the same character as in the PB_18_*N*_18_*H*_15_ cluster; i.e., they represent the promotion from the orbital dominated by one from 3p orbitals of phosphorus to a virtual orbital localized on the PB_3_ unit. However, contrary to the monolayer case, the state with the large intensity becomes the second in energy, while two degenerate states corresponding to the excitations from 3*p*_*x*_ or 3*p*_*y*_ of phosphorus are now the lowest energetically. Additionally, a redshift of the whole group is different for AB stacking (0.1 eV) and for the AA’ stacking (0.05 eV). One can also find the analog for the next excited state, which is redshifted by 0.06 eV for the AB stacking, but which has an unchanged excitation energy for the AA’ stacking (6.28 eV). For higher states, other differences start to appear between the AA’ and AB stackings, which can be at best seen by transition density analysis. It turns out that dark states from fifth to sevenths in the AB case are partially CT (from the doped into undoped cluster), while the corresponding states for the AA’ stacking involve only undoped (the fifth state) or doped (the latter two states) cluster. Finally, for both stacking types, one can find characteristic dipole-allowed excitations at 6.68 eV, which are not shifted with respect to the monolayer.

#### Mg_B_ defect

The Mg_B_ spectra of the cluster and both AB and AA’ complexes are the richest in low-energy lines from all the studied cases (see Fig. 2(15)–(17)). The Mg atom provides one valence electron less than boron; therefore, one obtains an unusual p-type boron-replacing defect. This means that the doubly occupied HOMO of boron is replaced by a singly occupied orbital and a new low-energy LUMO. Because of the cone shape of the defect, the three closest nitrogen atoms are elevated above the h-BN surface in the cluster, which prevents their 2*p*_*m*_ orbitals to mix effectively with other orbitals of this type; therefore, the HOMO and LUMO are constructed mostly from the orbitals of three N atoms forming a triangle around the Mg atom. Since it turns out that all the calculated states represent excitations to the LUMOβ, the local character of this orbital is of major importance in understanding the spectrum pattern in the presence of the Mg_B_ defect. An examination of the simulated spectra shows that the coarse position and intensity pattern of lines are quite similar for the cluster and the AA’ dimer. However, for the AB complex, the situation is quite different: while in the former case, the first two lines appear for the energy slightly above 1 eV and are followed by lines at about 2 eV, for the AB case the first excited state of a high intensity is observed already at 0.5 eV and the remaining lines are also placed in a completely different way as for the single cluster and the AA’ complex. The solution of this puzzle is provided by the observation of the form of the HOMOβ and LUMOβ in these cases. It turns out (see the figures in the Supplementary Information) that the shape of the HOMOβ of the cluster and the AA’ complex resembles the most the LUMOβ of the AB complex and vice versa: the LUMOβ of the cluster and the AA’ complex resembles the HOMOβ of the AB complex. Since all the excitations occur, as already noted, to the LUMOβ, the change of the character of this orbital explains a dramatic change in the low-energy part of the AB spectrum. The reason of this energetic order shift can be traced down to geometry differences between the cluster and the AA’ complex on the one side, and the AB complex on the other. In the former case, the two equidistant Mg-N bond lengths are shorter by about 0.04–0.06 Å than the remaining Mg-N bond, while for the AB complex they are longer by 0.06 Å.

#### Mg_N_ defect

The Mg_N_ defect introduces an even larger disbalance in numbers of valence electrons around the defect site. As noticed in the geometry section, the result of this disbalance is a lack of binding between Mg and two out of three boron atoms. The analysis of orbitals shows that there are several orbitals localized in the vicinity of the defect, playing a major role in the lowest electronic excitations. For instance, the HOMOβ resembles the *σ* bonding orbital between Mg and B, while HOMO*α* and LUMOβ, the antibonding counterpart of that orbital. As can be expected from the orbital analysis, the first excited states for the Mg_N_ defect for both mono- and bilayer cases (see Fig. 2(18)–(20)) have low excitation energies and can be characterized as electron promotions from the HOMO*α* (e.g., the first two states) of from the HOMOβ (e.g. the third state). The first and third excitations are completely localized on the defect, since in these cases the electron jumps either into the LUMO*α* (localized in the Mg-B bond and the remaining boron atoms around the N-hole) or into the LUMOβ for the first and third excitations, respectively. The second layer does not modify the character of these states, but it contributes to a redshift in the excitation energies (e.g., from 2.03 to 1.93–1.94 eV, from 2.28 to 2.33 eV, and as much as from 2.54 to 2.34–2.35 eV, respectively, for the first three excited states). The intensity of these states is also similar for mono- and bilayer cases. (It should be noted parenthetically that some heavily spin-contaminated states have been removed from the simulated spectra.)

#### V_B_ and V_N_ defects

The lowest excitation energies for the V_B_ defect are equal to about 0.9 eV for the cluster and both complexes. All calculated excited states for the V_B_ defect have zero or very small intensity. One should note, however, that the spin-unrestricted TD-DFT procedure for this case leads to a quite large spin contamination for all calculated states; therefore, the quality of this calculation is questionable [45] and will be not discussed any further.

Simulated spectra for the V_N_ defect are presented in Fig. 2(21)–(23). The lowest excitations of the cluster containing the V_N_ defect originate from the HOMO*α*, which is located on the B_3_ triangle left after the removal of the nitrogen atom. Some higher excitations have a different character: they promote an electron to the LUMOβ, which is similar in shape to the HOMO*α*. Most of these excitations have nonzero oscillator strengths, which makes them visible in the simulated spectrum, like e.g. the lowest two lines (3.10 and 3.25 eV). These lines correspond to excitations into delocalized virtual orbitals, which can be distinguished by their shape in the B_3_ region: for the lower excitation, this is a contribution from the 2*p*_*z*_ orbital on one boron (the one separated from the remaining pair), while for the upper excitation – a *σ*-like orbital between the pair of bound boron atoms. For the bilayers, these two lines are much lower in energy (2.32 eV and 3.08 eV for AA’ and 2.33 eV and 3.02 eV for AB stackings, respectively). This lowering in energy results apparently from a delocalization of target virtual orbitals. A more detailed analysis of these orbitals reveals that the lower state of the cluster corresponds to the higher state of the complex and vice versa, since the diffuse orbital for the upper (lower) excitation for the complex contains a contribution from 2*p*_*z*_(B) (*σ* (B-B)) orbital, on the contrary to the character of the corresponding cluster orbitals. In the 4–5-eV energy range, there are four more states for a cluster and five for AA’ and AB complexes. From these states, the highest two states for the cluster represent the excitations into the LUMOβ and one of them (4.62 eV) is fully localized in the vicinity of the defect (it originates from the HOMOβ, which represents a network of 2*p*_*z*_ orbitals of nitrogen atoms encircling the hole). For the complexes, all states below 4.9 eV can be characterized as excitations from the HOMO*α*. Therefore, the addition of the second layer causes a change in the states’ character in some cases. These differences can be explained by partial restoration of the planar character of the doped cluster through the stabilizing effect of the second cluster.

#### Si_N_ defect

The silicon atom introduces one unpaired electron into the cluster and forms the p-type or n-type defect for the Si_N_ and Si_B_ cases, respectively. For the former defect, all calculated excitations are dominated by promotions to the LUMOβ. Note that a similar situation occurs for the Mg_B_ defect, which is of the p-type, too. The LUMOβ mostly comprises the 3*p*_*z*_ orbital of Si, while degenerate HOMOβ and (*HOMO* − 1)β are mostly composed of the 3*p*_*x*_ and 3*p*_*y*_ orbitals of the same atom, respectively. The lowest double-degenerate excitation of 1.66 eV of the cluster can be described as a promotion from the HOMOβ or (*HOMO* − 1)β into the LUMOβ. However, these states, as well as the next excitation of 3.98 eV, which is also localized in the vicinity of the defect, have zero oscillator strengths, so the first visible lines in the UV-Vis spectrum appear at 4.65 eV and 5.03 eV and represent excitations from the 2*p*_*z*_ network of nitrogen atoms into the LUMOβ (see Fig. 2(24)). The addition of the second layer plays a minor role for the Si_N_ defect, as can be seen from Fig. 2(25) and (26). The excitation energies become somewhat lower (0.08 eV for the lowest state and the 0.05 eV for the first state of nonzero intensity), but the character of these excitations does not change and according to the transition-density analysis all these states involve excitations within the doped cluster only. Interestingly, on the orbital level some modifications do occur, e.g., the (HOMO− 3)β and (HOMO− 4)β switch their energetic order for the AA’ and AB stacking, but this change does not affect the excitation character of the third state, since for the AB stacking the excitation is characterized by the transition from the (HOMO− 4)β, which in this case resembles the (HOMO− 3)β for the cluster and for the AA’ complex. It should be noted that in the analysis of the Si_N_ defect, we have to skip states with too large spin contamination.

#### Si_B_ defect

Similarly to other defects possessing an extra valence electron, the Si_B_ defect introduces electronic states with much higher excitation energies than it is common for defects with one valence electron less than in the removed atom (see Fig. 2(27)–(29)). The examination of the orbital structure shows that a new singly occupied HOMO*α* and a LUMOβ appear, which are composed of a 4s orbital of silicon with a large admixture of 2*p*_*z*_ orbitals of the closest lattice atoms. The lowest excitation (4.76 eV) is characterized as the promotion of the electron from delocalized orbitals composed of 2*p*_*z*_ orbitals of nitrogen atoms to the LUMOβ, while the next two excitation energies (5.04 eV and 5.31 eV) correspond to excitations from the HOMO*α* into some delocalized virtual orbitals. All these lines are visible in the spectrum. The addition of the second layer changes the character of the first (doubly degenerate) state to partial CT (exceptionally from the undoped to the doped cluster and for both AB and AA’ stacking), but preserves the character of the lowest states in general. For both cases, the first excitation is blueshifted by 0.03–0.04 eV and the second and third ones—redshifted by 0.03–0.05 eV, and there is a small difference (about 0.01 eV) between the AA’ and AB stacking.

#### C_N_ defect

All calculated excitations for the C_N_ defect in the monolayer promote into the LUMOβ, similarly to other cases of the X_N_ type. The LUMOβ represents mostly the 2*p*_*z*_ orbital of carbon with a large admixture of the 2*p*_*z*_ orbitals of the three closest boron atoms. The lowest excitation energy is equal to 2.77 eV and it corresponds to a doubly degenerate state, which represents an excitation from two isoenergetic orbitals (HOMO− 6 and HOMO− 7) describing mostly *σ*-type C-B bonds, but also extending over closest B-N bonds. (Note that the planarity of the C_N_ defect facilitates such extension of the orbital, contrary to the cone-shaped defects, like Si_N_.) Since these orbitals involve a combination of 2*p*_*x*_ and 2*p*_*y*_ orbitals of carbon, the abovementioned states can be regarded as analogs of the lowest states for the Si_N_ defect. The second excitation energy of 3.45 eV corresponds to the excitation from the 2*p*_*z*_ orbitals on nitrogens around the carbon defect, also similarly to the Si_N_ case. All these states have zero intensity, and the first line visible in the spectrum occurs for 4.11 eV (see Fig. 2(30)). Altogether in the region from 4 to 5 eV one finds three excitation energies resulting from five states, namely: two degenerate states at 4.11 eV, one state at 4.75 eV, and another twofold degenerate state at 4.89 eV. The next state for the cluster is 0.9 eV higher (5.79 eV). All these states are characterized by electron promotions from combinations of 2*p*_*z*_ orbitals on nitrogen atoms, and the latter two have the highest intensities.

When the second undoped layer is added to the C-doped cluster, the lowest two excitation energies are not affected. Interestingly, two orbitals, from which the main excitation occurs for the lowest excitation energy, change their energetic order for AA’ and AB cases, but this does not affect the character of these excitations (i.e., always the orbital with a “proper” character is selected in the main configuration, irrespective of its energetic order, similarly to the silicon case). In the region above 4 eV, several new features of electronic spectra can be observed, which clearly result from the addition of a new layer (see Fig. 2(31) and (32)). First of all, the two degenerate states of energy 4.89 eV for the monolayer become significantly lower in energy for the bilayer case, which results in change of the energetic order of states between 4 and 5 eV. The redshift of 0.12 eV (AA’) and 0.25 eV (AB) is enough to make them lower in energy than the 4.75 eV state of the cluster, which is only slightly redshifted (by 0.03 eV to 4.72 eV) for the AB stacking or blueshifted (by 0.02 eV to 4.78 eV) for the AA’ stacking in the complexes. The large shift in energy in the former case can be justified by the interaction with the second layer; i.e., these states acquire a partial CT character. Even more interesting is the appearance of another low-lying pair of degenerate states with the excitation energies of 5.14 eV (5.12 eV) for the AA’ (AB) stacking with a large oscillator strength. Also, in this case, the excitation originates from occupied orbitals, which have a large component in the second layer.

#### C_B_ defect

For the C_B_ defect, all ten calculated states but the last one are zero- or low-intensity states (oscillator strengths 0.005 and lower). The character of the excitations for the C_B_ defect can be regarded as an opposite to the C_N_ case: while for all calculated excited states of C_N_ the electron is promoted to the LUMOβ; in the C_B_ case all the excitations originate from the same HOMO*α*. The HOMO*α* is localized on the CN_3_ unit (similarly to the LUMOβ of C_N_, also localized close to the dopant atom) and is composed of the 2*p*_*z*_ orbitals of these four atoms with the opposite signs for the carbon and the surrounding nitrogen atoms. Therefore, differences between these excitations are solely determined by properties of virtual orbitals. The lowest excited state of the doped cluster is dominated by the promotion to a virtual orbital delocalized over the whole molecule. The symmetry of this orbital prohibits the use of the 2*p*_*z*_ orbitals and its diffuse character can explain why this excitation is very sensitive to the addition of the second layer (see Fig. 2(33)–(35)): while for the single cluster, its excitation energy is equal to 2.59 eV, it rises over 0.3 eV for the complex to 2.92 eV (AA’) and 2.94 eV (AB). Additionally, its oscillator strength becomes somewhat larger in presence of the second layer. A comparison of the remaining excited states of a cluster and both complexes results in several surprising observations. For instance, for the second excited state, the excitation energy practically does not change (3.11, 3.10, 3.13 eV for the single cluster and AA’ and AB complexes, respectively), which suggests that this excitation is limited to the doped layer. However, a more detailed examination of the dominant configuration shows that this is not the case: for the cluster, the respective virtual orbital is composed of evenly distributed 2*p*_*z*_ orbitals on boron atoms, while for both complexes this orbital is delocalized over both layers, which indicates a partial CT character of this state. The same change in the state character occurs for the next state, which is doubly degenerate, but here additionally the AA’ stacking leads to a 0.08 eV redshift with respect to the cluster excitation energy to 3.17 eV, while the AB stacking leaves this energy almost unchanged. Unfortunately, this shift cannot be observed in the spectrum because of a zero oscillator strength for this state. Interestingly, for a cluster there is a state of 3.52 eV, for which one cannot find a counterpart if another layer is added. The next doubly degenerate state, which has a diffuse character, is blueshifted by 0.09 eV and by 0.21 eV (from 3.75 eV) for the AB and AA’ stacking cases, and for the latter case it acquires a nonzero oscillator strength. Therefore, in this case, the addition of the second layer results in emergence of an additional line in the spectrum, but only for the AA’ stacking. Next, the state with the excitation energy of 4.02 eV for the cluster can be characterized as an excitation into the framework of 2*p*_*z*_ orbitals on boron atoms. The AA’ stacking affects neither the energy (4.01 eV), nor the character of the state (the virtual counterpart is limited to the cluster), but the AB stacking, although it does not change the energy, modifies the character of the state, which becomes partially CT. A similar situation occurs for the 4.22 eV state (for the cluster) and 4.20 eV state (AA’) which also have a diffuse character. Interestingly, no counterpart of this state can be found for the AB stacking. Finally, the last pair of excited states, which have the highest intensity so far for all three cases, should be discussed. The excitation (4.23 eV) goes into the framework of 2*p*_*z*_ orbitals of boron atoms for the case of the monolayer. However, for both bilayer cases, the excitation of the similar excitation strength and energy has a completely different character—the respective dominant virtual orbitals are very diffuse and are delocalized over both layers.

#### C_B_C_N_ defect

The spectrum of the C_B_C_N_ defect is dominated by the lowest excitation, which has the oscillator strength of at least two orders of magnitude larger than other calculated excitations, as can be seen in Fig. 2(36)–(38). This excitation is dominated by the promotion of electron from the HOMO localized on the CN_2_ unit (with larger contribution of C_N_) into a virtual orbital, which is also localized on the CN_2_, but this time with a predominance of C_B_. The excitation energy of 4.96 eV becomes redshifted for complexes: by 0.08 eV for AA’ and by 0.06 eV for AB. Additionally, although the character of the HOMO orbital, from which the excitation mostly occurs, is preserved, the virtual orbital, to which the electron is promoted, changes significantly in the presence of the second cluster. One can see that this orbital becomes delocalized over both layers, and while for the AB layer one still observes some localization on the CN_2_ unit, almost no such localization remains for the AA’ case. (It should be noted parenthetically that the value for the monolayer differs from 4.83 eV obtained with the original CAM-B3LYP used in Ref. [32], which—as explained in the theory section—is a price for introducing of a larger ratio of exact exchange in the CAM-B3LYP-mod, needed to model faithfully the CT interaction in complexes.) Many higher excited states also are dominated by the electron promotion from the HOMO in most cases. If the number of calculated states is extended, one can additionally see a set of lines above 6.9 eV, which have a higher intensity than the localized excitation discussed above for the monolayer, while for both types of bilayers they become lower than the local excitation.

#### C_B_–C_N_ defect

Finally, let us discuss the C_B_–C_N_ defect, where the C_B_ and C_N_ defects reside in neighboring layers one directly above another. In this case, a dipole-allowed CT excitation appears as the lowest excitation in the spectrum with the energy of 3.02 eV and 3.14 eV for the AA’ and AB clusters, respectively (see Fig. 2(39) and (40)). In both cases, the corresponding excitation is dominated by the electron promotion from the HOMO to LUMO, where the HOMO is the *π*-type orbital localized on C_N_, while the LUMO is of the *π* type, too, but is localized on C_B_. Therefore, this state is a pure CT state, which is confirmed by the analysis of the transition density matrix (the transition density occupation numbers are equal of 0.8 for the term from the C_N_ to the C_B_ cluster). This first state is followed by several other states originating from the HOMO, which are however more than 1 eV higher in energy. Interestingly, the shape of the C_B_–C_N_ HOMO is similar to the LUMOβ of the C_N_ defect, while the shape of its LUMO resembles closely the HOMO*α* of the C_B_ defect.

## Summary and conclusions

We performed a theoretical study of mono- and bilayers of the doped h-BN with an emphasis on differences between properties of a doped monolayer and a system built from one doped and one undoped layer. The layer was modelled by molecular clusters of 37 or 42 “heavy,” i.e., non-hydrogen atoms. We utilize several high-level theoretical methods, like DFT, SCS-MP2, SAPT(DFT), and TD-DFT, for calculations of (i) geometry modifications resulting from the introduction of defects and/or the addition of the second layer, (ii) relative energetic stability of two types of bilayers (AA’ and AB stacking), and (iii) the electronic emission spectra of mono- and bilayers.

The analysis of geometries of the systems under study leads to several important conclusions. Firstly, the dopant atom for the monolayer can either make the h-BN surface nonplanar (for the majority of considered cases), or it can fit into the h-BN plane. For the former case, two types of plane distortion are possible: either a cone with the dopant atom on its top is formed, or the plane is folded around the dopant atom. Among the considered point defects, the C_N_, C_B_, C˙BC˙N, C_B_–C_N_, and V_B_ ones are planar or almost planar, the Al_B_, Al_N_, Si_B_, Si_N_, Mg_B_, P_B_, and P_N_ have a cone shape, while only two V_N_ and Mg_N_ trigger the folding of the plane. The second layer facilitates the reconstruction of the planar character of the h-BN surface, although this effect is never strong enough to completely restore the planarity of the doped h-BN. The restoration of the planarity is especially pronounced for two folding cases.

When the second layer is added, in most cases the defect is placed outwards with respect to the second layer, but in several cases it points inwards, i.e., between the layers. From all the defects formed by III-row elements, the Mg and Al atoms are the most interesting, since for the Mg_B_ and Al_B_ defects the dopant atom goes to the interlayer space, while the Mg_N_, Al_N_, Si, and P defects all have the dopant atom placed outwards the layers. There are also cases, for which a different behavior is observed for the AA’ and AB stacking types, like for the V_N_ and Mg_B_ defects. In the former case, one boron atom around the hole points either inwards (AA’) or outwards (AB) the second layer, while for the latter case a triangle formed by nitrogen atoms surrounding the defect is either obtuse (AA’), or acute (AB).

The higher stability of AA’ with respect to the AB stacking persists for all defects under study, but the energetic difference between the AA’ and AB type becomes smaller for two cases where the defect points inwards the second layer (Mg_B_ and Al_B_) and for the C_B_–C_N_ defect, where in the latter case the result agrees with the reversed stability order of bilayers of graphene (AB more stable than AA).

The conclusions on the electronic excited states persisting for most of the calculated cases are the following. For the X_B_ defects, the lowest excitations usually excite from the HOMO, and for the X_N_ defects into the LUMO, which are localized in the vicinity of the defect. For the n-type defects, the lowest excitation energies are as a rule higher than for the p-type. However, only a few states were found to be fully localized in the vicinity of a defect (for the one-atom defects such localized states were found only for the Mg_N_ case, what is directly related to the double-hole character of this defect). A common effect arising from the addition of the second undoped layer is a redshift of the excitation energies related to the doped layer, although in some cases, like C_B_, also blueshifts are observed. This redshift is as a rule of thumb not larger than 0.1 eV in most cases, but there are exception to this rule, like the V_N_ defect (about 0.8 eV for the lowest excited state). Within the bilayers the AB-type stacking has a greater tendency for CT excitations from one layer to the other, but the delocalization of orbitals exists for both types of bilayer.

Among interesting effects for individual defects, one can name: (i) the absence of the lowest excitation of the monolayer in the bilayers’ spectra for the Al_B_ defect, which can be explained by the fact that this defect points inwards the layers; (ii) acquiring of nonzero intensity by the 3.75 eV excited state through the interaction with the second layer for the C_B_ case; (iii) the localization of some states on the defect for the Mg_N_ case; (iv) the dramatic difference of the excitation pattern between the AA’ and AB bilayers for the Mg_B_ defect, which can be explained by the subtle energy-order modification of some orbitals resulting from geometrical changes around the defect; (v) the existence of similar energetic-order modifications for several other cases (like Si_N_), however, it does not translate into the spectra modifications, as in the Mg_B_; (vi) the appearance of additional excitation of about 5.1 eV as the results of layers’ interaction for the C_N_ defect.

Such modifications of the spectra triggered by the presence of the second layer show that in the experiment the question whether the measured h-BN is a true monolayer or multiple-layer can be of utmost importance in order to understand the spectra of doped h-BN.

## Electronic supplementary material

Below is the link to the electronic supplementary material.

(PDF 1.20 MB)

(ZIP 3.16 MB)

## References

[1] Pouch JJ, Alterovitz SA (1990). Materials science forum.

[2] Novoselov KS, Geim AK, Morozov SV, Jiang D, Zhang Y, Dubonos SV, Grigorieva IV, Firsov AA (2004). Science.

[3] Lin Y, Williams TV, Connell JWJ (2010). Phys Chem Lett.

[4] Zhang K, Feng Y, Wang F, Yang Z, Wang JJ (2017). Mater Chem C.

[5] Cassabois G, Valvin P, Gil B (2016). Nat Photon.

[6] Tran TT, Bray K, Ford MJ, Toth M, Aharonovich I (2015). Nat Nanotechnol.

[7] Attaccalite C, Bockstedte M, Marini A, Rubio A, Wirtz L (2011). Phys Rev B.

[8] Solozhenko VL, Will G, Elf F (1995). Solid State Commun.

[9] Krivanek OL (2010). Nature.

[10] Silly MG, Jaffrennou P, Barjon J, Lauret J-S, Ducastelle F, Loiseau A, Obraztsova E, Attal-Tretout B, Rosencher E (2007). Phys Rev B.

[11] Jin C, Lin F, Suenaga K, Iijima S (2009). Phys Rev Lett.

[12] Meyer JC, Chuvilin A, Algara-Siller G, Biskupek J, Kaiser U (2009). Nano Lett.

[13] Du XZ, Li J, Lin JY, Jiang HX (1110). Phys Appl Lett.

[14] Uddin MR, Li J, Lin JY, Jiang HX (2107). Phys Appl Lett.

[15] Du XZ, Uddin MR, Li J, Lin JY, Jiang HX (2102). Phys Appl Lett.

[16] Majety S, Cao XK, Li J, Dahal R, Lin JY, Jiang HX (1110). Phys Appl Lett.

[17] Da̧browska AK, Pakuła K, Bożek R, Rousset JG, Ziółkowska D, Gołasa K, Korona KP, Wysmołek A, Stȩpniewski R (2016). Acta Phys Pol A.

[18] Mokkath JH, Schwingenschlȯgl UJ (2014). Mater Chem C.

[19] Reimers JR, Sajid A, Kobayashi R, Ford MJJ (2018). Chem Theory Comput.

[20] Bourrellier R, Meuret S, Tararan A, Stėphan O, Kociak M, Tizei LHG, Zobelli A (2016). Nano Lett.

[21] Koronski K, Kaminska A, Zhigadlo ND, Elias C, Cassabois G, Gil B (2019). Superlattice Microst.

[22] Rydberg H, Dion M, Jacobson N, Schröder E, Hyldgaard P, Simak SI, Langreth DC, Lundqvist BI (2003). Phys Rev Lett.

[23] Marom N, Bernstein J, Garel J, Tkatchenko A, Joselevich E, Kronik L, Hod O (2010). Phys Rev Lett.

[24] Hsing C-R, Cheng C, Chou J-P, Chang C-M, Wei C-M (2014). J Phys.

[25] Xie W, Tamura T, Yanase T, Nagahama T, Shimada T (2018). Jpn J Appl Phys.

[26] Podeszwa RJ (2010). Chem Phys.

[27] Mostaani E, Drummond ND, Fal’ko VI (2015). Phys Rev Lett.

[28] Jeziorski B, Moszynski R, Szalewicz K (1994). Chem Rev.

[29] Szalewicz K (2012). Wiley Interdiscip Rev Comput Mol Sci.

[30] Heßelmann A, Jansen G (2002). Chem Phys Lett.

[31] Misquitta AJ, Szalewicz K (2002). Phys Chem Lettx.

[32] Korona T, Chojecki M (2019) Int J Quantum Chem:1–19

[33] Pelini T (2019). Phys Rev Mater.

[34] Meitei OR, Heßelmann A (2016). Chem Phys Chem.

[35] Lebedev AV, Lebedeva IV, Knizhnik AA, Popov AM (2016). RSC Adv.

[36] Grimme SJ (2006). Comput Chem.

[37] Grimme S, Antony J, Ehrlich S, Krieg HJ (2010). Chem Phys.

[38] Schäfer A, Horn H, Ahlrichs R (1992). Chem Phys.

[39] Schäfer A, Huber C, Ahlrichs R (1994). Chem Phys.

[40] Grimme SJ (2003). Chem Phys.

[41] Yanai T, Tew DP, Handy NC (2004). Phys Chem Lett.

[42] Koppen JV, Hapka M, Szczėṡniak MM, Chałasiṅski GJ (2012). Chem Phys.

[43] Dunning THJ (1989). Chem Phys.

[44] Papajak E, Zheng J, Xu X, Leverentz HR, Truhlar DGJ (2011). Chem Theory Comput.

[45] Ipatov A, Cordova F, Doriol LJ, Casida ME (2009). J Mol Struc Theo Chem.

[46] Plasser F, Lischka HJ (2012). Chem Theory Comput.

[47] Grüning M, Gritsenko OV, van Gisbergen SJA, Baerends EJJ (2001). Chem Phys.

[48] Adamo C, Barone VJ (1999). Chem Phys.

[49] Schäfer A, Horn H, Ahlrichs R (1992). Chem Phys.

[50] Helgaker T, Klopper W, Koch H, Noga JJ (1997). Chem Phys.

[51] Halkier A, Helgaker T, Jørgensen P, Klopper W, Koch H, Olsen J, Wilson AK (1998). Chem Phys Lett.

[52] Halkier A, Helgaker T, Jørgensen P, Klopper W, Olsen J (1999). Chem Phys Lett.

[53] Anoop A, Thiel W, Neese F (2010). Chem Theory Comput.

[54] Neese F, Valeev EFJ (2011). Chem Theory Comput.

[55] Rutkowska-Zbik D, Korona T (2012). Chem Theory Comput.

[56] Eichkorn K, Treutler O, Öhm H, Häser M, Ahlrichs R (1995). Phys Chem Lett.

[57] Eichkorn K, Weigend F, Treutler O, Ahlrichs R (1997). Theor Chem Acc.

[58] Frisch MJ et al (2016) Gaussian 16 Revision B.01. Gaussian Inc., Wallingford

[59] Werner H-J et al (2015) MOLPRO, version 2015.1, a package of ab initio programs, see http://www.molpro.net

[60] Plasser F (2016) THEODORE: A Package for Theoretical Density, Orbital Relaxation, and Exciton Analysis, Available from http://theodore-qc.sourceforge.net/

[61] Pėcharman A-F, Colebatch AL, Hill MS, McMullin CL, Mahon MF, Weetman C (2017). Nat Commun.

[62] MgB_2_ Crystal Structure: Datasheet from ’PAULING FILE Multinaries Edition – 2012’ in SpringerMaterials https://materials.springer.com/isp/crystallographic/docs/sd_1931851, Copyright 2016. Springer, Berlin & Material Phases Data System (MPDS), Switzerland & National Institute for Materials Science (NIMS), Japan

[63] Nagamatsu J, Nakagawa N, Muranaka T, Zenitani Y, Akimitsu J (2001). Nature.

[64] Yourdkhani S, Chojecki M, Hapka M, Korona T (2016). Phys Chem A.

[65] Cuscó R, Gil B, Cassabois G, Artús L (2016). Phys Rev B.

[66] Żuchowski PS, Podeszwa R, Moszyński R, Jeziorski B, Szalewicz K (2008). Chem. Phys.

[67] Korona T, Hesselmann A, Dodziuk HJ (2009). Chem Theory Comput.

[68] Hesselmann A, Korona T (2011). Phys Chem Chem Phys.

[69] Pitoňȧk M, Riley KE, Neogrȧdy P, Hobza P (2008). Chem Phys Chem.

