# Supplementary material for: Influence of the second layer on geometry and spectral properties of doped two-dimensional hexagonal boron nitride
Source: J Mol Model. 2020 Jul 27;26(8):216. doi: 10.1007/s00894-020-04456-8 (PMC7384999; doi:10.1007/s00894-020-04456-8)
Supplement: Supplementary file 1 — (PDF 1.20 MB) [file 894_2020_4456_MOESM1_ESM.pdf]

Supplementary Information: Influence of the second layer on geometry and spectral properties of doped two-dimensional hexagonal boron nitride

Michał Chojecki,† Ewa Lewandowska,‡ and Tatiana Korona*,†

†*Faculty of Chemistry, University of Warsaw, ul. Pasteura 1, 02-093 Warsaw, Poland*

‡*V LO im. księcia Józefa Poniatowskiego, ul. Nowolipie 8, 00-150 Warsaw, Poland*

* E-mail: tania@chem.uw.edu.pl

List of Figures

FS-3 Simulated IR spectra for complexes of two clusters, as described in the main text, calculated with the B97D3 functional in the DVP basis set. 11

FS-2 HOMOβ and LUMOβ molecular orbitals of the Mg_B layer as well as Mg_B(AB) and Mg_B(AA') clusters. The upper undoped layer has been removed from the figure. 11

List of Tables

TS1 Components of the SAPT(DFT) interaction energy for the complexes under study in the CBS limit. Energies are given in kJ/mol. 6

TS2 B97-D3/SVP optimized geometry of the Al_B(AB) 12

TS3 B97-D3/SVP optimized geometry of the Al_B(AA') 13

TS4 B97-D3/SVP optimized geometry of the Al_N(AB) 14

TS5 B97-D3/SVP optimized geometry of the Al_N(AA') 15

TS6 B97-D3/SVP optimized geometry of the Layer(AB) 16

TS7 B97-D3/SVP optimized geometry of the C_B(AB) . . . 17
TS8 B97-D3/SVP optimized geometry of the C_B-C_N(AB) . . . 18
TS9 B97-D3/SVP optimized geometry of the C_B-C_N(AA') . . . 19
TS10 B97-D3/SVP optimized geometry of the C_B(AA') . . . 20
TS11 B97-D3/SVP optimized geometry of the C_BC_N(AB) . . . 21
TS12 B97-D3/SVP optimized geometry of the C_BC_N(AA') . . . 22
TS13 B97-D3/SVP optimized geometry of the C_N(AB) . . . 23
TS14 B97-D3/SVP optimized geometry of the C_N(AA') . . . 24
TS15 B97-D3/SVP optimized geometry of the Mg_B(AB) . . . 25
TS16 B97-D3/SVP optimized geometry of the Mg_B(AA') . . . 26
TS17 B97-D3/SVP optimized geometry of the Mg_N(AB) . . . 27
TS18 B97-D3/SVP optimized geometry of the Mg_N(AA') . . . 28
TS19 B97-D3/SVP optimized geometry of the P_B(AB) . . . 29
TS20 B97-D3/SVP optimized geometry of the P_B(AA') . . . 30
TS21 B97-D3/SVP optimized geometry of the P_N(AB) . . . 31
TS22 B97-D3/SVP optimized geometry of the P_N(AA') . . . 32
TS23 B97-D3/SVP optimized geometry of the Layer(AA') . . . 33
TS24 B97-D3/SVP optimized geometry of the Si_B(AB) . . . 34
TS25 B97-D3/SVP optimized geometry of the Si_B(AA') . . . 35
TS26 B97-D3/SVP optimized geometry of the Si_N(AB) . . . 36
TS27 B97-D3/SVP optimized geometry of the Si_N(AA') . . . 37
TS28 B97-D3/SVP optimized geometry of the V_B(AB) . . . 38
TS29 B97-D3/SVP optimized geometry of the V_B(AA') . . . 39
TS30 B97-D3/SVP optimized geometry of the V_N(AB) . . . 40
TS31 B97-D3/SVP optimized geometry of the V_N(AA') . . . 41
TS32 B97-D3/SVP optimized geometry of the Al_B . . . 42
TS33 B97-D3/SVP optimized geometry of the Al_N . . . 43
TS34 B97-D3/SVP optimized geometry of the C_B . . . 44
TS35 B97-D3/SVP optimized geometry of the C_BC_N . . . 45
TS36 B97-D3/SVP optimized geometry of the C_N . . . 46

TS37B97-D3/SVP optimized geometry of the Mg_B 47
TS38B97-D3/SVP optimized geometry of the Mg_N 48
TS39B97-D3/SVP optimized geometry of the P_B 49
TS40B97-D3/SVP optimized geometry of the P_N 50
TS41B97-D3/SVP optimized geometry of the Si_B 51
TS42B97-D3/SVP optimized geometry of the Si_N 52
TS43B97-D3/SVP optimized geometry of the V_B 53
TS44B97-D3/SVP optimized geometry of the V_N 54
TS45B97-D3/SVP optimized geometry of the BN-Layer(N in center) 55
TS46B97-D3/SVP optimized geometry of the BN-Layer(BN in center) 56
TS47B97-D3/SVP optimized geometry of the BN-Layer(N in center) 57

Theoretical considerations on the accuracy of the modified CAM-B3LYP functional for local and charge-transfer excitations

The quality of the present functional for the bilayers of h-BN has been double-checked by performing calculations for the AA' stacked structure (the "sandwich" structure) of two borazine molecules. Additionally, two more advanced *ab initio* methods, algebraic adiabatic construction to the second order – ADC(2)[1] and equation-of-motion coupled cluster truncated to single and double excitations – EOM-CCSD,[2] have been used to obtain the spectrum for this complex with the same basis set. It turns out that the first excited state, as well as three lowest states with nonzero oscillator strengths are reproduced faithfully by the selected DFT functional. The excitation energy of the first excited state (which is dipole-forbidden) is equal to 6.79 eV, 6.37 eV, and 6.54 eV for TD-DFT, ADC(2), and EOM-CCSD methods, respectively, and the excitation character (performed by the analysis of orbitals taking part in the major electron promotions) confirms that all three methods describe the same state (i.e. that present TD-DFT produces no spurious CT states, which were reported whne some older functionals were utilized, see e.g. Ref.[3]). The difference between the EOM-CCSD and the CAM-B3LYP-mod results can serve as the estimation of a systematic energy blueshift of this functional. As one can see, for the lowest state this shift is equal to 0.25 eV, while for three dipole-allowed states it becomes 0.4 eV (doubly degenerate states with the 7.53, 6.84, and 7.15 eV excitation energy and one state with the 7.63, 6.91, and 7.23 eV for these three methods, respectively). The actual shift could be about 0.1 eV higher since the EOM-CCSD method usually tends to slightly overestimate excitation energies, contrary to ADC(2), which has a tendency to underestimate them. One can therefore assume that the error

resulting from the present calculations is about 0.35-0.5 eV. The character of the excited states, which are partially CT, assures that this type of states will be properly accounted for in the case of the h-BN dilayers with the CAM-B3LYP-mod functional.

The analysis of the energetic stability of complexes under study

The analysis of the stabilization energies of the complexes composed of the cluster with a defect and the cluster without a defect (presented in Table 1 of the main article), allows to make several interesting conclusions, the most important of which have been mentioned in the main article, like the greater stability of the AA'-stacked complexes with respect to the AB-stacked ones and a decrease of the difference between AA' and AB case for some cases, namely, for Al_B and Mg_B defects. For these cases both Al or Mg atoms are placed on top of the cone directed *towards* the second layer, while for the remaining cases the dopant atom resides outside the second layer or (for a single carbon defect) it (almost) does not distort the planarity of the h-BN surface. The change in stability of AB *versus* AA' for the dopant cone pointing towards the second layer can be explained by the fact that in this case also three neighbour nitrogen atoms are necessarily placed closer to the second layer and for the AA' stacking they seemingly are too close to the boron atoms of the second layer, while for the AB stacking these three atoms are placed "harmlessly" above the empty ring centers. For the remaining case of a diminished ΔE_{stab} (C_B-C_N) one can note that this observation agrees with a higher stability of the AB stacking for graphene. Therefore one can expect that a higher concentration of C_B-C_N defects could lead to an inversion of the AA' *versus* AB stability order.

Let us move to the discussion of the total SCS-MP2 stabilization energies. First one should emphasize that the complexes under study can be divided into three groups: (i) consisting of two closed-shell molecules, (ii) one closed-shell and one open-shell (spin doublet) molecule, (iii) two open-shell (both spin doublet) molecules. The first group is represented by undoped systems and by defects with Al and P, the third class: by the C_B-C_N case, while the remaining defects belong to the second case. As it can be expected, the lowest value of the stabilization energy (under –300 kJ/mol) occurs for the interaction of two open-shell molecules (the C_B-C_N defect). This case differs significantly in the interaction strength when compared to the next two cases with a low stabilization energy, for which the absolute value of this energy is over two times smaller. Nevertheless, also for these two cases, i.e. the Al_B (the energy around –140 kJ/mol) and the Mg_B (the energy around –120 kJ/mol) – the distance between the Al or Mg and the closest nitrogen atom from the second molecule of the complex is so small that one expects beginnings of the creation of covalent bonding between Al and N or Mg and N.

The systems under study are quite rigid because of the network of conjugated bonds, so their deformation energies are in most cases below 10 kJ/mol. There are several exceptions, though. The largest deformation effects occur for the Al_B and Mg_B defects, followed by C_B-C_N and V_N. Usually deformation energies for the AA' complexes are higher than for the AB counterparts, what can be explained by the fact that a smaller number of atoms are placed "face-to-face" for the latter type.

Next, one can compare the stabilization energies of the undoped and doped complex within the same stacking type, which shows the influence of a defect on a dilayer stability. The higher stabilization of the doped complex occurs for the C_B-C_N, Al_B, and Mg_B defects. This difference is especially pronounced for the C_B-C_N case, where two open-shell molecules interact with each other. The dopant atom has practically no influence on the stabilization energy differences for the C_B, C_N, and V_B defects, while for all other cases it causes a small destabilization of the complex (between 5 and 27 kJ/mol). The highest destabilization occurs for the V_N and Al_N defects, followed by Si_N and P_N ones. Therefore, the replacement of the electron-rich nitrogen atom seems to enhance the destabilization effect in comparison to the boron case.

Finally, for closed-shell clusters one can evaluate the relative importance of various SAPT components, which are presented in Table TS1. For all presented cases the dispersion energy is the most important attractive component of the interaction energy. The induction energy is almost always smaller in absolute value (with the exception of Al_B) and additionally it is mostly damped the exchange-induction counterpart. For the case of the Al_B defect the dominance of the induction can be explained by a partial bond formation between the Al atom and the N atom of the second layer. The net first-order contribution is always repulsive. It should be noted that the importance of dispersion contribution makes all DFT calculations of dilayers, which do not include a dispersion correction, highly unreliable. Additionally, one can notice a regular pattern in a behaviour of exchange energies, which are higher for the AA' than for the AB stacking. This behavior can be explained by a larger overlap of electron clouds for the former stacking.

Table TS1: Components of the SAPT(DFT) interaction energy for the complexes under study in the CBS limit. Energies are given in kJ/mol.

Complex	$E^{(1)}_{elst}$	$E^{(1)}_{exch}$	$E^{(2)}_{ind}$	$E^{(2)}_{exch-ind}$	$E^{(2)}_{disp}$	$E^{(2)}_{exch-disp}$	δE_{HF}	E^{SAPT}_{int}
Layer(AB)	−103.8	260.0	−95.5	91.3	−285.4	36.9	−12.0	−108.4
Layer(AA')	−140.8	291.5	−110.8	105.9	−293.3	39.6	−14.1	−122.0
Al_B(AB)	−245.1	422.2	−446.1	353.5	−306.4	47.6	−17.0	−191.1
Al_B(AA')	−302.4	491.7	−496.7	397.6	−332.6	54.1	−17.3	−205.7
Al_N(AB)	−86.0	226.7	−85.2	80.5	−248.8	30.7	−9.7	−91.7
Al_N(AA')	−126.3	263.0	−101.8	96.2	−262.7	34.2	−13.0	−110.2
C_BC_N(AB)	−111.8	282.2	−105.9	101.5	−317.4	41.3	−12.7	−122.9
C_BC_N(AA')	−157.7	327.3	−125.1	119.3	−334.7	45.1	−16.4	−142.1
P_B(AB)	−104.0	262.5	−99.5	94.9	−283.2	36.8	−12.8	−105.4
P_B(AA')	−143.8	296.6	−115.8	110.3	−292.0	39.5	−15.1	−120.2
P_N(AB)	−94.8	243.5	−90.2	85.9	−267.8	33.7	−10.7	−100.4
P_N(AA')	−135.0	279.6	−107.2	102.0	−279.3	36.9	−13.8	−116.9

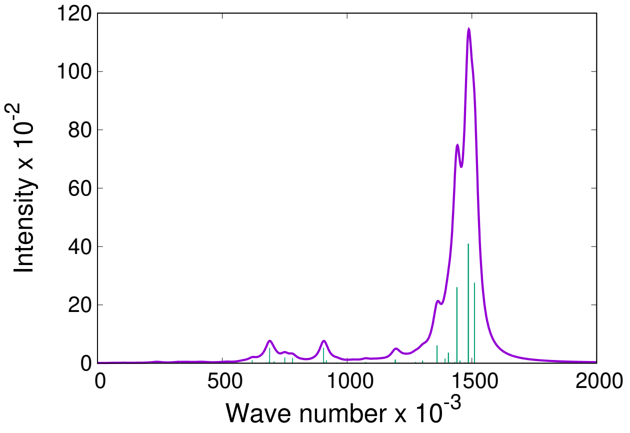

((1)) Al_B(AB)

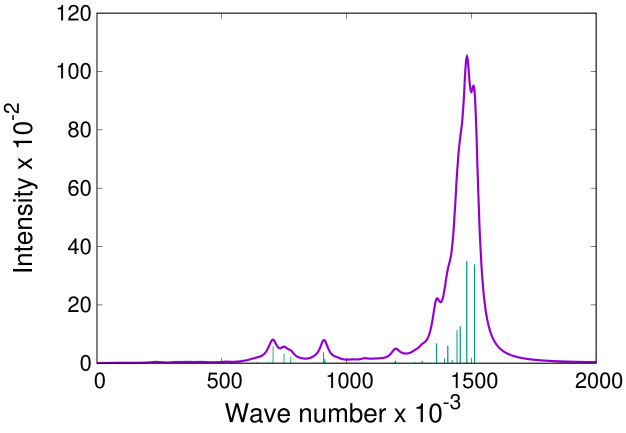

((2)) Al_B(AA')

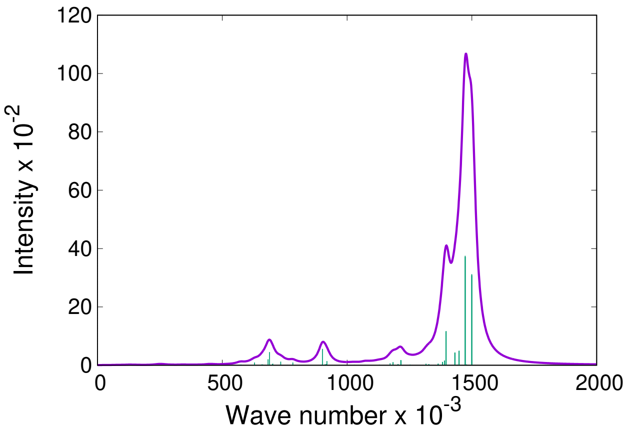

((3)) Al_N(AB)

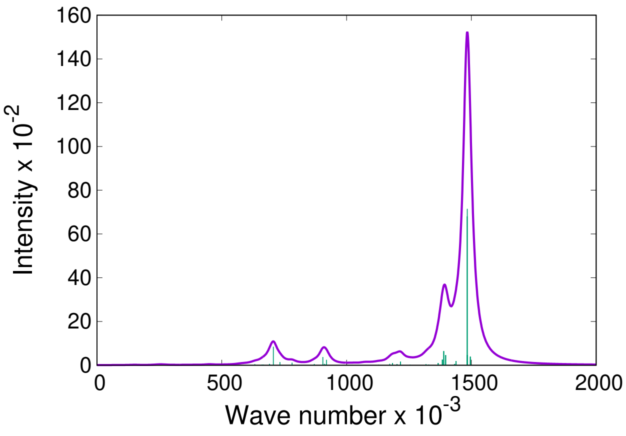

((4)) Al_N(AA')

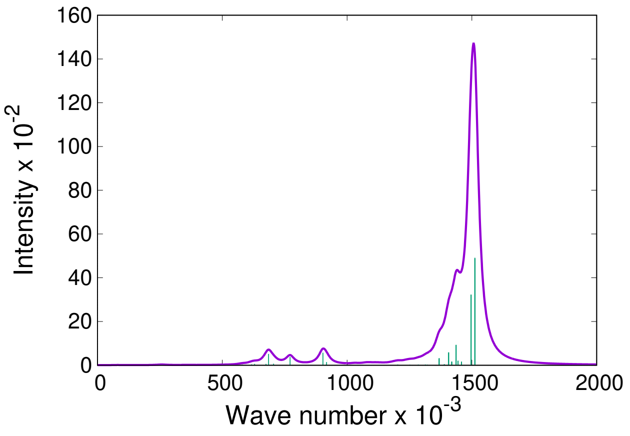

((5)) Layer(AB)

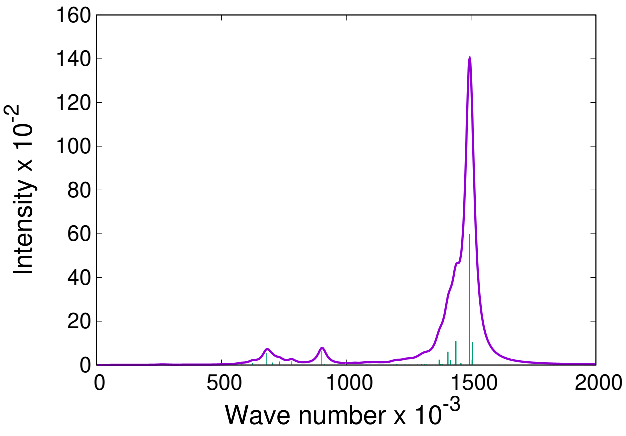

((6)) C_B(AB)

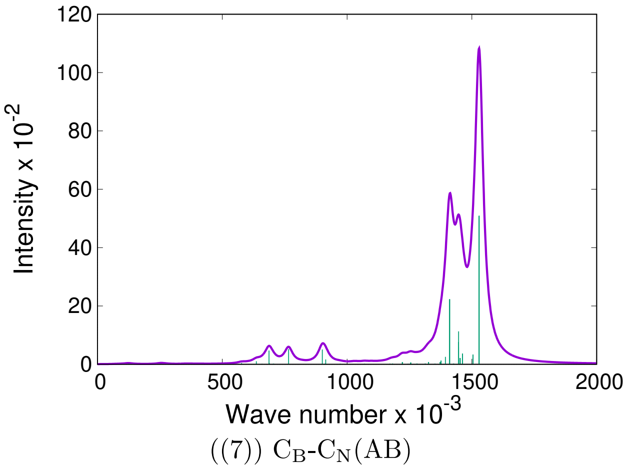

((7)) C_B-C_N(AB)

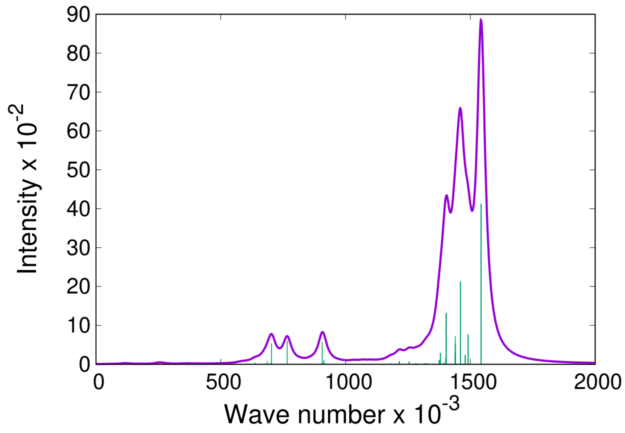

((8)) C_B-C_N(AA')

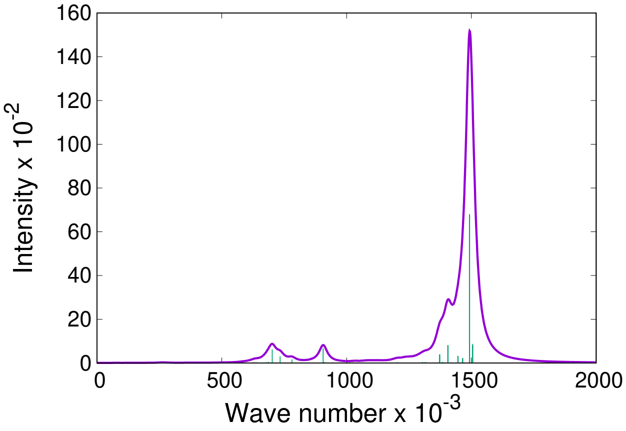

((9)) C_B(AA')

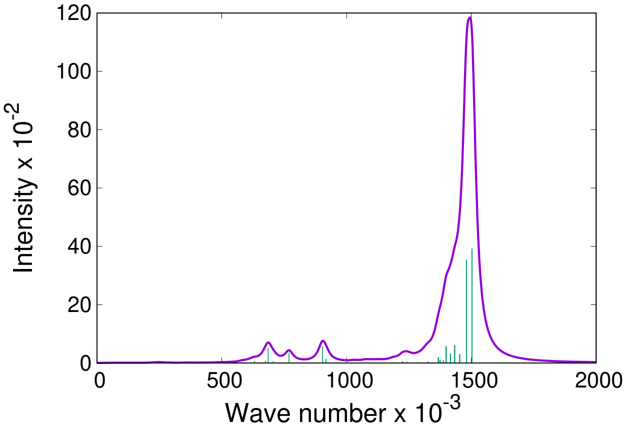

((10)) C_N(AB)

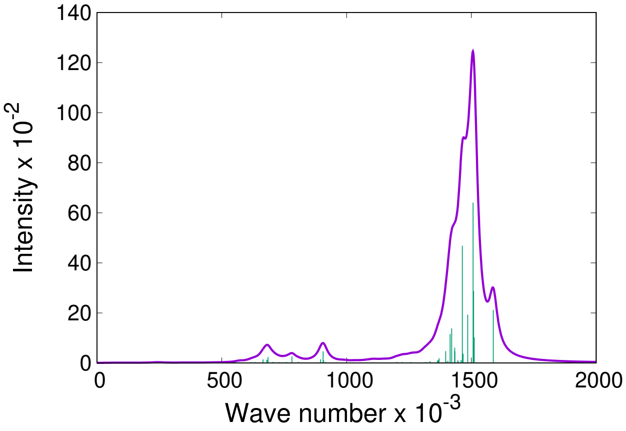

((11)) C_BC_N(AB)

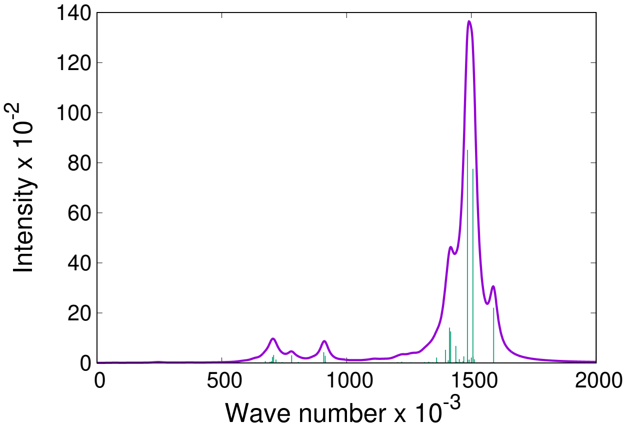

((12)) C_BC_N(AA')

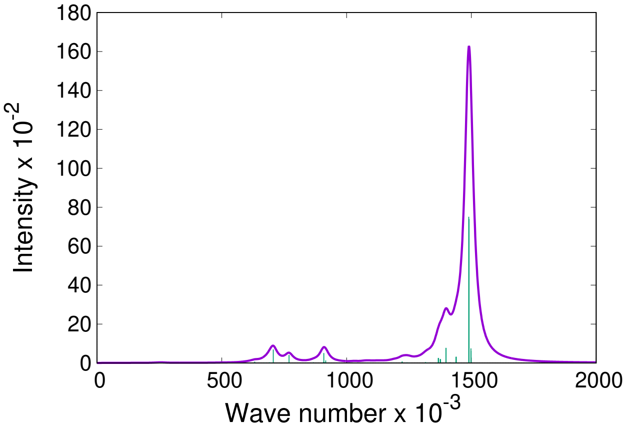

((13)) C_N(AA')

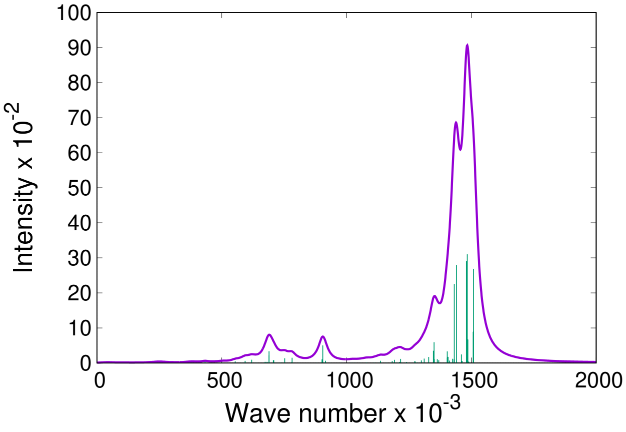

((14)) Mg_B(AB)

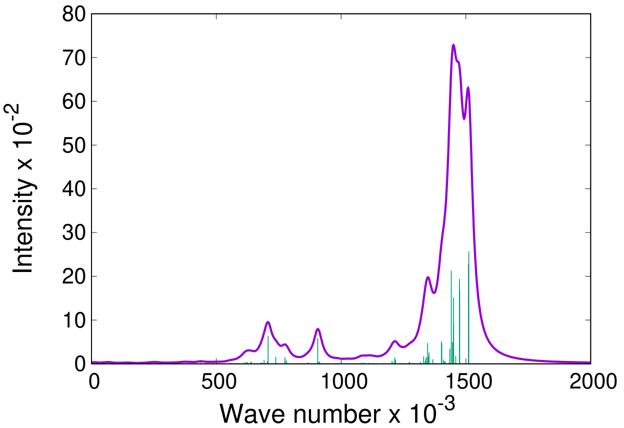

((15)) Mg_B(AA')

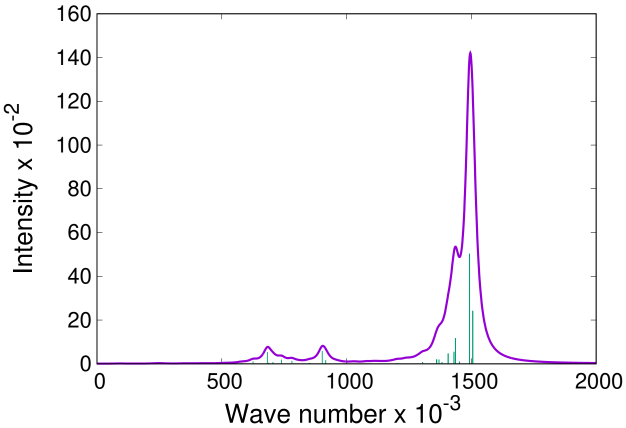

((16)) P_B(AB)

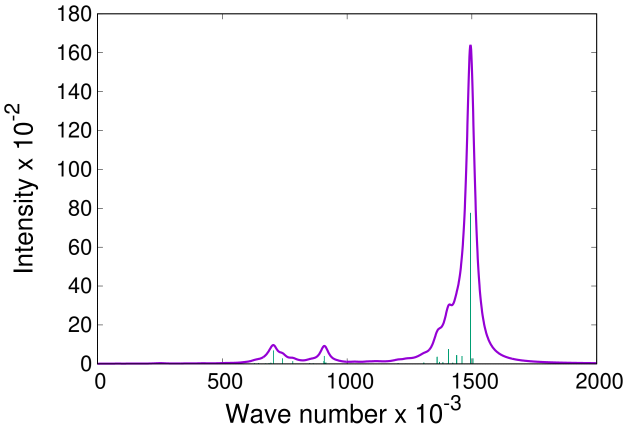

((17)) P_B(AA')

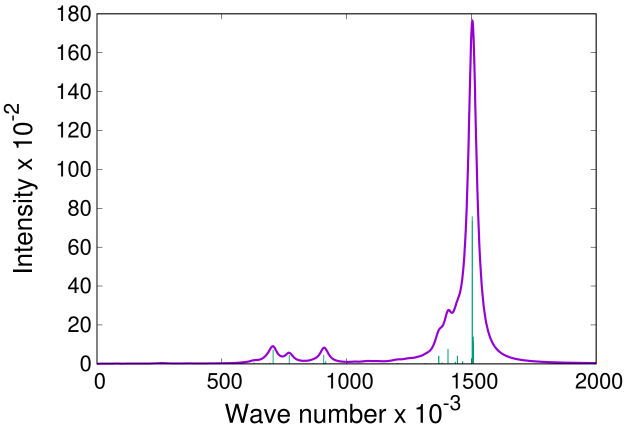

((18)) Layer(AA')

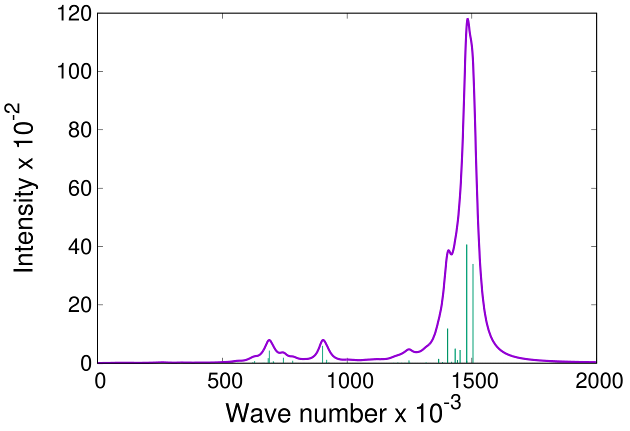

((19)) P_N(AB)

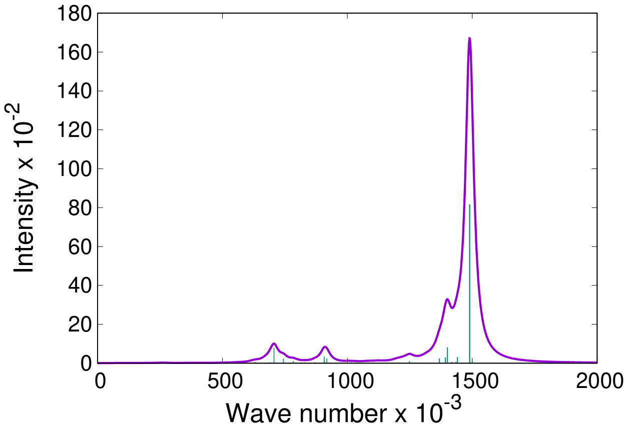

((20)) P_N(AA')

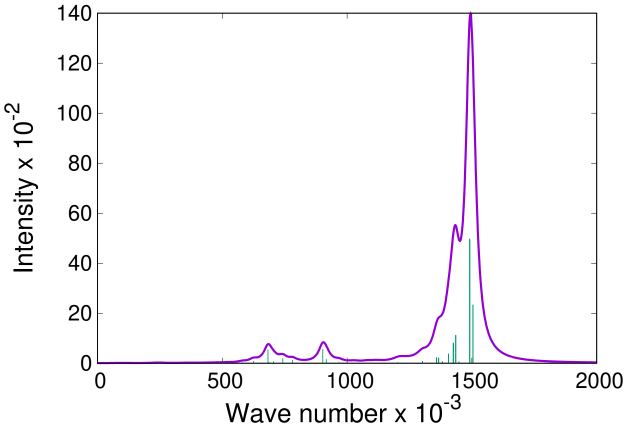

((21)) Si_B(AB)

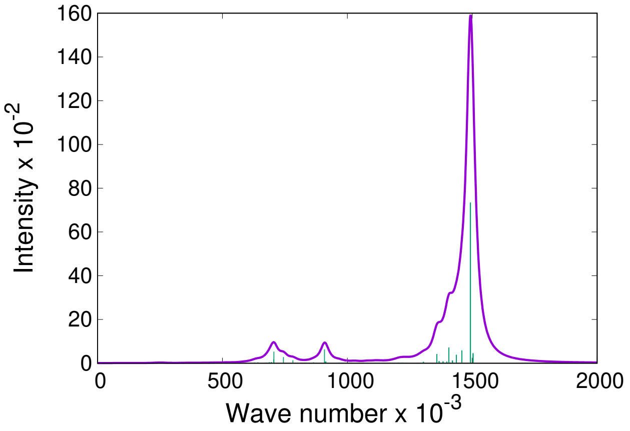

((22)) Si_B(AA')

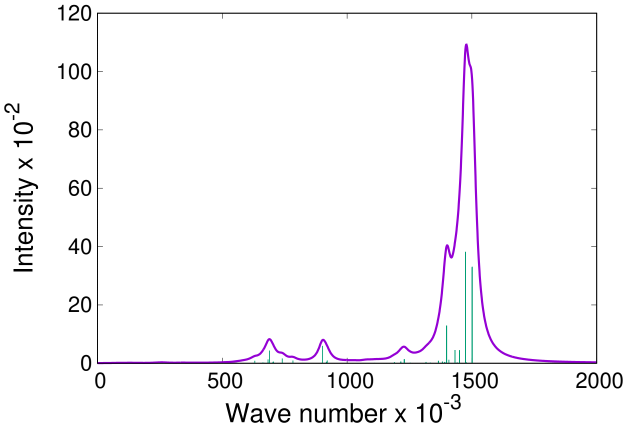

((23)) Si_N(AB)

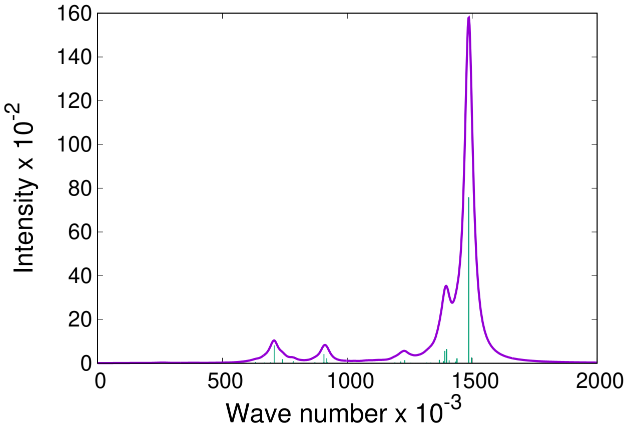

((24)) Si_N(AA')

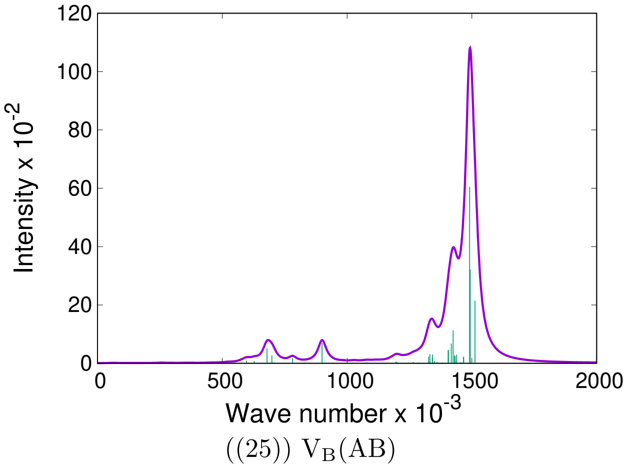

((25)) V_B(AB)

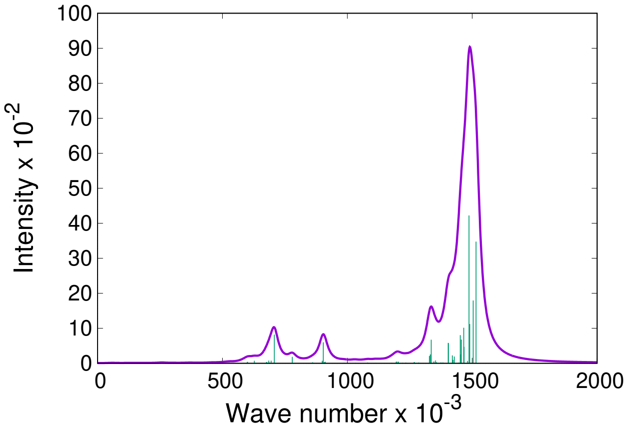

((26)) V_B(AA')

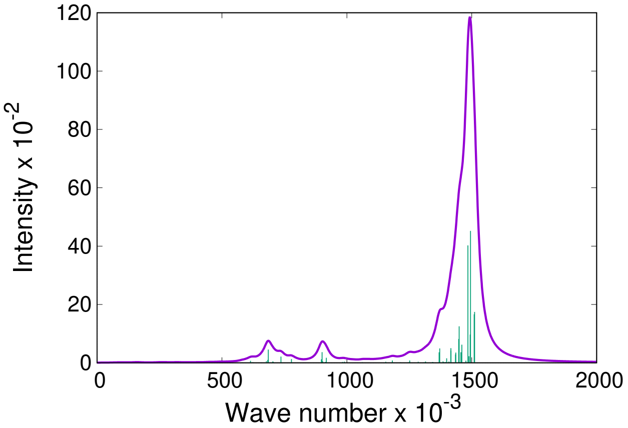

((27)) $V_N(AB)$

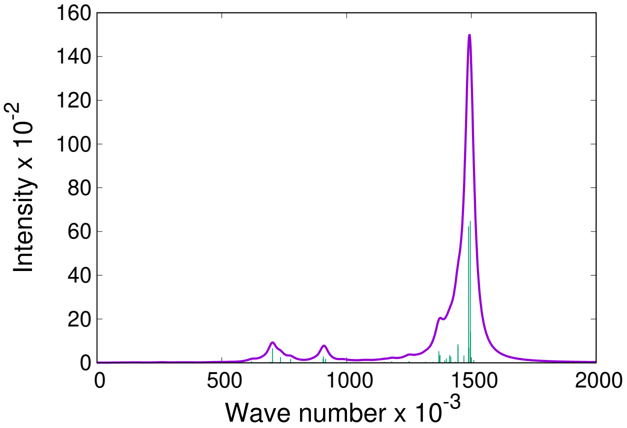

((28)) $V_N(AA')$

Figure FS-3: Simulated IR spectra for complexes of two clusters, as described in the main text, calculated with the B97D3 functional in the DVP basis set.

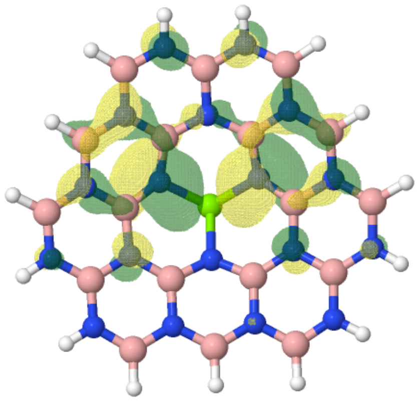

((1)) Mg_B HOMOβ

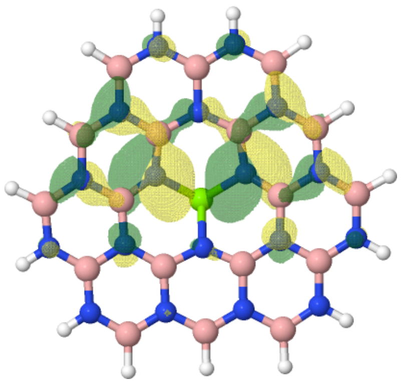

((2)) $Mg_B(AA')$ HOMOβ

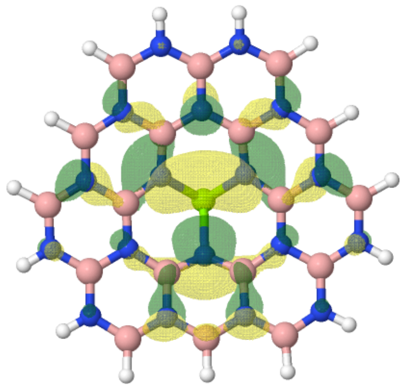

((3)) $Mg_B(AB)$ HOMOβ

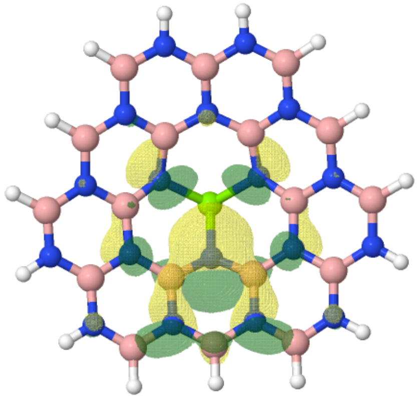

((4)) Mg_B LUMOβ

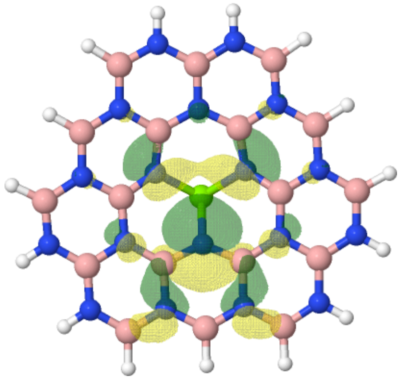

((5)) $Mg_B(AA')$ LUMOβ

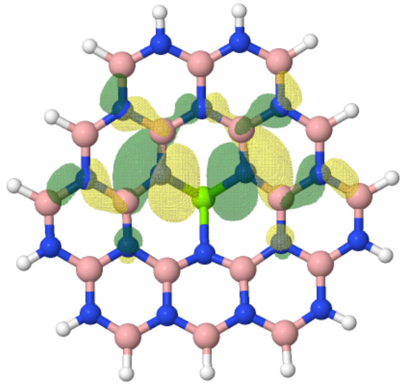

((6)) $Mg_B(AB)$ LUMOβ

Figure FS-2: HOMOβ and LUMOβ molecular orbitals of the Mg_B layer as well as $Mg_B(AB)$ and $Mg_B(AA')$ clusters. The upper undoped layer has been removed from the figure.

Table TS2: B97-D3/SVP optimized geometry of the Al_B(AB)

At.	X	Y	Z	At.	X	Y	Z
N	−4.929 09	−1.726 90	−1.840 98	B	−5.119 94	1.163 75	1.597 26
B	−3.645 09	−2.398 03	−1.732 16	N	−3.906 37	1.964 77	1.659 36
N	−3.536 87	−3.842 80	−1.840 76	B	−2.605 60	1.311 08	1.748 15
B	−2.287 17	−4.547 03	−1.897 36	N	−2.516 82	−0.145 55	1.800 13
N	−1.061 14	−3.782 96	−1.772 94	B	−3.742 40	−0.949 11	1.745 46
B	−1.138 80	−2.336 94	−1.501 97	N	−5.007 88	−0.261 52	1.661 25
N	0.096 91	−1.681 32	−1.206 99	H	−5.862 22	−0.806 62	1.587 20
Al	−0.001 00	−0.000 60	−0.635 51	N	−3.626 01	−2.382 99	1.748 52
N	1.404 78	0.924 05	−1.209 66	H	−4.471 72	−2.938 89	1.656 32
B	2.590 88	0.181 85	−1.503 18	B	−2.355 86	−3.058 60	1.745 60
N	2.607 21	−1.310 55	−1.529 30	N	−2.226 68	−4.492 93	1.661 25
B	1.400 55	−2.188 93	−1.502 15	H	−3.065 96	−5.060 98	1.587 45
N	1.491 68	−3.634 28	−1.772 41	B	−0.962 79	−5.161 15	1.597 06
B	2.798 36	−4.250 39	−1.897 02	H	−0.911 28	−6.368 03	1.496 86
N	3.957 74	−3.405 76	−1.838 71	N	0.253 93	−4.364 92	1.658 61
B	3.897 19	−1.958 18	−1.730 51	B	0.170 00	−2.911 68	1.747 73
N	5.094 39	−1.142 03	−1.838 18	N	−1.131 97	−2.252 39	1.800 28
B	5.079 34	0.292 31	−1.895 88	B	−1.240 42	−0.815 17	1.756 82
N	3.804 49	0.972 21	−1.772 86	N	0.001 12	0.000 78	1.537 28
B	3.687 56	2.425 75	−1.852 99	B	−0.084 59	1.484 17	1.754 97
N	2.398 83	3.108 39	−1.774 13	N	−1.383 52	2.108 97	1.797 85
B	2.279 28	4.548 01	−1.898 70	B	−1.469 76	3.571 86	1.741 29
N	0.967 89	5.129 50	−1.842 61	N	−2.776 67	4.176 99	1.657 17
B	−0.255 49	4.353 27	−1.733 97	B	−3.987 44	3.416 54	1.595 18
N	−0.171 49	2.912 55	−1.530 66	H	−5.058 42	3.975 25	1.494 64
B	−1.455 95	2.152 54	−1.502 14	H	−2.849 00	5.187 73	1.581 66
N	−2.747 17	2.808 27	−1.772 64	N	−0.249 55	4.333 96	1.742 84
B	−3.947 65	1.980 32	−1.851 92	H	−0.307 98	5.344 26	1.650 01
N	−3.894 47	0.523 04	−1.771 46	B	1.050 45	3.717 80	1.741 05
B	−5.081 24	−0.300 52	−1.897 15	N	1.133 59	2.254 71	1.797 16
H	−6.182 11	0.191 70	−2.047 34	B	2.439 58	1.603 36	1.746 13
B	−2.597 24	−0.120 64	−1.501 01	N	2.519 51	0.146 22	1.796 00
N	−2.439 48	−1.604 85	−1.528 91	B	1.328 93	−0.666 39	1.754 05
N	−1.505 97	0.754 69	−1.206 31	N	1.387 15	−2.106 55	1.797 07
H	−5.024 54	2.520 83	−2.004 88	B	2.695 76	−2.765 95	1.739 65
B	−2.795 66	4.251 95	−1.898 71	N	2.732 75	−4.205 63	1.655 23
N	−1.560 78	4.981 99	−1.842 84	B	1.554 34	−5.015 32	1.593 66
H	−1.606 03	5.988 34	−1.980 51	H	1.642 18	−6.220 06	1.492 98
H	−3.848 06	4.840 75	−2.048 54	H	3.631 84	−4.672 84	1.578 63
B	1.192 58	2.306 87	−1.504 07	N	3.879 39	−1.948 16	1.740 05
H	0.895 89	6.134 36	−1.979 87	H	4.783 42	−2.402 46	1.644 86
H	3.256 15	5.255 28	−2.047 60	B	3.829 61	−0.510 39	1.739 31
H	4.694 27	3.088 07	−2.005 03	N	5.007 18	0.318 86	1.655 65
H	6.115 68	0.909 35	−2.044 44	B	4.953 96	1.747 62	1.594 37
H	5.988 88	−1.605 88	−1.974 10	N	3.656 08	2.403 02	1.657 56
H	4.863 97	−3.845 97	−1.975 56	B	3.569 13	3.854 44	1.594 85
H	2.922 48	−5.449 82	−2.047 68	N	2.278 66	4.469 89	1.656 87
B	0.256 07	−4.408 70	−1.853 13	H	2.233 78	5.482 28	1.582 36
H	0.326 20	−5.611 46	−2.007 06	H	4.568 55	4.533 10	1.495 51
H	−2.270 79	−5.752 89	−2.046 71	H	5.973 39	2.395 86	1.495 13
H	−4.385 75	−4.385 35	−1.977 90	H	5.918 70	−0.123 65	1.579 38
H	−5.763 19	−2.291 76	−1.978 68	H	−6.207 33	1.689 89	1.497 07

Table TS3: B97-D3/SVP optimized geometry of the Al_B(AA')

At.	X	Y	Z	At.	X	Y	Z
N	−1.122 36	−5.106 14	−1.778 19	B	−1.116 43	−5.137 00	1.612 23
H	−1.082 48	−6.120 26	−1.836 79	H	−1.101 19	−6.348 33	1.545 46
B	0.125 27	−4.366 62	−1.702 99	N	0.124 32	−4.379 22	1.673 05
N	1.413 26	−5.033 81	−1.775 58	B	1.406 17	−5.065 21	1.613 57
H	1.431 35	−6.048 61	−1.833 42	H	1.459 74	−6.275 41	1.546 37
B	2.671 07	−4.345 03	−1.826 05	N	2.607 26	−4.287 93	1.648 92
H	3.709 15	−4.971 17	−1.919 48	H	3.493 31	−4.780 98	1.579 56
N	2.668 02	−2.895 58	−1.753 43	B	2.610 72	−2.845 68	1.700 87
B	3.893 52	−2.101 11	−1.803 49	N	3.817 09	−2.061 77	1.681 02
H	4.960 65	−2.677 12	−1.899 64	H	4.708 22	−2.542 98	1.592 76
N	3.885 17	−0.640 63	−1.753 62	B	3.811 14	−0.623 10	1.700 92
B	5.098 58	0.152 13	−1.826 27	N	5.015 33	0.170 83	1.649 46
H	6.191 58	−0.372 13	−1.920 54	H	5.913 68	−0.299 52	1.580 50
N	4.984 37	1.581 57	−1.774 99	B	5.006 33	1.601 44	1.613 08
H	5.842 75	2.123 22	−1.832 37	H	6.047 61	2.220 32	1.545 82
B	3.719 94	2.292 18	−1.701 37	N	3.729 68	2.297 07	1.672 61
N	2.485 93	1.531 86	−1.550 34	B	2.487 38	1.531 90	1.726 67
B	2.600 69	0.044 18	−1.527 73	N	2.521 51	0.073 23	1.754 28
N	1.477 93	−0.797 17	−1.262 06	B	1.308 53	−0.707 04	1.690 40
B	1.390 65	−2.197 47	−1.527 78	N	1.321 15	−2.149 28	1.754 60
N	0.083 96	−2.917 86	−1.550 71	B	0.082 76	−2.920 77	1.726 89
B	−1.261 55	−2.273 07	−1.529 60	N	−1.197 57	−2.221 06	1.753 30
N	−2.497 02	−3.043 16	−1.755 22	B	−2.445 42	−2.989 69	1.698 32
B	−2.417 45	−4.490 34	−1.828 04	N	−2.359 87	−4.429 32	1.645 88
H	−3.418 09	−5.174 72	−1.921 75	H	−3.216 31	−4.972 03	1.575 55
B	−3.765 94	−2.320 23	−1.805 39	N	−3.694 41	−2.275 67	1.677 89
H	−4.798 45	−2.956 43	−1.900 31	H	−4.556 59	−2.806 78	1.588 48
N	−3.841 24	−0.861 82	−1.754 40	B	−3.770 35	−0.839 02	1.699 46
B	−2.597 85	−0.104 55	−1.529 15	N	−2.522 43	−0.070 47	1.753 52
N	−1.428 74	−0.880 10	−1.263 92	B	−1.267 02	−0.780 51	1.689 21
Al	0.000 35	0.000 25	−0.687 22	N	−0.000 28	−0.000 53	1.470 12
N	−0.047 69	1.678 16	−1.263 83	B	−0.042 78	1.486 31	1.689 54
B	1.208 65	2.303 05	−1.528 15	N	1.199 89	2.218 51	1.753 24
N	1.174 72	3.758 58	−1.752 65	B	1.158 10	3.683 45	1.698 27
B	2.428 39	4.486 04	−1.824 33	N	2.408 78	4.401 62	1.646 05
N	3.653 78	3.741 17	−1.773 78	B	3.682 53	3.750 08	1.611 25
H	4.523 57	4.264 34	−1.830 83	H	4.703 68	4.401 62	1.543 58
H	2.451 67	5.698 20	−1.916 77	H	2.392 76	5.415 40	1.575 88
B	−0.126 01	4.422 78	−1.802 69	N	−0.124 08	4.336 02	1.677 70
H	−0.160 68	5.635 07	−1.897 38	H	−0.152 89	5.348 27	1.588 69
N	−1.386 64	3.685 21	−1.753 27	B	−1.366 96	3.611 50	1.698 95
B	−1.337 53	2.230 22	−1.528 60	N	−1.325 19	2.146 54	1.753 41
N	−2.568 70	1.387 17	−1.550 95	B	−2.571 45	1.387 74	1.726 49
B	−3.844 21	2.075 61	−1.701 37	N	−3.855 37	2.081 07	1.673 46
N	−5.065 93	1.293 77	−1.774 16	B	−5.090 41	1.313 91	1.614 11
B	−5.098 16	−0.139 91	−1.825 42	N	−5.017 71	−0.114 98	1.648 52
H	−6.159 44	−0.725 94	−1.918 35	H	−5.887 72	−0.635 75	1.578 82
H	−5.953 97	1.785 37	−1.830 86	H	−6.165 28	1.872 50	1.547 74
N	−3.860 81	3.525 90	−1.774 74	B	−3.891 17	3.534 53	1.613 41
H	−4.759 08	3.998 48	−1.832 06	H	−4.947 77	4.126 91	1.546 41
B	−2.679 91	4.339 62	−1.825 30	N	−2.656 51	4.257 44	1.647 42
H	−2.772 44	5.548 41	−1.918 46	H	−2.698 13	5.270 53	1.577 48

Table TS4: B97-D3/SVP optimized geometry of the Al_N(AB)

At.	X	Y	Z	At.	X	Y	Z
B	3.367 99	−4.001 18	−1.361 83	N	0.723 67	−5.158 25	1.854 92
N	3.518 78	−2.570 77	−1.599 04	B	−0.464 49	−4.334 18	1.816 98
B	2.360 62	−1.726 51	−1.941 88	N	−1.800 97	−4.886 12	1.855 11
N	1.033 73	−2.393 35	−2.029 09	B	−2.976 33	−4.065 74	1.842 00
B	0.873 48	−3.776 99	−1.573 97	N	−2.822 55	−2.620 08	1.801 47
N	2.056 54	−4.566 88	−1.326 26	B	−1.484 41	−2.031 52	1.744 06
H	1.964 61	−5.545 71	−1.070 48	N	−1.328 88	−0.585 89	1.701 07
N	−0.463 32	−4.310 44	−1.438 80	B	0.003 01	0.002 87	1.687 57
H	−0.565 65	−5.269 03	−1.117 86	N	0.159 07	1.450 69	1.699 42
B	−1.657 21	−3.506 31	−1.572 18	B	−1.015 07	2.308 31	1.741 59
N	−2.980 16	−4.028 30	−1.322 77	N	−2.344 63	1.718 18	1.751 34
H	−3.096 88	−5.004 46	−1.066 91	B	−2.502 79	0.272 17	1.741 31
B	−4.142 56	−3.198 33	−1.357 47	N	−3.838 81	−0.321 26	1.797 40
H	−5.237 30	−3.677 26	−1.151 72	B	−5.011 58	0.538 11	1.834 42
N	−3.987 97	−1.768 37	−1.595 03	N	−4.827 15	1.959 52	1.846 84
B	−2.677 85	−1.187 92	−1.939 00	B	−3.519 29	2.576 39	1.811 20
N	−1.521 95	−2.120 08	−2.027 51	N	−3.329 03	4.009 73	1.850 03
B	−0.194 02	−1.785 13	−2.559 67	B	−2.030 85	4.617 41	1.838 55
Al	−0.004 09	−0.003 98	−3.593 43	N	−0.855 69	3.761 42	1.799 61
B	1.634 88	0.722 71	−2.562 37	B	0.471 12	4.343 90	1.815 01
N	2.590 16	−0.259 61	−2.032 03	N	1.643 21	3.492 01	1.797 10
B	3.859 46	0.316 59	−1.580 32	B	2.973 89	4.077 91	1.832 86
N	4.973 56	−0.568 15	−1.333 46	N	4.112 72	3.207 55	1.842 17
B	4.835 28	−1.989 80	−1.365 73	B	3.993 01	1.766 52	1.805 38
H	5.797 65	−2.698 45	−1.161 28	N	2.662 29	1.178 34	1.747 97
H	5.878 05	−0.181 15	−1.080 31	B	2.508 65	−0.268 12	1.738 69
N	3.959 14	1.752 72	−1.447 93	N	3.687 48	−1.132 65	1.794 62
H	4.841 10	2.143 52	−1.128 89	B	3.528 58	−2.572 87	1.812 57
B	2.828 22	2.643 53	−1.580 10	N	2.204 74	−3.162 05	1.799 92
N	1.548 87	2.090 31	−2.032 27	B	2.046 86	−4.607 26	1.840 52
B	0.307 96	2.905 60	−1.942 44	H	3.013 03	−5.339 84	1.878 93
N	−1.077 39	2.370 79	−2.030 24	B	1.022 76	−2.301 82	1.743 12
B	−1.451 35	1.052 59	−2.560 57	N	−0.308 58	−2.887 88	1.755 19
N	−2.592 15	0.294 33	−2.029 12	N	1.178 80	−0.856 27	1.699 67
B	−3.710 60	1.125 74	−1.576 41	H	4.506 44	−3.287 16	1.844 63
N	−4.986 50	0.496 54	−1.329 17	B	5.016 47	−0.542 94	1.830 09
B	−5.151 80	−0.922 27	−1.361 01	N	5.139 33	0.885 12	1.840 86
H	−6.242 07	−1.411 55	−1.155 90	H	6.072 30	1.282 49	1.908 31
H	−5.788 77	1.065 90	−1.075 84	H	6.008 13	−1.240 65	1.867 52
N	−3.504 04	2.550 28	−1.443 15	B	1.489 13	2.038 34	1.739 49
H	−4.283 16	3.118 59	−1.123 22	H	5.034 60	3.630 01	1.909 67
B	−2.210 37	3.181 74	−1.576 31	H	3.125 35	5.280 91	1.871 45
N	−2.000 66	4.588 52	−1.327 69	H	0.601 00	5.547 83	1.848 84
B	−0.700 55	5.179 78	−1.362 15	H	−1.922 38	5.825 00	1.877 84
N	0.460 42	4.330 58	−1.599 15	H	−4.139 57	4.618 90	1.919 42
B	1.774 95	4.915 49	−1.364 77	H	−5.653 91	2.546 57	1.915 69
N	2.921 03	4.063 01	−1.332 50	H	−6.129 13	0.067 71	1.872 05
H	3.815 13	4.472 97	−1.078 59	B	−3.990 42	−1.762 29	1.814 66
H	1.896 26	6.104 32	−1.159 55	H	−5.098 00	−2.251 87	1.846 72
H	−0.567 70	6.367 29	−1.156 47	H	−4.076 38	−4.575 54	1.881 80
H	−2.787 58	5.177 81	−1.071 84	H	−1.923 27	−5.892 55	1.926 01
H	4.337 02	−4.700 59	−1.156 99	H	0.628 71	−6.167 65	1.925 41

Table TS5: B97-D3/SVP optimized geometry of the Al_N(AA')

At.	X	Y	Z	At.	X	Y	Z
B	−4.297 39	2.987 50	−1.358 72	N	−4.292 07	2.946 44	1.872 48
H	−5.086 75	3.889 84	−1.166 04	H	−4.972 38	3.701 20	1.868 67
N	−2.887 17	3.272 71	−1.590 36	B	−2.883 89	3.268 57	1.847 41
B	−2.428 02	4.636 57	−1.360 34	N	−2.388 95	4.625 66	1.870 31
H	−3.224 74	5.532 96	−1.170 27	H	−3.053 07	5.394 69	1.866 48
N	−1.025 64	4.907 76	−1.331 29	B	−0.994 91	4.941 05	1.803 58
H	−0.722 40	5.845 20	−1.082 29	H	−0.628 58	6.098 94	1.779 48
B	−0.041 63	3.878 44	−1.571 63	N	−0.027 93	3.854 69	1.778 94
N	1.379 38	4.110 38	−1.437 68	B	1.392 41	4.134 44	1.743 52
H	1.687 90	5.028 11	−1.127 33	H	1.779 11	5.285 21	1.702 05
B	2.371 95	3.067 59	−1.573 87	N	2.355 56	3.053 68	1.778 17
N	3.778 02	3.293 21	−1.334 48	B	3.782 37	3.335 58	1.802 15
H	4.102 90	4.223 35	−1.085 40	H	4.189 62	4.479 73	1.779 39
B	4.731 33	2.229 46	−1.362 67	N	4.703 10	2.242 25	1.866 78
H	5.907 59	2.462 01	−1.171 18	H	5.696 88	2.454 12	1.863 37
N	4.272 90	0.865 47	−1.593 03	B	4.277 95	0.861 61	1.843 00
B	2.865 92	0.581 52	−1.933 29	N	2.850 82	0.573 28	1.783 85
N	1.938 83	1.740 96	−2.021 22	B	1.890 97	1.665 62	1.762 72
B	0.568 37	1.699 99	−2.549 96	N	0.466 05	1.377 87	1.735 51
N	−0.498 51	2.559 86	−2.019 20	B	−0.495 86	2.467 77	1.764 04
B	−1.937 80	2.196 20	−1.930 57	N	−1.920 59	2.176 91	1.786 75
N	−2.478 80	0.813 77	−2.020 08	B	−2.386 69	0.799 55	1.764 91
B	−3.843 90	0.525 08	−1.571 74	N	−3.821 09	0.507 97	1.781 16
N	−4.741 91	1.629 97	−1.330 32	B	−4.778 68	1.602 58	1.806 84
H	−5.709 55	1.446 21	−1.080 02	H	−5.973 18	1.383 22	1.784 32
N	−4.250 71	−0.856 07	−1.436 92	B	−4.275 60	−0.866 42	1.745 65
H	−5.199 65	−1.048 13	−1.126 45	H	−5.465 59	−1.106 78	1.704 60
B	−3.339 65	−1.970 78	−1.573 56	N	−3.323 16	−1.956 60	1.779 34
N	−1.969 43	−1.706 52	−2.021 47	B	−1.888 04	−1.668 50	1.763 51
B	−1.759 40	−0.352 06	−2.551 57	N	−1.425 07	−0.290 56	1.736 03
Al	−0.003 30	0.002 62	−3.584 35	B	0.002 15	−0.002 28	1.728 30
B	1.182 82	−1.340 66	−2.552 45	N	0.965 49	−1.094 04	1.734 73
N	2.461 71	−0.846 79	−2.023 30	B	2.390 36	−0.806 04	1.761 97
B	3.375 60	−1.901 80	−1.576 67	N	3.357 50	−1.904 85	1.776 35
N	4.759 10	−1.564 29	−1.337 22	B	4.781 85	−1.610 68	1.800 71
B	5.224 74	−0.213 89	−1.363 71	N	5.205 84	−0.245 65	1.865 76
H	6.399 06	0.028 12	−1.172 03	H	6.203 92	−0.055 05	1.862 07
H	5.419 62	−2.295 68	−1.089 10	H	5.601 40	−2.506 93	1.777 33
N	2.865 58	−3.248 22	−1.442 10	B	2.889 51	−3.274 75	1.740 99
H	3.506 19	−3.974 64	−1.132 76	H	3.692 64	−4.185 08	1.698 60
B	1.465 92	−3.585 85	−1.576 32	N	1.471 94	−3.568 42	1.775 84
N	0.532 95	−2.547 33	−2.022 24	B	0.502 19	−2.471 94	1.762 02
B	−0.934 59	−2.770 81	−1.933 07	N	−0.923 77	−2.756 90	1.784 35
N	−1.391 63	−4.131 51	−1.593 33	B	−1.387 68	−4.137 01	1.842 37
B	−2.802 16	−4.416 41	−1.363 05	N	−2.810 52	−4.386 85	1.865 86
N	−3.738 79	−3.337 92	−1.334 68	B	−3.780 68	−3.337 15	1.802 33
H	−4.702 06	−3.544 34	−1.085 11	H	−4.966 65	−3.598 66	1.779 60
H	−3.179 53	−5.554 58	−1.171 90	H	−3.144 53	−5.346 50	1.861 68
B	−0.439 19	−5.210 40	−1.364 14	N	−0.404 68	−5.195 59	1.864 08
H	−0.825 60	−6.345 42	−1.172 43	H	−0.718 23	−6.162 11	1.859 82
N	0.958 66	−4.916 66	−1.337 73	B	1.002 55	−4.944 99	1.799 04
H	1.602 04	−5.663 29	−1.090 00	H	1.789 65	−5.869 86	1.775 09

Table TS6: B97-D3/SVP optimized geometry of the Layer(AB)

At.	X	Y	Z	At.	X	Y	Z
B	−3.633 31	3.810 53	−1.626 89	N	−1.046 19	5.100 29	1.684 89
N	−3.699 46	2.358 39	−1.645 85	B	0.192 85	4.354 39	1.665 75
B	−2.462 41	1.571 00	−1.651 13	N	1.492 35	4.989 48	1.680 03
N	−1.169 97	2.239 70	−1.647 30	B	2.717 87	4.247 61	1.652 86
B	−1.106 78	3.696 88	−1.624 85	N	2.657 06	2.793 98	1.641 61
N	−2.347 74	4.440 12	−1.613 30	B	1.357 70	2.120 10	1.634 34
H	−2.317 38	5.454 93	−1.576 15	N	1.294 09	0.666 97	1.628 30
N	0.184 83	4.335 21	−1.609 19	B	0.002 46	−0.005 34	1.625 28
H	0.228 93	5.348 94	−1.560 89	N	−0.060 99	−1.460 04	1.627 31
B	1.416 24	3.587 61	−1.627 98	B	1.165 56	−2.241 71	1.633 56
N	2.716 93	4.220 95	−1.620 42	N	2.454 92	−1.567 65	1.641 29
H	2.774 69	5.234 64	−1.585 08	B	2.520 89	−0.114 30	1.632 81
B	3.943 17	3.482 70	−1.635 83	N	3.818 34	0.563 55	1.640 48
H	5.008 46	4.063 09	−1.628 70	B	5.043 70	−0.220 48	1.650 77
N	3.883 65	2.030 28	−1.654 04	N	4.948 64	−1.650 05	1.673 97
B	2.583 42	1.352 82	−1.656 11	B	3.682 96	−2.350 18	1.661 92
N	1.353 45	2.130 51	−1.649 69	N	3.583 45	−3.793 16	1.680 20
B	0.060 34	1.459 54	−1.653 41	B	2.328 15	−4.483 48	1.659 01
N	−0.002 50	0.005 34	−1.656 99	N	1.099 70	−3.703 93	1.643 41
B	−1.293 41	−0.667 42	−1.655 61	B	−0.188 00	−4.369 81	1.633 28
N	−2.521 02	0.116 97	−1.649 92	N	−1.412 65	−3.594 18	1.638 24
B	−3.814 48	−0.557 19	−1.632 51	B	−2.704 43	−4.263 48	1.646 67
N	−5.013 27	0.252 42	−1.623 38	N	−3.894 82	−3.466 37	1.671 93
B	−4.987 09	1.683 70	−1.631 71	B	−3.868 53	−2.020 29	1.658 73
H	−6.022 50	2.315 90	−1.624 43	N	−2.576 66	−1.347 96	1.640 04
H	−5.919 94	−0.204 77	−1.590 62	B	−2.515 89	0.105 60	1.635 66
N	−3.846 06	−1.997 65	−1.619 99	N	−3.749 10	0.893 82	1.647 61
H	−4.746 07	−2.466 80	−1.577 37	B	−3.682 10	2.341 92	1.643 65
B	−2.647 33	−2.796 72	−1.636 27	N	−2.397 92	3.014 64	1.650 56
N	−1.353 74	−2.122 97	−1.653 89	B	−2.331 84	4.467 97	1.662 40
B	−0.128 43	−2.908 12	−1.658 99	H	−3.343 19	5.137 94	1.669 52
N	1.160 01	−2.231 64	−1.654 52	B	−1.162 36	2.230 11	1.638 22
B	1.225 24	−0.776 21	−1.657 08	N	0.129 37	2.899 51	1.644 59
N	2.516 50	−0.100 74	−1.654 00	N	−1.225 54	0.776 97	1.631 09
B	3.746 75	−0.884 04	−1.637 87	H	−4.703 94	2.993 05	1.643 73
N	5.011 14	−0.181 14	−1.636 14	B	−5.038 48	0.219 81	1.656 62
B	5.108 32	1.246 94	−1.643 43	N	−5.068 23	−1.212 58	1.676 74
H	6.194 29	1.787 69	−1.636 57	H	−5.975 25	−1.669 49	1.708 47
H	5.875 29	−0.714 52	−1.605 20	H	−6.072 98	0.853 49	1.663 14
N	3.653 81	−2.321 54	−1.617 53	B	−1.351 16	−2.131 76	1.631 32
H	4.509 54	−2.866 52	−1.567 17	H	−4.789 15	−3.947 69	1.703 57
B	2.390 75	−3.014 31	−1.633 85	H	−2.779 01	−5.474 38	1.648 49
N	2.288 65	−4.457 24	−1.624 52	H	−0.240 95	−5.580 40	1.629 26
B	1.036 40	−5.150 30	−1.641 25	H	2.296 20	−5.696 28	1.667 71
N	−0.191 89	−4.372 87	−1.658 12	H	4.432 62	−4.350 10	1.715 67
B	−1.482 58	−5.041 61	−1.643 23	H	5.812 63	−2.183 95	1.705 82
N	−2.670 57	−4.243 27	−1.630 30	H	6.129 82	0.320 11	1.653 55
H	−3.564 66	−4.724 57	−1.597 33	B	3.877 64	2.012 06	1.632 94
H	−1.557 43	−6.252 47	−1.637 58	H	4.952 38	2.571 49	1.627 41
H	1.006 66	−6.363 12	−1.635 90	H	3.783 81	4.826 88	1.654 74
H	3.137 32	−5.014 25	−1.585 32	H	1.550 49	6.003 40	1.713 44
H	−4.644 35	4.480 95	−1.616 96	H	−1.015 49	6.115 38	1.719 23

Table TS7: B97-D3/SVP optimized geometry of the C_B(AB)

At.	X	Y	Z	At.	X	Y	Z
N	−4.954 71	1.592 56	1.670 74	B	−5.098 35	−1.289 58	−1.627 47
B	−3.697 34	2.305 99	1.651 51	N	−3.866 67	−2.061 35	−1.647 74
N	−3.609 77	3.749 00	1.670 79	B	−2.572 86	−1.371 81	−1.658 57
B	−2.356 63	4.439 99	1.653 16	N	−2.519 11	0.082 48	−1.659 36
N	−1.122 47	3.666 60	1.643 57	B	−3.756 29	0.854 12	−1.634 54
B	−1.178 80	2.206 58	1.626 07	N	−5.014 15	0.139 37	−1.618 72
N	0.047 70	1.411 01	1.613 77	H	−5.882 65	0.665 12	−1.581 23
C	−0.000 32	0.000 41	1.564 14	N	−3.677 46	2.292 69	−1.619 90
N	1.197 27	−0.746 49	1.613 85	H	−4.538 41	2.829 46	−1.569 61
B	2.499 51	−0.082 13	1.626 71	B	−2.420 41	2.996 61	−1.634 35
N	2.562 47	1.365 93	1.628 24	N	−2.332 21	4.440 66	−1.618 10
B	1.325 43	2.121 34	1.626 56	H	−3.186 54	4.989 10	−1.580 00
N	1.368 57	3.581 83	1.644 06	B	−1.086 17	5.145 15	−1.627 02
B	2.652 48	4.269 46	1.653 91	H	−1.067 49	6.358 16	−1.612 99
N	3.855 68	3.494 80	1.672 11	N	0.148 95	4.378 89	−1.647 80
B	3.844 89	2.049 18	1.653 04	B	0.099 23	2.913 67	−1.658 69
N	5.050 82	1.251 88	1.672 74	N	−1.183 00	2.225 33	−1.659 34
B	5.022 67	−0.178 83	1.654 70	B	−1.233 86	0.769 08	−1.670 58
N	3.735 82	−0.860 89	1.644 29	N	0.000 34	−0.000 42	−1.687 41
B	3.697 65	−2.305 78	1.641 32	B	−0.048 99	−1.453 97	−1.670 22
N	2.416 87	−2.975 78	1.643 74	N	−1.335 60	−2.138 05	−1.658 83
B	2.370 46	−4.431 48	1.653 44	B	−1.384 83	−3.595 28	−1.633 36
N	1.097 98	−5.086 18	1.671 55	N	−2.679 52	−4.240 93	−1.617 21
B	−0.148 58	−4.354 03	1.652 57	B	−3.912 70	−3.514 12	−1.626 59
N	−0.099 14	−2.901 79	1.628 07	H	−4.972 53	−4.104 44	−1.612 65
B	−1.321 75	−2.123 26	1.626 82	H	−2.727 31	−5.255 02	−1.579 04
N	−2.614 35	−2.804 50	1.644 44	N	−0.146 67	−4.331 94	−1.618 56
B	−3.846 59	−2.048 96	1.641 58	H	−0.181 06	−5.345 91	−1.568 02
N	−3.786 44	−0.604 79	1.643 70	B	1.138 59	−3.680 91	−1.633 32
B	−5.023 95	0.163 21	1.653 08	N	1.188 28	−2.223 70	−1.658 79
H	−6.099 36	−0.397 44	1.662 43	B	2.474 63	−1.543 08	−1.658 37
B	−2.500 01	0.088 07	1.626 23	N	2.519 61	−0.088 49	−1.659 06
N	−2.464 35	1.537 05	1.627 23	B	1.283 85	0.683 64	−1.670 42
N	−1.245 93	−0.663 31	1.614 00	N	1.331 85	2.140 00	−1.659 15
H	−4.914 89	−2.618 19	1.645 29	B	2.618 70	2.825 62	−1.634 31
B	−2.667 07	−4.260 00	1.654 42	N	2.628 58	4.272 33	−1.618 32
N	−1.442 04	−4.999 72	1.672 20	B	1.433 17	5.059 67	−1.627 12
H	−1.495 51	−6.013 71	1.707 21	H	1.496 74	6.271 15	−1.613 06
H	−3.732 21	−4.839 92	1.663 95	H	3.518 11	4.761 61	−1.580 35
B	1.173 59	−2.208 20	1.626 46	N	3.825 16	2.038 10	−1.620 06
H	1.082 39	−6.101 47	1.706 53	H	4.720 54	2.515 33	−1.570 48
H	3.393 69	−5.082 53	1.662 83	B	3.806 27	0.597 50	−1.634 27
H	4.724 78	−2.946 32	1.645 01	N	5.012 79	−0.200 90	−1.618 30
H	6.057 46	−0.811 34	1.664 42	B	4.999 88	−1.632 24	−1.626 94
H	5.955 70	1.712 56	1.708 00	N	3.718 70	−2.318 75	−1.647 39
H	4.742 77	3.988 92	1.706 90	B	3.666 13	−3.771 28	−1.626 38
H	2.704 69	5.481 13	1.663 03	N	2.386 52	−4.412 84	−1.617 18
B	0.147 95	4.356 00	1.641 33	H	2.365 51	−5.427 84	−1.579 12
H	0.189 09	5.565 79	1.645 19	H	4.683 49	−4.432 10	−1.612 42
H	−2.326 30	5.652 40	1.662 70	H	6.041 04	−2.254 93	−1.613 01
H	−4.461 17	4.302 34	1.705 64	H	5.914 94	0.264 78	−1.580 71
H	−5.826 17	2.113 73	1.705 52	H	−6.179 33	−1.840 22	−1.613 82

Table TS8: B97-D3/SVP optimized geometry of the C_B-C_N(AB)

At.	X	Y	Z	At.	X	Y	Z
B	−5.161 61	−1.110 40	−1.626 11	N	−4.892 02	1.769 58	1.651 50
N	−3.959 72	−1.929 55	−1.610 01	B	−3.619 77	2.443 47	1.610 96
B	−2.631 47	−1.282 29	−1.593 61	N	−3.470 64	3.875 42	1.651 53
N	−2.558 23	0.177 58	−1.570 32	B	−2.191 99	4.515 41	1.622 92
B	−3.752 16	0.993 47	−1.602 94	N	−0.989 42	3.687 31	1.599 90
N	−5.039 69	0.319 24	−1.610 71	B	−1.112 08	2.243 24	1.542 39
H	−5.896 27	0.865 92	−1.618 58	N	0.096 97	1.377 21	1.528 22
N	−3.612 74	2.438 27	−1.594 05	C	0.000 26	0.000 10	1.453 47
H	−4.455 15	3.006 89	−1.603 27	N	1.144 54	−0.772 19	1.528 36
B	−2.324 89	3.107 90	−1.603 14	B	2.499 08	−0.158 11	1.542 42
N	−2.181 07	4.554 14	−1.611 05	N	2.610 09	1.272 06	1.570 10
H	−3.008 44	5.144 12	−1.619 00	B	1.415 21	2.065 78	1.542 23
B	−0.900 75	5.201 86	−1.626 45	N	1.495 63	3.512 82	1.599 64
H	−0.836 30	6.415 38	−1.643 29	B	2.802 16	4.164 69	1.622 41
N	0.308 48	4.393 56	−1.610 36	N	3.978 79	3.352 23	1.651 30
B	0.204 92	2.919 65	−1.593 93	B	3.926 30	1.913 50	1.610 91
N	−1.121 66	2.305 78	−1.570 52	N	5.091 85	1.068 38	1.651 56
B	−1.230 91	0.830 59	−1.558 94	B	5.006 81	−0.358 96	1.622 90
C	−0.000 26	−0.000 11	−1.491 22	N	3.688 38	−0.986 37	1.600 00
B	−0.104 36	−1.481 25	−1.558 89	B	3.599 05	−2.428 81	1.599 83
N	−1.436 56	−2.124 24	−1.570 23	N	2.294 75	−3.051 22	1.599 92
B	−1.529 65	−3.567 31	−1.602 61	B	2.206 01	−4.508 63	1.622 54
N	−2.854 06	−4.165 84	−1.610 17	N	0.914 07	−5.121 40	1.651 18
B	−4.055 14	−3.380 90	−1.625 85	B	−0.305 68	−4.356 58	1.610 88
H	−5.138 31	−3.931 84	−1.642 65	N	−0.203 10	−2.896 00	1.570 37
H	−2.951 36	−5.177 35	−1.617 91	B	−1.386 19	−2.084 78	1.542 70
N	−0.305 82	−4.347 79	−1.593 61	N	−2.698 13	−2.700 58	1.600 32
H	−0.377 07	−5.361 65	−1.602 64	B	−3.902 67	−1.902 00	1.600 20
B	1.015 14	−3.746 13	−1.602 85	N	−3.789 54	−0.461 21	1.600 24
N	1.124 81	−2.304 22	−1.570 38	B	−5.007 34	0.344 34	1.622 92
B	2.425 73	−1.637 65	−1.593 95	H	−6.095 19	−0.186 07	1.651 95
N	2.557 42	−0.181 88	−1.570 58	B	−2.496 14	0.192 66	1.542 57
B	1.334 48	0.650 33	−1.559 01	N	−2.406 16	1.624 31	1.570 26
N	1.432 61	2.126 30	−1.570 55	N	−1.240 70	−0.604 70	1.528 49
B	2.736 20	2.752 25	−1.603 16	H	−4.988 27	−2.431 10	1.634 00
N	2.796 03	4.204 40	−1.610 82	B	−2.813 96	−4.156 09	1.622 90
B	1.618 86	5.024 80	−1.626 29	N	−1.620 34	−4.943 41	1.651 40
H	1.724 75	6.235 41	−1.643 10	H	−1.715 65	−5.954 31	1.688 00
H	3.697 74	4.672 91	−1.618 64	H	−3.901 99	−4.686 14	1.651 89
N	3.917 74	1.909 10	−1.594 38	B	1.081 75	−2.258 09	1.542 49
H	4.831 38	2.354 35	−1.603 65	H	0.867 15	−6.135 69	1.687 78
B	3.853 73	0.458 99	−1.603 42	H	3.209 29	−5.185 54	1.651 44
N	5.034 29	−0.388 72	−1.611 53	H	4.600 07	−3.104 43	1.633 60
B	4.955 02	−1.821 35	−1.627 08	H	6.009 85	−1.036 22	1.651 97
N	3.650 38	−2.464 34	−1.610 73	H	6.014 95	1.491 30	1.688 30
B	3.541 87	−3.914 77	−1.626 72	H	4.880 65	3.818 74	1.688 02
N	2.242 81	−4.524 01	−1.610 78	H	2.886 72	5.372 01	1.651 36
H	2.197 67	−5.539 18	−1.618 62	B	0.304 45	4.331 17	1.599 55
H	4.537 34	−4.611 77	−1.643 90	H	0.389 04	5.535 88	1.633 36
H	5.973 70	−2.483 97	−1.644 28	H	−2.106 95	5.722 69	1.652 27
H	5.958 92	0.032 78	−1.619 63	H	−4.298 44	4.463 40	1.688 33
H	−6.262 95	−1.624 03	−1.642 87	H	−5.746 95	2.317 37	1.688 13

Table TS9: B97-D3/SVP optimized geometry of the C_B-C_N(AA')

At.	X	Y	Z	At.	X	Y	Z
B	4.349 84	2.991 69	1.616 70	N	4.298 23	2.930 44	−1.668 36
H	5.173 68	3.888 21	1.600 17	H	4.989 11	3.674 96	−1.635 00
N	2.928 47	3.294 65	1.623 85	B	2.900 40	3.268 95	−1.654 87
B	2.459 38	4.670 18	1.620 54	N	2.398 97	4.616 93	−1.664 68
H	3.252 13	5.594 32	1.604 48	H	3.056 61	5.390 94	−1.631 09
N	1.050 62	4.940 63	1.618 83	B	1.004 36	4.917 98	−1.594 68
H	0.752 68	5.912 23	1.609 15	H	0.620 38	6.066 06	−1.564 55
B	0.055 35	3.879 78	1.622 73	N	0.042 41	3.817 02	−1.598 42
N	−1.374 36	4.137 14	1.596 76	B	−1.370 72	4.116 86	−1.545 40
H	−1.695 27	5.101 80	1.586 92	H	−1.752 34	5.263 82	−1.512 65
B	−2.365 22	3.075 02	1.622 69	N	−2.322 69	3.030 32	−1.597 70
N	−3.797 70	3.328 53	1.619 11	B	−3.752 47	3.335 90	−1.592 62
H	−4.141 16	4.284 99	1.608 99	H	−4.132 63	4.485 27	−1.563 24
B	−4.763 91	2.268 34	1.621 18	N	−4.689 17	2.259 69	−1.660 31
H	−5.952 25	2.533 61	1.604 95	H	−5.679 31	2.485 73	−1.625 48
N	−4.315 73	0.885 88	1.625 18	B	−4.283 41	0.879 94	−1.650 47
B	−2.864 96	0.588 08	1.627 65	N	−2.846 01	0.584 79	−1.640 26
N	−1.918 61	1.701 42	1.609 81	B	−1.887 53	1.649 04	−1.594 39
B	−0.465 57	1.404 96	1.607 97	N	−0.436 22	1.307 09	−1.600 40
N	0.520 39	2.512 29	1.609 84	B	0.521 04	2.450 27	−1.595 61
B	1.945 17	2.187 35	1.627 59	N	1.926 07	2.171 90	−1.642 57
N	2.436 27	0.811 10	1.608 46	B	2.368 74	0.809 60	−1.599 61
B	3.849 31	0.511 14	1.618 46	N	3.782 57	0.495 85	−1.605 05
N	4.785 07	1.624 87	1.612 04	B	4.762 14	1.581 14	−1.601 29
H	5.785 08	1.443 96	1.599 92	H	5.947 63	1.335 59	−1.573 58
N	4.273 93	−0.877 97	1.592 07	B	4.247 54	−0.871 92	−1.553 56
H	5.269 75	−1.082 55	1.580 40	H	5.431 69	−1.115 05	−1.523 02
B	3.336 26	−1.987 38	1.620 49	N	3.281 14	−1.945 72	−1.604 10
N	1.919 33	−1.706 32	1.609 76	B	1.858 04	−1.676 84	−1.599 19
B	1.453 11	−0.298 81	1.607 87	N	1.346 74	−0.276 15	−1.604 04
C	0.001 89	−0.000 78	1.559 89	C	−0.001 79	0.000 63	−1.557 87
B	−0.981 76	−1.108 20	1.607 50	N	−0.916 06	−1.028 83	−1.602 22
N	−2.433 98	−0.808 15	1.608 61	B	−2.384 78	−0.771 26	−1.597 00
B	−3.385 86	−1.894 51	1.619 44	N	−3.329 13	−1.869 18	−1.603 09
N	−4.802 38	−1.563 15	1.615 03	B	−4.763 62	−1.586 63	−1.597 74
B	−5.272 51	−0.207 94	1.619 06	N	−5.200 15	−0.228 18	−1.662 09
H	−6.469 22	0.016 55	1.602 70	H	−6.199 21	−0.045 57	−1.627 17
H	−5.494 87	−2.306 94	1.603 42	H	−5.565 96	−2.493 28	−1.570 56
N	−2.893 92	−3.261 23	1.592 77	B	−2.882 38	−3.242 96	−1.552 36
H	−3.568 96	−4.021 39	1.581 78	H	−3.685 07	−4.146 93	−1.522 63
B	−1.478 50	−3.588 10	1.620 58	N	−1.465 40	−3.524 13	−1.602 66
N	−0.512 09	−2.514 64	1.609 55	B	−0.486 69	−2.456 68	−1.597 65
B	0.925 47	−2.777 54	1.628 82	N	0.914 36	−2.754 40	−1.642 37
N	1.392 74	−4.182 74	1.626 34	B	1.377 40	−4.146 72	−1.651 72
B	2.818 59	−4.464 38	1.620 77	N	2.795 43	−4.386 56	−1.662 57
N	3.757 33	−3.379 75	1.616 96	B	3.753 61	−3.329 27	−1.597 73
H	4.747 69	−3.607 73	1.605 88	H	4.939 92	−3.570 76	−1.569 73
H	3.222 51	−5.613 01	1.604 80	H	3.136 92	−5.343 01	−1.626 94
B	0.419 44	−5.262 01	1.620 57	N	0.385 38	−5.187 95	−1.660 94
H	0.783 87	−6.423 77	1.603 75	H	0.684 84	−6.158 36	−1.625 06
N	−0.981 94	−4.955 38	1.616 61	B	−1.015 12	−4.915 03	−1.595 80
H	−1.638 63	−5.730 94	1.604 95	H	−1.820 49	−5.818 90	−1.566 73

Table TS10: B97-D3/SVP optimized geometry of the C_B(AA')

At.	X	Y	Z	At.	X	Y	Z
N	−4.409 09	−2.769 72	1.652 81	B	−4.460 90	−2.776 97	−1.609 77
H	−5.369 51	−3.099 14	1.616 43	H	−5.608 84	−3.173 46	−1.574 72
B	−4.147 70	−1.349 13	1.658 15	N	−4.166 05	−1.354 71	−1.637 21
N	−5.195 03	−0.354 38	1.652 36	B	−5.240 97	−0.377 66	−1.609 53
H	−6.165 41	−0.653 17	1.615 92	H	−6.402 66	−0.731 88	−1.574 64
B	−4.918 79	1.047 67	1.617 69	N	−4.911 22	1.014 90	−1.626 31
H	−5.827 03	1.851 81	1.582 99	H	−5.673 17	1.685 88	−1.585 20
N	−3.533 04	1.497 89	1.636 60	B	−3.547 24	1.497 96	−1.642 47
B	−3.241 82	2.912 72	1.604 23	N	−3.218 96	2.900 74	−1.631 66
H	−4.142 60	3.723 04	1.563 91	H	−3.973 41	3.579 75	−1.581 61
N	−1.865 57	3.351 50	1.637 45	B	−1.858 09	3.374 14	−1.641 62
B	−1.563 82	4.777 05	1.621 02	N	−1.520 62	4.781 08	−1.624 63
H	−2.459 10	5.595 57	1.586 37	H	−2.267 86	5.468 41	−1.583 74
N	−0.198 66	5.199 47	1.657 69	B	−0.170 25	5.255 10	−1.607 87
H	−0.003 50	6.195 98	1.623 90	H	0.060 11	6.447 52	−1.572 59
B	0.900 83	4.262 76	1.662 48	N	0.914 26	4.288 65	−1.635 22
N	0.598 94	2.839 73	1.661 35	B	0.609 44	2.852 88	−1.652 49
B	−0.774 85	2.378 92	1.650 96	N	−0.773 13	2.399 19	−1.657 37
N	−1.052 29	0.943 07	1.648 44	B	−1.078 17	0.973 80	−1.658 00
B	−2.450 90	0.515 76	1.650 60	N	−2.463 85	0.521 29	−1.658 42
N	−2.764 39	−0.898 80	1.659 90	B	−2.770 11	−0.901 02	−1.654 40
B	−1.678 45	−1.857 98	1.650 43	N	−1.685 88	−1.871 49	−1.658 94
N	−1.975 27	−3.288 88	1.637 58	B	−1.987 93	−3.298 42	−1.644 00
B	−3.360 55	−3.740 63	1.618 79	N	−3.375 23	−3.709 45	−1.627 39
H	−3.621 36	−4.925 31	1.583 79	H	−3.597 09	−4.700 20	−1.586 78
B	−0.907 11	−4.261 33	1.605 55	N	−0.897 52	−4.240 20	−1.634 17
H	−1.158 35	−5.446 65	1.565 37	H	−1.108 63	−5.233 09	−1.585 16
N	0.463 70	−3.806 04	1.638 99	B	0.481 60	−3.823 16	−1.642 67
B	0.773 12	−2.377 86	1.651 42	N	0.785 99	−2.396 70	−1.657 32
N	−0.296 25	−1.380 31	1.648 28	B	−0.298 85	−1.422 99	−1.658 55
C	−0.003 45	−0.000 24	1.625 68	N	0.003 75	0.000 40	−1.659 07
N	1.338 15	0.436 15	1.648 34	B	1.387 69	0.450 23	−1.656 28
B	1.667 39	1.860 96	1.651 63	N	1.688 38	1.876 53	−1.656 25
N	3.059 02	2.307 12	1.640 29	B	3.075 88	2.326 48	−1.639 78
B	3.362 10	3.732 32	1.624 24	N	3.339 11	3.749 29	−1.623 80
N	2.285 93	4.672 50	1.659 95	B	2.297 85	4.731 11	−1.607 81
H	2.512 35	5.662 39	1.626 79	H	2.571 80	5.914 28	−1.573 26
H	4.512 72	4.116 70	1.591 46	H	4.301 14	4.073 91	−1.583 43
B	4.138 77	1.347 46	1.608 08	N	4.126 68	1.340 67	−1.627 93
H	5.291 07	1.722 24	1.569 92	H	5.091 97	1.654 51	−1.577 80
N	3.830 50	−0.063 74	1.640 37	B	3.856 42	−0.074 59	−1.637 66
B	2.442 84	−0.522 07	1.651 83	N	2.469 63	−0.526 65	−1.654 81
N	2.154 94	−1.942 02	1.661 99	B	2.171 08	−1.950 73	−1.650 80
B	3.236 52	−2.914 92	1.663 49	N	3.261 89	−2.932 81	−1.632 09
N	2.898 88	−4.319 28	1.659 59	B	2.952 84	−4.352 22	−1.605 98
B	1.546 56	−4.781 13	1.623 23	N	1.581 90	−4.762 78	−1.625 22
H	1.304 32	−5.969 81	1.589 38	H	1.381 59	−5.758 17	−1.585 53
H	3.643 09	−5.010 16	1.626 39	H	3.840 19	−5.181 36	−1.570 38
N	4.597 57	−2.431 15	1.660 05	B	4.641 12	−2.477 04	−1.602 66
H	5.363 11	−3.098 31	1.626 78	H	5.558 44	−3.272 92	−1.567 34
B	4.914 35	−1.037 71	1.624 40	N	4.905 96	−1.070 61	−1.619 13
H	6.070 91	−0.671 60	1.591 56	H	5.874 89	−0.767 43	−1.577 90

Table TS11: B97-D3/SVP optimized geometry of the C_BC_N(AB)

At.	X	Y	Z	At.	X	Y	Z
B	−6.001 84	2.520 15	−0.865 96	B	6.727 90	1.260 03	0.825 24
N	−4.560 56	2.513 53	−1.075 69	H	7.934 06	1.283 59	0.684 20
B	−3.847 12	1.245 69	−1.167 37	N	5.989 14	2.482 96	0.920 83
N	−2.395 37	1.216 34	−1.357 92	H	6.507 73	3.355 14	0.866 47
B	−1.669 23	2.510 92	−1.454 34	B	4.551 38	2.520 41	1.083 63
N	−0.242 13	2.556 21	−1.639 35	N	3.827 04	1.258 26	1.161 69
B	0.476 86	1.296 65	−1.738 74	B	4.552 39	−0.000 19	1.073 96
N	1.925 05	1.272 71	−1.923 51	N	6.010 33	−0.000 15	0.908 52
B	2.649 96	2.531 59	−1.999 08	B	6.727 95	−1.260 29	0.825 04
N	4.084 92	2.488 33	−2.179 47	H	7.934 11	−1.283 77	0.684 00
B	4.815 15	1.260 63	−2.285 30	N	5.989 24	−2.483 26	0.920 43
H	6.018 80	1.279 14	−2.438 05	H	6.507 87	−3.355 41	0.865 96
N	4.094 40	0.000 77	−2.204 97	B	4.551 48	−2.520 79	1.083 23
B	2.636 93	0.000 63	−2.018 29	N	3.812 18	−3.756 32	1.183 89
N	1.925 29	−1.271 57	−1.923 54	H	4.321 07	−4.635 61	1.155 67
B	2.650 41	−2.530 30	−1.999 29	B	2.381 11	−3.783 24	1.354 00
N	4.085 36	−2.486 79	−2.179 67	N	1.630 38	−5.017 69	1.467 48
B	4.815 38	−1.258 96	−2.285 41	H	2.124 43	−5.905 10	1.435 75
H	6.019 02	−1.277 24	−2.438 22	B	0.209 78	−5.048 61	1.638 88
H	4.612 58	−3.354 40	−2.209 53	H	−0.373 84	−6.109 35	1.723 99
N	1.912 19	−3.772 01	−1.889 72	N	−0.513 92	−3.788 97	1.723 35
H	2.428 78	−4.646 65	−1.909 13	B	0.215 60	−2.522 07	1.614 96
B	0.483 77	−3.816 62	−1.715 35	N	1.656 35	−2.521 02	1.430 22
N	−0.282 77	−5.039 04	−1.605 90	B	2.380 57	−1.259 69	1.330 66
H	0.192 12	−5.936 90	−1.630 71	N	3.827 10	−1.258 67	1.161 54
B	−1.701 35	−5.039 60	−1.430 74	N	1.657 17	−0.000 27	1.418 47
H	−2.306 70	−6.086 08	−1.342 64	B	2.380 51	1.259 19	1.330 75
N	−2.415 51	−3.765 71	−1.357 31	N	1.656 24	2.520 48	1.430 46
B	−1.668 77	−2.510 46	−1.454 53	B	2.380 97	3.782 75	1.354 61
N	−0.241 64	−2.555 47	−1.639 41	N	3.812 04	3.755 90	1.184 52
B	0.477 10	−1.295 77	−1.738 70	H	4.320 91	4.635 21	1.156 54
C	−0.309 66	0.000 37	−1.637 07	N	1.630 20	5.017 14	1.468 41
C	−1.681 01	0.000 24	−1.454 35	H	2.124 23	5.904 58	1.436 95
N	−2.395 12	−1.216 01	−1.358 00	B	0.209 61	5.047 97	1.639 85
B	−3.846 89	−1.245 65	−1.167 49	N	−0.514 06	3.788 29	1.723 94
N	−4.560 11	−2.513 63	−1.076 03	B	0.215 49	2.521 44	1.615 18
B	−3.846 19	−3.771 95	−1.168 70	N	−0.507 55	1.262 04	1.703 02
H	−4.450 82	−4.817 30	−1.087 97	B	0.214 13	−0.000 33	1.594 92
B	−6.001 38	−2.520 53	−0.866 24	N	−0.507 49	−1.262 72	1.703 10
H	−6.610 05	−3.565 27	−0.780 14	B	−1.946 30	−1.262 04	1.892 89
N	−6.693 20	−1.271 00	−0.764 31	N	−2.672 50	−2.530 08	1.995 62
B	−6.015 74	−0.000 19	−0.861 92	B	−1.950 26	−3.784 73	1.905 73
N	−4.572 64	−0.000 05	−1.069 60	H	−2.553 01	−4.833 19	1.984 74
N	−6.693 45	1.270 48	−0.764 27	B	−4.116 28	−2.526 71	2.173 38
H	−7.701 67	1.292 44	−0.637 22	H	−4.737 69	−3.565 41	2.246 08
H	−7.701 42	−1.293 17	−0.637 23	N	−4.796 95	−1.269 85	2.256 18
H	4.612 01	3.356 02	−2.209 20	B	−4.111 32	−0.000 44	2.170 45
N	1.911 51	3.773 14	−1.889 41	N	−2.667 34	−0.000 40	1.983 59
B	0.483 07	3.817 48	−1.715 02	B	−1.946 35	1.261 28	1.892 99
N	−0.283 66	5.039 76	−1.605 34	N	−2.672 59	2.529 28	1.996 02
H	0.191 08	5.937 71	−1.630 00	B	−1.950 38	3.783 96	1.906 42
B	−1.702 24	5.040 05	−1.430 08	H	−2.553 14	4.832 39	1.985 82
N	−2.416 19	3.766 03	−1.356 85	B	−4.116 37	2.525 83	2.173 78
B	−3.846 86	3.771 99	−1.168 14	N	−4.797 01	1.268 92	2.256 30
H	−4.451 64	4.817 22	−1.087 10	H	−5.808 13	1.280 90	2.353 57
H	−2.307 75	6.086 41	−1.341 72	H	−4.737 81	3.564 50	2.246 73
H	2.427 96	4.647 88	−1.908 62	H	−5.808 09	−1.281 87	2.353 31
H	−6.610 70	3.564 76	−0.779 72	H	−0.374 03	6.108 68	1.725 34

Table TS12: B97-D3/SVP optimized geometry of the C_BC_N(AA')

At.	X	Y	Z	At.	X	Y	Z
N	5.103 75	−2.490 03	−1.617 33	B	−5.840 73	−1.261 75	1.587 70
B	3.657 48	−2.532 17	−1.648 11	H	−7.054 48	−1.283 89	1.539 99
N	2.928 96	−1.272 70	−1.677 15	N	−5.096 64	−2.483 88	1.610 97
B	1.468 25	−1.296 62	−1.686 09	H	−5.613 45	−3.357 71	1.567 09
N	0.742 68	−2.556 89	−1.678 67	B	−3.650 18	−2.521 88	1.642 23
B	−0.696 26	−2.510 88	−1.663 46	N	−2.922 56	−1.257 65	1.664 43
N	−1.429 53	−1.216 52	−1.671 97	B	−3.653 57	0.000 10	1.654 85
B	−2.893 95	−1.245 40	−1.669 83	N	−5.120 88	0.000 09	1.621 46
N	−3.613 91	−2.513 72	−1.648 04	B	−5.840 75	1.261 93	1.587 70
B	−5.070 86	−2.517 78	−1.637 50	H	−7.054 49	1.284 06	1.540 12
N	−5.769 01	−1.270 27	−1.685 82	N	−5.096 66	2.484 07	1.610 94
H	−6.784 52	−1.288 45	−1.655 34	H	−5.613 49	3.357 89	1.567 16
B	−5.085 50	−0.000 11	−1.689 96	B	−3.650 21	2.522 08	1.642 21
N	−3.626 35	−0.000 11	−1.689 34	N	−2.903 78	3.754 07	1.637 11
B	−2.893 95	1.245 17	−1.669 88	H	−3.409 98	4.633 80	1.585 07
N	−3.613 89	2.513 50	−1.648 14	B	−1.462 78	3.782 49	1.655 83
B	−5.070 85	2.517 56	−1.637 71	N	−0.705 36	5.014 66	1.641 86
N	−5.769 00	1.270 06	−1.685 96	H	−1.201 39	5.900 47	1.599 47
H	−6.784 51	1.288 25	−1.655 66	B	0.725 06	5.045 33	1.617 18
H	−5.684 88	3.563 06	−1.600 01	H	1.314 48	6.106 27	1.578 64
B	−2.891 99	3.768 10	−1.601 75	N	1.455 44	3.787 71	1.645 05
H	−3.502 47	4.814 44	−1.551 61	B	0.720 44	2.520 44	1.669 04
N	−1.449 77	3.766 65	−1.633 82	N	−0.734 25	2.519 20	1.675 58
B	−0.729 60	5.037 62	−1.600 48	B	−1.464 30	1.258 91	1.676 58
H	−1.342 20	6.084 73	−1.555 80	N	−2.922 57	1.257 86	1.664 41
N	0.699 21	5.040 42	−1.628 96	N	−0.736 91	0.000 10	1.683 85
H	1.175 65	5.936 73	−1.580 86	B	−1.464 29	−1.258 71	1.676 58
B	1.472 61	3.817 57	−1.651 93	N	−0.734 24	−2.519 00	1.675 59
N	0.742 67	2.556 67	−1.678 91	B	−1.462 74	−3.782 31	1.655 92
B	−0.696 25	2.510 65	−1.663 55	N	−2.903 74	−3.753 87	1.637 14
N	−1.429 53	1.216 31	−1.672 02	H	−3.409 95	−4.633 59	1.585 12
C	−0.708 07	−0.000 11	−1.681 16	N	−0.705 33	−5.014 48	1.642 18
C	0.674 90	−0.000 11	−1.691 09	H	−1.201 36	−5.900 30	1.600 07
B	1.468 25	1.296 39	−1.686 20	B	0.725 09	−5.045 16	1.618 03
N	2.928 95	1.272 51	−1.677 18	N	1.455 45	−3.787 51	1.645 62
B	3.657 46	2.531 98	−1.647 78	B	0.720 45	−2.520 22	1.669 20
N	2.911 80	3.773 93	−1.636 46	N	1.447 59	−1.261 44	1.682 18
H	3.426 61	4.648 39	−1.576 44	B	0.719 25	0.000 11	1.676 23
N	5.103 72	2.489 84	−1.616 91	N	1.447 58	1.261 67	1.682 10
H	5.627 79	3.358 89	−1.565 41	B	2.901 72	1.260 99	1.673 51
B	5.839 19	1.260 81	−1.594 62	N	3.633 22	2.529 91	1.652 12
N	5.116 81	−0.000 08	−1.632 47	B	2.904 42	3.780 79	1.612 56
B	3.646 45	−0.000 09	−1.666 85	H	3.510 57	4.831 37	1.565 20
B	5.839 19	−1.260 97	−1.594 86	B	5.088 56	2.524 28	1.632 24
H	7.053 48	−1.278 81	−1.545 69	H	5.713 04	3.564 77	1.592 29
H	7.053 48	1.278 72	−1.545 43	N	5.774 99	1.269 50	1.672 61
H	−5.684 90	−3.563 27	−1.599 68	B	5.084 05	0.000 12	1.680 09
B	−2.892 01	−3.768 33	−1.601 76	N	3.626 83	0.000 12	1.687 36
N	−1.449 78	−3.766 84	−1.633 88	B	2.901 72	−1.260 74	1.673 68
B	−0.729 54	−5.037 79	−1.600 56	N	3.633 22	−2.529 68	1.652 66
H	−1.342 22	−6.084 94	−1.555 98	B	2.904 45	−3.780 59	1.613 66
N	0.699 27	−5.040 61	−1.629 08	H	3.510 58	−4.831 24	1.567 39
B	1.472 65	−3.817 77	−1.652 10	B	5.088 55	−2.524 07	1.633 10
N	2.911 83	−3.774 14	−1.637 13	N	5.774 99	−1.269 27	1.672 91
H	3.426 68	−4.648 61	−1.577 72	H	6.790 39	−1.279 32	1.634 78
H	1.175 31	−5.937 10	−1.581 14	H	5.713 04	−3.564 59	1.593 92
H	−3.502 49	−4.814 66	−1.551 69	H	6.790 37	1.279 55	1.633 92
H	5.627 95	−3.359 04	−1.566 53	H	1.314 55	−6.106 13	1.580 38

Table TS13: B97-D3/SVP optimized geometry of the C_N(AB)

At.	X	Y	Z	At.	X	Y	Z
B	−2.633 02	−4.569 15	−1.641 76	N	−4.601 12	−2.448 73	1.672 27
N	−1.217 46	−4.219 67	−1.655 38	B	−4.235 67	−1.049 36	1.656 48
B	−0.811 35	−2.811 68	−1.655 58	N	−5.211 99	0.017 66	1.672 33
N	−1.851 53	−1.773 87	−1.650 82	B	−4.846 63	1.403 01	1.650 28
B	−3.265 31	−2.121 66	−1.637 50	N	−3.435 15	1.755 27	1.640 14
N	−3.616 29	−3.525 89	−1.631 06	B	−2.421 49	0.699 24	1.630 30
H	−4.593 94	−3.801 02	−1.600 11	N	−1.009 77	1.048 82	1.626 11
N	−4.237 89	−1.049 23	−1.624 48	B	0.000 19	−0.000 27	1.619 80
H	−5.223 76	−1.293 42	−1.583 46	N	1.413 75	0.349 80	1.625 75
B	−3.878 23	0.353 17	−1.637 03	B	1.816 85	1.747 16	1.630 20
N	−4.844 03	1.431 25	−1.630 14	N	0.806 01	2.793 30	1.637 74
H	−5.837 07	1.218 25	−1.599 13	B	−0.606 74	2.446 19	1.630 58
B	−4.461 32	2.812 84	−1.640 66	N	−1.623 49	3.499 25	1.640 52
H	−5.311 92	3.678 09	−1.634 55	B	−1.217 79	4.896 29	1.650 77
N	−3.046 26	3.164 37	−1.654 59	N	0.180 46	5.208 69	1.672 65
B	−2.029 96	2.108 69	−1.655 02	B	1.209 61	4.192 52	1.656 82
N	−2.465 55	0.705 40	−1.650 42	N	2.621 86	4.504 52	1.672 44
B	−1.480 76	−0.366 36	−1.648 17	B	3.638 93	3.495 45	1.650 23
C	−0.000 21	0.000 27	−1.638 60	N	3.238 26	2.096 96	1.639 79
B	1.057 62	−1.098 58	−1.648 23	B	4.240 83	1.049 97	1.633 46
N	0.621 81	−2.487 32	−1.650 79	N	3.842 81	−0.343 92	1.639 55
B	1.633 23	−3.534 57	−1.637 33	B	4.849 87	−1.393 75	1.649 91
N	1.182 43	−4.909 98	−1.630 53	N	4.421 31	−2.760 84	1.672 33
B	−0.205 42	−5.269 32	−1.641 30	B	3.026 69	−3.143 98	1.656 61
H	−0.529 42	−6.438 60	−1.635 18	N	2.016 70	−2.094 90	1.637 18
H	1.863 38	−5.663 51	−1.599 32	B	0.605 23	−2.447 21	1.630 08
N	3.027 58	−3.144 87	−1.624 60	N	0.197 52	−3.853 08	1.640 00
H	3.731 97	−3.876 59	−1.583 57	B	−1.210 51	−4.197 81	1.633 82
B	3.470 04	−1.766 36	−1.637 52	N	−2.218 69	−3.156 20	1.639 78
N	2.461 94	−0.715 91	−1.650 80	B	−3.631 43	−3.503 40	1.650 09
B	2.840 60	0.703 85	−1.655 62	H	−3.988 44	−4.662 91	1.655 77
N	1.843 10	1.782 75	−1.650 57	B	−1.815 09	−1.749 13	1.630 07
B	0.422 50	1.465 81	−1.647 94	N	−2.822 09	−0.699 27	1.637 34
N	−0.611 08	2.490 63	−1.650 22	N	−0.403 39	−1.399 47	1.625 81
B	−0.205 33	3.888 84	−1.636 65	H	−1.546 31	−5.362 12	1.631 36
N	−1.245 94	4.894 91	−1.630 02	B	1.208 37	−4.899 28	1.650 68
B	−2.641 10	4.565 03	−1.640 82	N	2.590 78	−4.522 99	1.672 84
H	−3.473 36	5.447 93	−1.634 86	H	3.285 32	−5.264 12	1.705 07
H	−0.995 38	5.879 16	−1.599 02	H	0.893 08	−6.070 82	1.656 71
N	1.209 74	4.194 92	−1.623 83	B	2.422 42	−0.697 88	1.629 89
H	1.491 11	5.170 82	−1.582 47	H	5.135 49	−3.483 06	1.704 44
B	2.244 42	3.182 25	−1.636 97	H	6.032 54	−1.123 16	1.655 51
N	3.660 99	3.479 57	−1.630 48	H	5.417 05	1.341 30	1.630 74
B	4.666 13	2.457 38	−1.641 84	H	4.811 17	3.808 13	1.656 01
N	4.263 04	1.056 13	−1.655 68	H	2.916 46	5.476 57	1.704 34
B	5.273 48	0.004 96	−1.642 09	H	0.448 84	6.188 31	1.704 47
N	4.861 61	−1.368 21	−1.631 13	H	−2.043 43	5.785 24	1.656 48
H	5.588 69	−2.077 34	−1.600 23	B	−3.029 66	3.147 01	1.634 23
H	6.454 24	0.284 27	−1.636 37	H	−3.870 04	4.020 02	1.631 69
H	5.840 76	2.761 39	−1.636 35	H	−5.703 51	2.261 88	1.655 88
H	3.972 96	4.446 10	−1.599 33	H	−6.201 10	−0.213 26	1.704 26
H	−2.981 50	−5.731 38	−1.635 88	H	−5.583 67	−2.706 14	1.704 21

Table TS14: B97-D3/SVP optimized geometry of the C_N(AA')

At.	X	Y	Z	At.	X	Y	Z
B	5.199 04	−0.886 56	−1.603 60	N	5.138 77	−0.855 58	1.661 30
H	6.317 12	−1.360 46	−1.563 25	H	6.074 26	−1.251 31	1.631 06
N	4.029 89	−1.756 02	−1.633 40	B	3.998 89	−1.743 71	1.665 16
B	4.188 55	−3.204 34	−1.603 87	N	4.124 30	−3.183 24	1.660 43
H	5.296 76	−3.700 97	−1.564 10	H	5.051 19	−3.598 67	1.630 08
N	3.028 23	−4.045 63	−1.625 21	B	2.993 41	−4.059 86	1.625 11
H	3.171 10	−5.050 86	−1.582 47	H	3.150 33	−5.263 18	1.593 26
B	1.681 97	−3.513 97	−1.646 28	N	1.657 15	−3.483 16	1.639 71
N	0.492 65	−4.339 62	−1.636 22	B	0.488 22	−4.339 20	1.607 78
H	0.606 45	−5.348 84	−1.585 36	H	0.624 50	−5.544 45	1.568 84
B	−0.850 84	−3.800 01	−1.648 16	N	−0.842 39	−3.765 92	1.640 36
N	−2.044 68	−4.618 45	−1.629 12	B	−2.016 13	−4.626 48	1.625 28
H	−1.960 10	−5.630 26	−1.586 20	H	−1.900 40	−5.834 45	1.593 37
B	−3.363 48	−4.057 10	−1.609 84	N	−3.314 29	−4.024 60	1.659 60
H	−4.333 04	−4.788 41	−1.570 94	H	−4.124 75	−4.636 82	1.627 44
N	−3.531 75	−2.609 76	−1.639 49	B	−3.513 70	−2.593 52	1.663 68
B	−2.351 70	−1.738 45	−1.660 12	N	−2.341 09	−1.727 27	1.664 78
N	−1.017 31	−2.353 41	−1.665 14	B	−1.008 86	−2.311 45	1.653 51
B	0.173 80	−1.515 79	−1.662 02	N	0.161 05	−1.447 20	1.656 44
N	1.521 73	−2.066 62	−1.663 63	B	1.494 40	−2.028 28	1.653 34
B	2.685 32	−1.169 74	−1.656 23	N	2.662 46	−1.161 25	1.664 97
N	2.550 54	0.293 15	−1.662 92	B	2.502 29	0.284 42	1.653 96
B	3.720 13	1.160 69	−1.644 93	N	3.678 73	1.155 82	1.640 47
N	5.025 78	0.536 07	−1.624 89	B	5.010 98	0.569 60	1.626 12
H	5.859 61	1.115 46	−1.582 10	H	5.999 32	1.273 75	1.594 06
N	3.515 74	2.593 86	−1.633 68	B	3.509 99	2.594 82	1.608 16
H	4.332 77	3.197 12	−1.582 29	H	4.485 55	3.315 51	1.568 34
B	2.206 22	3.211 13	−1.645 59	N	2.184 09	3.179 03	1.640 98
N	1.032 89	2.348 71	−1.663 66	B	1.005 41	2.310 66	1.654 36
B	1.229 60	0.905 96	−1.661 11	N	1.168 83	0.865 43	1.656 80
C	0.002 53	−0.000 06	−1.654 08	B	−0.002 69	0.000 10	1.652 97
B	−1.395 49	0.609 72	−1.662 71	N	−1.337 95	0.581 88	1.657 12
N	−2.546 51	−0.282 21	−1.665 98	B	−2.507 83	−0.282 36	1.653 38
B	−3.880 11	0.302 72	−1.649 72	N	−3.849 20	0.304 28	1.638 31
N	−5.013 72	−0.597 30	−1.631 01	B	−5.016 68	−0.564 69	1.621 83
B	−4.865 47	−2.022 86	−1.610 47	N	−4.823 05	−1.982 29	1.657 50
H	−5.849 82	−2.734 18	−1.571 97	H	−5.646 35	−2.577 13	1.625 19
H	−5.955 84	−0.218 57	−1.589 22	H	−6.137 22	−0.099 03	1.587 79
N	−4.000 43	1.745 52	−1.639 31	B	−4.006 04	1.744 51	1.605 43
H	−4.931 51	2.151 38	−1.589 54	H	−5.117 94	2.228 98	1.563 99
B	−2.861 27	2.639 16	−1.649 15	N	−2.844 35	2.610 22	1.638 73
N	−1.525 15	2.059 98	−1.665 31	B	−1.501 47	2.027 04	1.653 79
B	−0.325 58	2.908 24	−1.658 83	N	−0.329 50	2.888 75	1.665 45
N	−0.490 14	4.365 76	−1.637 13	B	−0.493 37	4.337 30	1.664 65
B	0.684 95	5.227 28	−1.607 37	N	0.690 62	5.165 71	1.660 89
N	1.993 58	4.642 80	−1.626 30	B	2.015 30	4.624 63	1.626 11
H	2.792 69	5.269 18	−1.583 30	H	2.978 83	5.362 31	1.593 83
H	0.561 11	6.435 39	−1.569 16	H	0.587 35	6.176 15	1.628 91
B	−1.827 72	4.943 67	−1.608 40	N	−1.832 53	4.880 26	1.658 50
H	−1.976 31	6.148 98	−1.569 32	H	−1.957 35	5.888 24	1.626 15
N	−2.973 19	4.082 23	−1.629 07	B	−3.002 72	4.056 88	1.622 59
H	−3.891 91	4.514 59	−1.586 62	H	−4.106 72	4.560 53	1.588 04

Table TS15: B97-D3/SVP optimized geometry of the Mg_B(AB)

At.	X	Y	Z	At.	X	Y	Z
N	1.489 29	−5.030 80	−1.696 20	B	−1.716 93	−5.010 21	1.570 67
B	2.268 49	−3.802 28	−1.676 48	N	−2.422 51	−3.737 11	1.612 03
N	3.712 27	−3.827 29	−1.803 76	B	−1.679 82	−2.493 97	1.794 62
B	4.514 62	−2.641 99	−1.961 54	N	−0.229 25	−2.520 43	1.959 65
N	3.868 09	−1.355 85	−1.845 75	B	0.482 36	−3.801 54	1.854 04
B	2.433 79	−1.282 67	−1.472 31	N	−0.293 41	−5.008 52	1.703 30
N	1.914 10	−0.041 64	−1.046 48	H	0.190 48	−5.898 19	1.619 80
Mg	0.129 47	−0.002 22	−0.286 28	N	1.918 07	−3.793 60	1.872 29
N	−0.659 73	1.383 67	−1.488 40	H	2.407 27	−4.671 01	1.717 95
B	0.222 15	2.513 08	−1.622 77	B	2.688 46	−2.578 27	1.902 30
N	1.690 47	2.449 99	−1.550 63	N	4.119 83	−2.560 53	1.744 78
B	2.489 22	1.173 37	−1.474 96	H	4.609 64	−3.437 36	1.590 64
N	3.924 93	1.184 13	−1.847 49	B	4.881 97	−1.351 61	1.672 58
B	4.625 56	2.441 35	−1.967 12	H	6.080 22	−1.391 72	1.496 14
N	3.874 85	3.660 40	−1.810 86	N	4.189 22	−0.080 87	1.813 63
B	2.431 22	3.697 12	−1.683 55	B	2.741 42	−0.051 55	1.980 68
N	1.703 82	4.957 15	−1.702 88	N	1.982 26	−1.301 20	2.065 80
B	0.276 14	5.042 43	−1.745 37	B	0.538 15	−1.296 99	2.067 94
N	−0.495 41	3.809 88	−1.754 63	N	−0.173 78	0.007 32	1.952 17
B	−1.947 52	3.837 22	−1.866 90	B	−1.657 85	0.037 19	1.889 21
N	−2.749 94	2.618 46	−1.894 62	N	−2.380 40	−1.211 69	1.781 31
B	−4.196 17	2.629 31	−2.032 37	B	−3.826 31	−1.182 49	1.534 09
N	−4.896 85	1.375 87	−2.081 91	N	−4.522 77	−2.435 33	1.377 77
B	−4.233 63	0.086 00	−2.009 49	B	−3.861 63	−3.704 22	1.401 75
N	−2.790 72	0.054 58	−1.855 93	H	−4.491 47	−4.727 26	1.243 49
B	−2.110 78	−1.248 03	−1.764 32	H	−5.520 90	−2.425 76	1.187 88
N	−2.862 28	−2.508 84	−1.893 16	N	−4.482 58	0.093 79	1.439 32
B	−2.111 62	−3.760 32	−1.865 51	H	−5.474 50	0.113 57	1.219 56
N	−0.659 77	−3.793 56	−1.750 85	B	−3.775 59	1.342 76	1.532 51
B	0.059 32	−5.057 04	−1.742 18	N	−2.329 36	1.314 09	1.777 90
H	−0.534 74	−6.116 14	−1.787 04	B	−1.577 91	2.567 15	1.789 71
B	0.110 28	−2.527 66	−1.612 57	N	−0.127 22	2.535 18	1.952 46
N	1.580 99	−2.524 58	−1.542 92	B	0.590 26	1.281 98	2.064 15
N	−0.723 60	−1.363 76	−1.470 11	N	2.033 25	1.227 92	2.062 28
H	−2.719 84	−4.807 17	−1.955 14	B	2.790 20	2.475 19	1.896 09
B	−4.307 18	−2.456 84	−2.033 63	N	4.219 65	2.399 47	1.738 70
N	−4.952 44	−1.173 76	−2.081 76	B	4.932 58	1.160 67	1.669 96
H	−5.960 20	−1.160 04	−2.215 16	H	6.131 56	1.152 10	1.494 07
H	−4.965 84	−3.474 34	−2.115 18	H	4.744 56	3.255 63	1.583 92
B	−2.053 39	1.325 45	−1.771 41	N	2.069 47	3.720 66	1.864 24
H	−5.904 33	1.406 32	−2.214 45	H	2.593 78	4.577 23	1.708 25
H	−4.809 72	3.674 78	−2.111 36	B	0.635 28	3.786 55	1.845 74
H	−2.511 50	4.908 74	−1.953 60	N	−0.091 40	5.023 67	1.694 48
H	−0.273 90	6.125 13	−1.787 75	B	−1.514 01	5.082 79	1.565 21
H	2.229 23	5.824 48	−1.776 84	N	−2.270 25	3.839 15	1.608 17
H	4.377 18	4.536 92	−1.924 89	B	−3.709 94	3.864 02	1.400 38
H	5.822 06	2.490 48	−2.179 57	N	−4.421 34	2.622 58	1.376 78
B	4.601 42	−0.101 71	−1.967 33	H	−5.419 43	2.653 10	1.188 90
H	5.797 17	−0.128 48	−2.185 84	H	−4.298 55	4.911 58	1.243 80
H	5.708 24	−2.742 75	−2.171 99	H	−2.075 10	6.146 33	1.415 22
H	4.176 66	−4.724 83	−1.915 25	H	0.427 83	5.893 19	1.610 90
H	1.978 53	−5.919 05	−1.770 31	H	−2.320 10	−6.050 21	1.419 03

Table TS16: B97-D3/SVP optimized geometry of the Mg_B(AA')

At.	X	Y	Z	At.	X	Y	Z
N	−3.651 17	−3.732 83	1.766 58	B	−3.662 95	−3.772 11	−1.550 01
H	−4.508 86	−4.278 40	1.760 87	H	−4.679 34	−4.422 56	−1.427 77
B	−3.742 06	−2.282 73	1.771 18	N	−3.715 18	−2.323 67	−1.672 30
N	−5.013 33	−1.584 24	1.812 76	B	−4.991 09	−1.630 86	−1.560 28
H	−5.870 09	−2.131 24	1.824 28	H	−6.024 80	−2.252 42	−1.433 00
B	−5.137 06	−0.152 66	1.878 33	N	−5.004 19	−0.202 21	−1.610 56
H	−6.237 63	0.360 88	1.950 08	H	−5.896 06	0.268 45	−1.483 72
N	−3.931 01	0.648 66	1.835 38	B	−3.806 77	0.592 48	−1.739 90
B	−3.924 09	2.108 15	1.878 66	N	−3.814 52	2.029 03	−1.701 42
H	−4.985 49	2.697 33	1.970 02	H	−4.698 19	2.504 31	−1.537 42
N	−2.687 78	2.881 02	1.825 82	B	−2.612 49	2.817 26	−1.751 03
B	−2.688 80	4.328 94	1.859 64	N	−2.613 08	4.254 03	−1.627 69
H	−3.725 47	4.961 42	1.934 47	H	−3.497 49	4.736 71	−1.494 17
N	−1.430 87	5.014 62	1.787 29	B	−1.414 78	5.033 99	−1.587 54
H	−1.446 18	6.030 66	1.816 36	H	−1.469 34	6.239 00	−1.461 07
B	−0.145 89	4.343 56	1.726 59	N	−0.133 25	4.355 03	−1.708 53
N	−0.100 23	2.890 27	1.604 55	B	−0.087 36	2.902 93	−1.854 78
B	−1.406 30	2.161 62	1.594 62	N	−1.325 30	2.129 37	−1.914 53
N	−1.520 19	0.777 53	1.298 51	B	−1.306 91	0.684 20	−1.952 59
B	−2.632 06	−0.023 07	1.631 89	N	−2.521 76	−0.101 24	−1.898 42
N	−2.515 33	−1.506 17	1.717 50	B	−2.481 35	−1.557 51	−1.821 20
B	−1.226 48	−2.213 02	1.742 19	N	−1.189 64	−2.244 14	−1.856 16
N	−1.163 12	−3.694 92	1.772 80	B	−1.142 86	−3.703 23	−1.694 32
B	−2.409 48	−4.446 15	1.755 90	N	−2.387 83	−4.421 51	−1.583 71
H	−2.400 76	−5.661 94	1.735 31	H	−2.365 59	−5.429 01	−1.451 93
B	0.136 51	−4.354 88	1.774 76	N	0.141 71	−4.347 96	−1.635 84
H	0.174 27	−5.570 42	1.773 21	H	0.172 77	−5.350 64	−1.470 39
N	1.393 27	−3.616 17	1.781 62	B	1.383 09	−3.623 46	−1.686 49
B	1.360 87	−2.134 32	1.752 06	N	1.338 60	−2.164 32	−1.848 96
N	0.050 27	−1.574 45	1.636 46	B	0.052 46	−1.504 66	−1.926 37
Mg	−0.002 59	−0.016 53	0.367 93	N	0.005 38	−0.019 79	−1.881 38
N	1.449 58	0.862 87	1.336 55	B	1.270 32	0.765 41	−1.946 95
B	1.245 34	2.245 05	1.604 01	N	1.197 65	2.208 83	−1.909 08
N	2.483 94	3.044 44	1.806 51	B	2.438 55	2.977 16	−1.746 82
B	2.393 96	4.490 64	1.828 41	N	2.348 19	4.411 28	−1.628 11
N	1.095 10	5.094 88	1.772 13	B	1.102 58	5.113 84	−1.588 83
H	1.046 60	6.110 01	1.796 82	H	1.081 16	6.320 13	−1.464 54
H	3.390 25	5.186 66	1.883 31	H	3.200 15	4.949 38	−1.495 51
B	3.767 97	2.353 77	1.853 64	N	3.688 07	2.266 33	−1.693 60
H	4.789 46	3.011 57	1.928 29	H	4.539 74	2.797 03	−1.530 21
N	3.871 50	0.897 34	1.824 90	B	3.771 25	0.832 03	−1.728 66
B	2.618 66	0.137 85	1.651 15	N	2.532 48	0.058 52	−1.885 53
N	2.602 38	−1.346 44	1.737 06	B	2.583 90	−1.397 66	−1.808 63
B	3.876 91	−2.041 36	1.788 07	N	3.863 30	−2.084 13	−1.655 09
N	3.879 13	−3.493 98	1.786 80	B	3.901 99	−3.532 93	−1.530 27
B	2.685 24	−4.285 63	1.772 10	N	2.670 58	−4.261 36	−1.567 56
H	2.755 11	−5.499 45	1.753 49	H	2.711 26	−5.268 09	−1.434 26
H	4.769 89	−3.983 70	1.782 38	H	4.957 00	−4.117 55	−1.402 84
N	5.099 49	−1.259 97	1.820 95	B	5.092 92	−1.312 18	−1.543 65
H	5.990 84	−1.748 61	1.831 26	H	6.163 61	−1.867 01	−1.413 82
B	5.128 54	0.177 17	1.866 29	N	5.016 24	0.114 49	−1.597 30
H	6.192 79	0.763 74	1.922 26	H	5.876 69	0.640 67	−1.471 55

Table TS17: B97-D3/SVP optimized geometry of the $Mg_N(AB)$

At.	X	Y	Z	At.	X	Y	Z
N	−3.613 19	3.741 35	1.773 31	B	−5.207 54	1.274 68	−1.660 12
B	−2.167 12	3.776 99	1.797 50	N	−4.508 85	−0.000 27	−1.667 61
N	−1.413 00	5.009 61	1.857 78	B	−3.053 60	−0.000 35	−1.632 86
B	0.019 97	5.041 42	1.862 14	N	−2.266 08	1.202 86	−1.604 53
N	0.750 98	3.784 11	1.831 74	B	−2.972 82	2.466 59	−1.569 12
B	0.017 88	2.519 01	1.782 06	N	−4.421 88	2.470 86	−1.610 91
N	0.745 88	1.260 33	1.753 74	H	−4.911 38	3.360 65	−1.575 37
B	0.017 42	0.000 46	1.742 15	N	−2.175 16	3.640 84	−1.467 34
N	0.745 65	−1.259 53	1.753 81	H	−2.613 56	4.553 74	−1.395 71
B	2.199 71	−1.260 23	1.739 68	B	−0.740 99	3.560 60	−1.445 60
N	2.926 10	0.000 19	1.715 45	N	−0.014 33	4.810 23	−1.367 04
B	2.199 95	1.260 74	1.739 73	H	−0.554 93	5.665 75	−1.279 70
N	2.929 81	2.528 58	1.763 94	B	1.402 14	4.913 43	−1.344 09
B	4.384 31	2.526 52	1.738 48	H	1.953 27	5.986 54	−1.229 12
N	5.073 74	1.270 20	1.720 43	N	2.144 04	3.679 54	−1.470 59
B	4.382 66	0.000 03	1.713 43	B	1.441 13	2.396 51	−1.639 00
N	5.073 49	−1.270 26	1.720 27	N	−0.020 88	2.260 00	−1.507 98
B	4.383 83	−2.526 45	1.738 26	B	−0.814 74	0.984 97	−1.512 45
N	2.929 33	−2.528 21	1.763 77	Mg	0.278 32	0.000 63	−4.422 22
B	2.199 83	−3.780 70	1.809 59	B	−0.814 88	−0.985 97	−1.512 13
N	0.750 26	−3.783 32	1.831 83	N	−2.266 23	−1.203 65	−1.604 10
B	0.019 03	−5.040 47	1.862 80	B	−2.973 10	−2.467 30	−1.568 20
N	−1.413 94	−5.008 39	1.859 26	N	−4.422 16	−2.471 40	−1.609 85
B	−2.167 84	−3.775 66	1.798 56	B	−5.207 68	−1.275 13	−1.659 55
N	−1.437 97	−2.515 77	1.770 55	H	−6.419 41	−1.327 03	−1.687 11
B	−2.167 77	−1.258 46	1.729 51	H	−4.911 80	−3.361 09	−1.574 00
N	−3.631 43	−1.256 23	1.696 90	N	−2.175 55	−3.641 60	−1.466 16
B	−4.352 70	0.000 86	1.666 76	H	−2.614 04	−4.554 42	−1.394 13
N	−3.631 18	1.257 82	1.696 54	B	−0.741 37	−3.561 55	−1.444 67
B	−4.357 29	2.518 77	1.707 79	N	−0.021 14	−2.261 04	−1.507 35
H	−5.569 88	2.540 54	1.681 21	B	1.440 84	−2.397 64	−1.638 69
B	−2.167 53	1.259 77	1.729 33	N	2.238 98	−1.237 13	−1.935 29
N	−1.437 49	2.516 95	1.770 36	B	1.623 39	−0.000 59	−2.356 38
N	−1.439 80	0.000 59	1.734 63	N	2.239 22	1.235 91	−1.935 48
H	−5.563 73	0.000 97	1.629 30	B	3.690 19	1.252 30	−1.739 41
B	−4.357 78	−2.517 03	1.708 82	N	4.338 56	2.522 26	−1.529 72
N	−3.613 90	−3.739 72	1.774 92	B	3.595 53	3.739 27	−1.432 91
H	−4.133 98	−4.612 02	1.812 45	H	4.169 69	4.798 40	−1.297 27
H	−5.570 37	−2.538 57	1.682 32	H	5.347 25	2.562 20	−1.417 16
B	0.017 40	−2.518 06	1.782 16	N	4.399 24	−0.000 75	−1.762 14
H	−1.909 51	−5.894 33	1.907 68	H	5.403 52	−0.000 85	−1.609 72
H	0.605 66	−6.101 77	1.899 62	B	3.690 00	−1.253 69	−1.739 42
H	2.804 84	−4.830 02	1.832 90	N	4.338 26	−2.523 73	−1.529 88
H	5.008 49	−3.566 36	1.747 38	B	3.595 13	−3.740 67	−1.432 98
H	6.089 51	−1.284 98	1.735 99	N	2.143 63	−3.680 76	−1.470 35
H	6.089 76	1.284 73	1.736 17	B	1.401 65	−4.914 56	−1.343 48
H	5.009 17	3.566 31	1.747 65	N	−0.014 83	−4.811 23	−1.366 09
B	2.200 56	3.781 21	1.809 70	H	−0.555 46	−5.666 71	−1.278 48
H	2.805 75	4.830 42	1.833 07	H	1.952 70	−5.987 70	−1.228 45
H	0.606 82	6.102 59	1.899 07	H	4.169 22	−4.799 86	−1.297 50
H	−1.908 42	5.895 66	1.905 79	H	5.346 98	−2.563 78	−1.417 56
H	−4.133 08	4.613 78	1.810 45	H	−6.419 26	1.326 68	−1.687 70

Table TS18: B97-D3/SVP optimized geometry of the Mg_N(AA')

At.	X	Y	Z	At.	X	Y	Z
B	−5.112 60	−1.275 16	−1.696 56	N	−5.137 56	−1.271 83	1.600 66
H	−6.326 14	−1.326 00	−1.708 52	H	−6.150 60	−1.283 51	1.520 86
N	−4.416 95	0.000 64	−1.703 19	B	−4.449 13	−0.001 75	1.638 60
B	−5.112 62	1.276 45	−1.697 09	N	−5.138 17	1.267 94	1.599 23
H	−6.326 17	1.327 28	−1.708 64	H	−6.151 14	1.279 05	1.518 48
N	−4.329 28	2.474 38	−1.675 41	B	−4.450 73	2.523 20	1.600 72
H	−4.817 85	3.364 91	−1.641 49	H	−5.072 72	3.563 88	1.538 78
B	−2.880 57	2.469 69	−1.622 80	N	−2.997 68	2.528 25	1.686 29
N	−2.083 36	3.645 12	−1.530 64	B	−2.265 82	3.778 81	1.688 54
H	−2.522 99	4.559 10	−1.474 46	H	−2.869 03	4.830 16	1.621 67
B	−0.649 44	3.564 53	−1.484 57	N	−0.819 57	3.784 60	1.779 03
N	0.075 23	4.815 58	−1.407 87	B	−0.085 85	5.041 21	1.795 55
H	−0.468 15	5.672 07	−1.347 73	H	−0.672 70	6.103 27	1.749 02
B	1.489 85	4.915 95	−1.330 86	N	1.342 96	5.007 96	1.871 76
H	2.039 29	5.991 56	−1.213 19	H	1.841 53	5.893 29	1.857 28
N	2.236 31	3.682 38	−1.422 16	B	2.097 66	3.775 65	1.860 32
B	1.537 61	2.397 60	−1.608 06	N	1.368 29	2.514 83	1.827 60
N	0.071 81	2.262 32	−1.516 64	B	−0.085 83	2.517 91	1.796 94
B	−0.724 67	0.987 94	−1.521 90	N	−0.814 85	1.259 74	1.777 77
N	−2.174 72	1.204 71	−1.632 36	B	−2.268 37	1.259 62	1.727 84
B	−2.960 97	0.000 64	−1.661 29	N	−2.993 08	−0.001 34	1.708 28
N	−2.174 72	−1.203 44	−1.631 45	B	−2.267 71	−1.261 92	1.728 26
B	−2.880 56	−2.468 45	−1.621 72	N	−2.996 38	−2.530 94	1.687 44
N	−4.329 27	−2.473 10	−1.673 91	B	−4.449 46	−2.526 72	1.602 72
H	−4.817 87	−3.363 61	−1.639 79	H	−5.070 91	−3.567 78	1.541 67
N	−2.083 20	−3.643 83	−1.530 16	B	−2.263 88	−3.781 11	1.689 33
H	−2.522 70	−4.557 87	−1.474 03	H	−2.866 59	−4.832 78	1.622 78
B	−0.649 25	−3.563 16	−1.485 08	N	−0.817 60	−3.786 16	1.778 90
N	0.071 96	−2.260 91	−1.517 26	B	−0.084 51	−2.519 09	1.796 63
B	−0.724 68	−0.986 59	−1.521 73	N	−0.814 18	−1.261 30	1.777 79
Mg	0.434 08	−0.001 80	−4.406 74	B	−0.085 73	−0.000 59	1.776 03
B	1.741 14	0.000 92	−2.320 13	N	1.370 70	−0.000 21	1.776 22
N	2.345 05	1.237 73	−1.881 92	B	2.098 48	1.258 23	1.790 15
B	3.789 73	1.254 00	−1.643 55	N	3.562 73	1.256 64	1.771 24
N	4.433 18	2.524 25	−1.417 46	B	4.286 85	2.519 06	1.794 39
B	3.685 50	3.739 29	−1.337 59	N	3.542 65	3.738 96	1.867 03
H	4.256 09	4.800 82	−1.190 17	H	4.059 13	4.614 01	1.856 90
H	5.438 17	2.561 67	−1.273 30	H	5.500 28	2.540 49	1.766 94
N	4.499 05	0.000 85	−1.641 11	B	4.282 61	0.000 56	1.730 36
H	5.497 31	0.000 85	−1.450 83	H	5.495 33	0.000 90	1.679 38
B	3.789 84	−1.252 36	−1.644 17	N	3.563 39	−1.255 93	1.770 43
N	2.345 14	−1.236 07	−1.882 37	B	2.099 14	−1.258 29	1.789 58
B	1.537 77	−2.396 10	−1.608 98	N	1.369 62	−2.515 28	1.826 67
N	2.236 56	−3.680 94	−1.423 86	B	2.099 66	−3.775 74	1.858 55
B	1.490 17	−4.914 59	−1.332 95	N	1.345 60	−5.008 44	1.869 84
N	0.075 53	−4.814 22	−1.409 26	B	−0.083 22	−5.042 40	1.794 66
H	−0.467 79	−5.670 76	−1.349 32	H	−0.669 58	−6.104 75	1.748 34
H	2.039 72	−5.990 24	−1.216 12	H	1.844 62	−5.893 52	1.854 92
B	3.685 77	−3.737 81	−1.339 54	N	3.544 62	−3.738 31	1.864 65
H	4.256 43	−4.799 39	−1.192 73	H	4.061 54	−4.613 10	1.853 86
N	4.433 39	−2.522 71	−1.418 93	B	4.288 18	−2.517 99	1.792 62
H	5.438 41	−2.560 17	−1.274 91	H	5.501 62	−2.538 76	1.764 90

Table TS19: B97-D3/SVP optimized geometry of the P_B(AB)

At.	X	Y	Z	At.	X	Y	Z
N	−1.417 77	5.021 90	−1.546 36	B	1.474 46	5.048 49	1.762 36
B	−2.175 27	3.789 41	−1.601 96	N	2.200 53	3.789 27	1.747 84
N	−3.621 62	3.757 26	−1.547 25	B	1.464 16	2.521 92	1.700 83
B	−4.377 11	2.540 83	−1.473 15	N	0.009 56	2.521 76	1.680 19
N	−3.664 61	1.272 78	−1.526 28	B	−0.716 20	3.785 85	1.697 18
B	−2.212 69	1.275 83	−1.695 34	N	0.043 58	5.016 82	1.730 11
N	−1.541 00	0.002 47	−1.869 30	H	−0.449 69	5.904 92	1.722 35
P	0.000 78	−0.001 91	−2.693 84	N	−2.156 68	3.759 95	1.683 18
N	0.767 02	−1.338 07	−1.866 32	H	−2.662 03	4.640 91	1.665 30
B	0.000 21	−2.556 47	−1.692 24	B	−2.906 11	2.529 60	1.697 76
N	−1.464 83	−2.523 36	−1.710 45	N	−4.352 35	2.495 05	1.731 37
B	−2.219 62	−1.267 23	−1.695 02	H	−4.870 11	3.369 12	1.723 64
N	−3.671 54	−1.256 60	−1.526 81	B	−5.101 82	1.275 75	1.763 26
B	−4.390 79	−2.520 90	−1.475 12	H	−6.314 14	1.301 79	1.799 15
N	−3.641 63	−3.741 27	−1.548 82	N	−4.381 04	0.013 51	1.748 53
B	−2.195 38	−3.781 07	−1.601 96	B	−2.915 23	0.009 55	1.702 32
N	−1.444 26	−5.017 50	−1.545 79	N	−2.180 97	1.265 16	1.680 61
B	−0.013 15	−5.063 36	−1.469 50	B	−0.723 74	1.262 58	1.665 33
N	0.728 65	−3.812 18	−1.521 48	N	−0.000 19	0.001 59	1.656 54
B	2.181 45	−3.802 84	−1.466 52	B	1.453 66	−0.002 47	1.663 69
N	2.922 52	−2.553 25	−1.521 73	N	2.184 40	1.258 19	1.676 99
B	4.377 06	−2.544 07	−1.470 47	B	3.642 02	1.253 82	1.692 89
N	5.059 53	−1.285 37	−1.547 37	N	4.335 21	2.523 50	1.725 94
B	4.371 04	−0.013 01	−1.603 19	B	3.654 11	3.782 18	1.760 39
N	2.916 51	−0.009 14	−1.710 85	H	4.282 95	4.818 97	1.796 94
B	2.212 87	1.276 35	−1.694 62	H	5.351 04	2.534 80	1.717 29
N	2.936 38	2.535 30	−1.526 60	N	4.332 86	−0.010 37	1.678 18
B	2.201 85	3.788 79	−1.472 20	H	5.348 43	−0.013 18	1.659 17
N	0.749 02	3.805 53	−1.525 15	B	3.635 23	−1.270 76	1.695 98
B	0.013 67	5.060 53	−1.472 72	N	2.177 64	−1.267 13	1.679 28
H	0.594 95	6.121 71	−1.389 94	B	1.450 63	−2.526 81	1.703 89
B	0.013 82	2.553 50	−1.694 35	N	−0.003 90	−2.518 59	1.681 13
N	−1.451 37	2.527 96	−1.711 45	B	−0.730 44	−1.255 43	1.665 21
N	0.774 16	1.330 96	−1.867 65	N	−2.187 66	−1.249 98	1.679 76
H	2.808 70	4.833 52	−1.385 12	B	−2.919 47	−2.510 53	1.695 09
B	4.390 94	2.518 29	−1.476 30	N	−4.365 51	−2.468 14	1.726 99
N	5.066 48	1.255 69	−1.550 61	B	−5.108 47	−1.244 88	1.760 82
H	6.082 25	1.260 59	−1.518 85	H	−6.320 94	−1.264 45	1.796 13
H	5.025 03	3.548 92	−1.395 01	H	−4.888 03	−3.339 35	1.717 76
B	2.205 93	−1.290 78	−1.692 50	N	−2.176 59	−3.744 90	1.680 65
H	6.075 25	−1.295 85	−1.515 35	H	−2.686 70	−4.623 08	1.661 44
H	5.005 34	−3.578 02	−1.386 30	B	−0.736 34	−3.778 71	1.697 70
H	2.782 74	−4.850 60	−1.377 22	N	0.016 68	−5.013 85	1.733 24
H	0.562 50	−6.127 46	−1.384 94	B	1.447 30	−5.053 34	1.768 38
H	−1.947 73	−5.899 69	−1.513 08	N	2.180 12	−3.798 10	1.753 31
H	−4.158 45	−4.615 73	−1.516 57	B	3.633 65	−3.799 01	1.768 06
H	−5.600 50	−2.548 12	−1.393 19	N	4.321 62	−2.544 09	1.732 52
B	−4.383 14	0.010 02	−1.472 48	H	5.337 38	−2.560 90	1.725 09
H	−5.591 28	0.013 33	−1.384 80	H	4.256 70	−4.839 19	1.807 30
H	−5.586 51	2.574 44	−1.389 23	H	2.030 78	−6.116 24	1.807 40
H	−4.133 71	4.634 42	−1.513 38	H	−0.481 43	−5.899 26	1.725 80
H	−1.916 70	5.906 63	−1.512 90	H	2.063 85	6.108 21	1.798 73

Table TS20: B97-D3/SVP optimized geometry of the P_B(AA')

At.	X	Y	Z	At.	X	Y	Z
N	−3.173 05	4.140 92	−1.533 70	B	−3.212 47	4.161 81	1.708 47
H	−3.963 58	4.771 92	−1.434 40	H	−4.149 75	4.934 71	1.687 84
B	−3.421 81	2.718 20	−1.610 52	N	−3.432 77	2.726 07	1.717 50
N	−4.751 40	2.154 21	−1.533 26	B	−4.781 38	2.186 35	1.708 57
H	−5.544 67	2.781 65	−1.433 36	H	−5.746 50	2.924 22	1.688 03
B	−5.001 11	0.746 85	−1.447 64	N	−4.958 99	0.766 51	1.727 34
H	−6.134 04	0.329 20	−1.326 33	H	−5.907 88	0.404 18	1.700 78
N	−3.872 36	−0.169 35	−1.527 04	B	−3.849 54	−0.162 54	1.722 00
B	−4.069 85	−1.606 45	−1.446 46	N	−4.032 42	−1.591 61	1.722 89
H	−5.192 46	−2.049 30	−1.321 36	H	−4.977 26	−1.964 57	1.693 76
N	−2.944 34	−2.521 56	−1.527 78	B	−2.922 40	−2.510 17	1.722 46
B	−3.143 22	−3.961 69	−1.449 14	N	−3.097 82	−3.946 47	1.727 47
H	−4.256 10	−4.430 13	−1.327 69	H	−4.038 34	−4.329 93	1.700 18
N	−1.999 77	−4.819 39	−1.535 67	B	−1.998 02	−4.861 91	1.709 49
H	−2.150 94	−5.819 56	−1.436 92	H	−2.198 48	−6.060 12	1.689 00
B	−0.643 22	−4.323 60	−1.611 55	N	−0.644 61	−4.334 57	1.719 44
N	−0.428 99	−2.885 21	−1.743 71	B	−0.428 86	−2.882 89	1.711 77
B	−1.593 77	−1.996 42	−1.724 15	N	−1.565 85	−1.975 49	1.707 22
N	−1.434 24	−0.566 49	−1.909 41	B	−1.353 10	−0.533 66	1.696 13
B	−2.527 17	0.369 06	−1.723 68	N	−2.493 65	0.373 93	1.707 03
N	−2.283 20	1.813 72	−1.743 69	B	−2.283 37	1.813 23	1.710 67
B	−0.931 11	2.378 09	−1.723 79	N	−0.929 04	2.344 16	1.706 57
N	−0.710 75	3.810 17	−1.526 97	B	−0.713 71	3.786 21	1.721 62
B	−1.858 54	4.702 38	−1.447 82	N	−1.869 80	4.656 42	1.727 26
H	−1.707 76	5.900 38	−1.326 31	H	−1.731 65	5.662 69	1.700 71
B	0.644 53	4.327 29	−1.445 56	N	0.636 79	4.288 14	1.721 86
H	0.822 18	5.520 82	−1.319 15	H	0.786 12	5.292 88	1.692 45
N	1.790 46	3.437 95	−1.527 39	B	1.782 97	3.415 16	1.721 63
B	1.584 21	2.003 68	−1.724 02	N	1.569 60	1.972 84	1.706 34
N	0.227 41	1.524 87	−1.909 15	B	0.213 38	1.438 91	1.695 72
P	0.000 49	−0.000 55	−2.736 52	N	−0.000 36	0.000 43	1.689 19
N	1.208 04	−0.959 95	−1.909 19	B	1.138 61	−0.903 96	1.696 58
B	0.944 18	−2.374 26	−1.723 42	N	0.922 82	−2.345 30	1.708 17
N	2.083 08	−3.269 95	−1.526 04	B	2.065 37	−3.251 13	1.724 05
B	1.853 81	−4.705 60	−1.446 98	N	1.815 48	−4.676 36	1.729 93
N	0.510 13	−5.192 97	−1.533 69	B	0.497 11	−5.232 51	1.710 97
H	0.363 36	−6.193 85	−1.435 35	H	0.340 71	−6.437 29	1.691 23
H	2.782 00	−5.477 92	−1.325 90	H	2.603 83	−5.316 84	1.703 93
B	3.426 32	−2.722 24	−1.444 50	N	3.394 49	−2.694 99	1.724 70
H	4.371 15	−3.472 79	−1.318 26	H	4.189 90	−3.326 77	1.695 32
N	3.656 08	−1.290 05	−1.526 84	B	3.635 09	−1.274 53	1.723 78
B	2.526 05	−0.383 05	−1.724 00	N	2.493 71	−0.367 20	1.707 71
N	2.713 35	1.070 03	−1.744 29	B	2.711 04	1.071 03	1.710 83
B	4.066 06	1.603 77	−1.611 63	N	4.076 18	1.610 18	1.718 03
N	4.242 30	3.037 26	−1.534 35	B	4.283 03	3.047 84	1.708 91
B	3.148 30	3.957 28	−1.448 52	N	3.142 16	3.911 52	1.727 32
H	3.353 01	5.147 25	−1.327 02	H	3.302 54	4.914 48	1.700 73
H	5.182 37	3.410 60	−1.435 16	H	5.404 62	3.514 63	1.688 93
N	5.173 72	0.676 85	−1.534 32	B	5.209 65	0.701 77	1.709 92
H	6.115 38	1.046 10	−1.434 87	H	6.347 61	1.127 17	1.689 62
B	5.002 83	−0.742 24	−1.448 43	N	4.966 73	−0.708 23	1.729 36
H	5.964 92	−1.471 80	−1.326 66	H	5.769 05	−1.331 12	1.703 02

Table TS21: B97-D3/SVP optimized geometry of the P_N(AB)

At.	X	Y	Z	At.	X	Y	Z
B	−3.295 66	−4.091 14	−1.422 92	N	−4.925 22	−1.708 95	1.803 02
N	−1.850 85	−3.966 85	−1.563 10	B	−4.347 54	−0.383 56	1.767 01
B	−1.236 46	−2.650 26	−1.762 84	N	−5.147 12	0.821 01	1.805 84
N	−2.105 39	−1.464 18	−1.816 25	B	−4.570 74	2.132 83	1.786 12
B	−3.538 60	−1.582 20	−1.555 41	N	−3.122 27	2.261 85	1.745 66
N	−4.098 61	−2.906 18	−1.406 72	B	−2.285 40	1.062 77	1.695 51
H	−5.096 23	−3.012 34	−1.246 88	N	−0.836 67	1.190 04	1.659 32
N	−4.329 67	−0.375 49	−1.480 14	B	−0.001 39	−0.002 36	1.648 98
H	−5.322 98	−0.462 27	−1.285 23	N	1.448 91	0.124 73	1.659 48
B	−3.759 51	0.950 09	−1.554 03	B	2.062 96	1.442 96	1.695 85
N	−4.540 31	2.156 91	−1.403 65	N	1.226 89	2.633 05	1.709 73
H	−5.541 27	2.088 62	−1.244 03	B	−0.222 18	2.508 06	1.697 47
B	−3.954 43	3.462 96	−1.417 16	N	−1.063 24	3.704 17	1.748 32
H	−4.653 29	4.445 36	−1.287 80	B	−0.447 15	5.021 31	1.791 74
N	−2.509 98	3.590 78	−1.557 40	N	0.982 66	5.114 79	1.813 54
B	−1.677 11	2.400 71	−1.759 86	B	1.841 61	3.951 90	1.772 71
N	−2.327 40	1.082 28	−1.814 51	N	3.284 64	4.041 95	1.808 35
B	−1.608 17	−0.137 72	−2.152 56	B	4.132 43	2.886 89	1.783 91
P	0.001 44	0.003 07	−3.142 95	N	3.519 87	1.568 08	1.743 65
B	0.927 77	−1.321 14	−2.152 99	B	4.348 16	0.378 97	1.753 63
N	0.230 64	−2.554 25	−1.816 36	N	3.739 53	−0.936 17	1.745 49
B	1.060 85	−3.728 46	−1.555 32	B	4.572 27	−2.128 23	1.787 91
N	0.405 89	−5.008 14	−1.406 08	N	3.938 51	−3.413 25	1.811 06
B	−1.018 07	−5.153 94	−1.422 26	B	2.501 85	−3.575 71	1.772 92
H	−1.519 58	−6.250 48	−1.294 58	N	1.667 00	−2.383 95	1.710 50
H	0.965 32	−5.840 90	−1.246 06	B	0.218 28	−2.512 96	1.696 32
N	2.493 83	−3.559 50	−1.479 85	N	−0.401 78	−3.837 26	1.744 74
H	3.065 43	−4.376 38	−1.284 59	B	−1.845 78	−3.959 97	1.752 11
B	3.143 44	−2.271 02	−1.554 33	N	−2.680 39	−2.775 28	1.741 05
N	2.324 96	−1.088 66	−1.815 64	B	−4.129 23	−2.900 36	1.780 84
B	2.917 64	0.256 78	−1.761 09	H	−4.660 39	−3.990 49	1.812 95
N	2.101 01	1.479 40	−1.814 50	B	−2.065 03	−1.448 78	1.693 42
B	0.684 57	1.466 93	−2.151 80	N	−2.897 91	−0.256 41	1.706 19
N	−0.215 49	2.560 27	−1.813 69	N	−0.616 24	−1.322 00	1.658 38
B	0.398 97	3.860 06	−1.551 09	H	−2.357 50	−5.057 83	1.776 04
N	−0.467 47	5.006 64	−1.398 83	B	0.434 12	−5.027 11	1.787 29
B	−1.895 14	4.903 74	−1.413 91	N	1.858 37	−4.870 41	1.810 70
H	−2.578 79	5.896 55	−1.282 89	H	2.428 74	−5.708 81	1.877 92
H	−0.060 48	5.922 95	−1.235 39	H	−0.059 23	−6.134 86	1.820 49
N	1.839 61	3.941 77	−1.476 85	B	2.283 09	−1.066 47	1.696 87
H	2.261 08	4.844 77	−1.279 11	H	4.531 57	−4.235 75	1.878 36
B	2.702 52	2.785 33	−1.552 27	H	5.781 89	−2.043 00	1.821 51
N	4.138 08	2.857 84	−1.401 56	H	5.554 78	0.484 85	1.777 67
B	4.976 37	1.697 56	−1.417 75	H	5.338 47	3.013 57	1.814 66
N	4.364 86	0.382 86	−1.559 74	H	3.725 80	4.955 13	1.873 21
B	5.194 64	−0.806 27	−1.418 89	H	1.398 65	6.039 52	1.881 06
N	4.569 87	−2.094 11	−1.403 56	H	−1.125 65	6.026 28	1.827 01
H	5.160 52	−2.905 04	−1.243 58	B	−2.506 59	3.573 70	1.757 47
H	6.396 58	−0.710 92	−1.290 44	H	−3.201 49	4.565 75	1.783 44
H	6.176 66	1.811 46	−1.289 14	H	−5.283 45	3.113 86	1.820 88
H	4.579 37	3.758 60	−1.240 41	H	−6.158 43	0.746 07	1.872 05
H	−3.814 03	−5.179 86	−1.295 73	H	−5.934 15	−1.811 36	1.868 84

Table TS22: B97-D3/SVP optimized geometry of the P_N(AA')

At.	X	Y	Z	At.	X	Y	Z
B	5.086 55	1.312 58	−1.404 05	N	5.039 07	1.322 18	1.815 59
H	6.292 97	1.334 07	−1.267 81	H	6.054 99	1.343 80	1.811 09
N	4.379 95	0.047 64	−1.549 67	B	4.362 46	0.045 66	1.795 74
B	5.113 54	−1.201 86	−1.404 30	N	5.066 02	−1.216 20	1.815 73
H	6.320 18	−1.197 52	−1.268 50	H	6.082 17	−1.216 30	1.812 52
N	4.395 91	−2.440 06	−1.408 24	B	4.393 55	−2.478 13	1.758 42
H	4.920 54	−3.295 06	−1.245 78	H	5.029 86	−3.512 14	1.739 18
B	2.960 39	−2.506 44	−1.561 19	N	2.938 85	−2.499 03	1.736 76
N	2.214 78	−3.741 91	−1.487 26	B	2.222 65	−3.757 79	1.706 29
H	2.722 81	−4.600 33	−1.291 26	H	2.840 65	−4.802 37	1.672 75
B	0.772 95	−3.800 86	−1.561 24	N	0.774 57	−3.779 47	1.736 68
N	0.023 79	−5.027 23	−1.408 87	B	0.055 79	−5.044 37	1.758 37
H	0.520 77	−5.898 66	−1.246 73	H	0.655 70	−6.099 94	1.739 35
B	−1.406 96	−5.060 48	−1.405 30	N	−1.374 00	−5.026 13	1.815 71
H	−1.991 44	−6.116 24	−1.270 17	H	−1.863 19	−5.916 78	1.812 17
N	−2.149 20	−3.816 00	−1.550 07	B	−2.141 20	−3.801 89	1.795 46
B	−1.436 23	−2.549 96	−1.760 49	N	−1.426 44	−2.532 91	1.744 01
N	0.033 62	−2.566 57	−1.820 91	B	0.027 69	−2.520 46	1.723 26
B	0.823 06	−1.390 16	−2.158 59	N	0.741 17	−1.253 71	1.698 78
N	2.234 28	−1.264 37	−1.821 01	B	2.194 98	−1.238 23	1.723 38
B	2.927 10	0.032 01	−1.760 48	N	2.906 08	0.030 22	1.744 46
N	2.206 70	1.313 33	−1.820 87	B	2.168 24	1.283 30	1.723 40
B	2.905 94	2.570 69	−1.560 71	N	2.885 10	2.559 57	1.736 74
N	4.342 53	2.535 07	−1.407 70	B	4.339 94	2.569 57	1.758 08
H	4.848 68	3.401 12	−1.245 07	H	4.954 14	3.616 87	1.738 66
N	2.133 96	3.789 81	−1.486 27	B	2.142 26	3.802 83	1.706 77
H	2.623 39	4.658 90	−1.289 97	H	2.737 90	4.860 32	1.673 48
B	0.691 22	3.817 86	−1.560 50	N	0.694 04	3.793 68	1.737 58
N	−0.021 45	2.567 94	−1.820 05	B	−0.025 85	2.519 11	1.723 81
B	0.793 10	1.408 87	−2.158 25	N	0.714 43	1.267 79	1.698 92
P	−0.000 01	0.000 86	−3.148 60	B	−0.000 07	−0.000 63	1.693 12
B	−1.615 90	−0.016 64	−2.158 28	N	−1.455 86	−0.016 07	1.698 70
N	−2.212 53	−1.301 75	−1.820 87	B	−2.169 38	−1.282 81	1.722 99
B	−3.651 28	−1.309 53	−1.561 17	N	−3.633 18	−1.296 65	1.735 98
N	−4.311 57	−2.585 95	−1.408 80	B	−4.342 37	−2.566 93	1.757 38
B	−3.598 17	−3.826 60	−1.405 39	N	−3.585 78	−3.780 28	1.815 24
H	−4.197 96	−4.873 76	−1.270 49	H	−4.093 95	−4.660 24	1.811 57
H	−5.314 38	−2.612 81	−1.246 63	H	−5.556 02	−2.600 98	1.737 83
N	−4.348 38	−0.046 06	−1.486 84	B	−4.365 20	−0.047 02	1.705 63
H	−5.345 80	−0.056 81	−1.290 83	H	−5.578 82	−0.059 89	1.671 81
B	−3.678 57	1.232 10	−1.560 97	N	−3.659 93	1.217 86	1.736 85
N	−2.239 94	1.255 22	−1.820 24	B	−2.196 19	1.235 21	1.723 55
B	−1.490 56	2.519 77	−1.759 50	N	−1.479 94	2.500 72	1.745 01
N	−2.230 46	3.770 17	−1.548 82	B	−2.221 55	3.754 20	1.797 00
B	−1.515 16	5.030 29	−1.403 87	N	−1.480 56	4.994 42	1.817 51
N	−0.084 01	5.027 92	−1.407 99	B	−0.051 48	5.043 02	1.759 79
H	0.394 18	5.909 77	−1.245 73	H	0.525 87	6.111 10	1.741 12
H	−2.122 15	6.073 20	−1.268 27	H	−1.988 57	5.874 47	1.814 35
B	−3.679 40	3.749 75	−1.404 67	N	−3.665 34	3.701 88	1.817 11
H	−4.301 48	4.783 84	−1.269 86	H	−4.192 09	4.570 84	1.814 11
N	−4.366 11	2.494 08	−1.408 60	B	−4.396 01	2.472 79	1.759 14
H	−5.369 37	2.499 46	−1.247 03	H	−5.610 11	2.481 03	1.740 13

Table TS23: B97-D3/SVP optimized geometry of the Layer(AA')

At.	X	Y	Z	At.	X	Y	Z
N	−4.147 02	−3.161 68	1.660 91	B	−4.193 30	−3.166 85	−1.607 65
H	−5.076 06	−3.571 63	1.622 61	H	−5.300 61	−3.665 10	−1.568 77
B	−4.012 96	−1.722 75	1.670 15	N	−4.027 97	−1.723 50	−1.639 28
N	−5.147 45	−0.827 59	1.662 22	B	−5.187 16	−0.847 92	−1.606 56
H	−6.084 97	−1.217 74	1.623 76	H	−6.311 54	−1.306 31	−1.567 55
B	−5.010 61	0.596 52	1.625 00	N	−4.985 12	0.568 72	−1.623 40
H	−5.994 24	1.306 91	1.584 98	H	−5.804 60	1.167 69	−1.577 01
N	−3.675 02	1.174 68	1.646 44	B	−3.671 00	1.173 58	−1.645 23
B	−3.497 19	2.612 43	1.610 46	N	−3.470 94	2.600 41	−1.630 46
H	−4.468 09	3.338 99	1.561 55	H	−4.283 43	3.208 31	−1.574 20
N	−2.168 18	3.188 75	1.648 44	B	−2.158 67	3.195 08	−1.643 45
B	−1.990 54	4.633 32	1.628 93	N	−1.949 43	4.626 54	−1.620 37
H	−2.949 69	5.376 51	1.589 81	H	−2.755 40	5.243 63	−1.574 33
N	−0.663 07	5.166 61	1.666 78	B	−0.647 51	5.220 12	−1.600 46
H	−0.553 63	6.176 26	1.630 52	H	−0.525 54	6.428 14	−1.559 03
B	0.516 05	4.331 12	1.675 39	N	0.519 87	4.355 60	−1.633 50
N	0.343 42	2.883 66	1.682 21	B	0.346 10	2.898 65	−1.660 12
B	−0.994 72	2.313 53	1.669 36	N	−0.990 03	2.322 11	−1.668 36
N	−1.167 05	0.869 07	1.674 40	B	−1.164 98	0.875 35	−1.674 48
B	−2.504 04	0.296 15	1.667 77	N	−2.503 43	0.299 15	−1.670 55
N	−2.673 24	−1.148 61	1.678 80	B	−2.679 42	−1.145 54	−1.664 03
B	−1.510 29	−2.022 25	1.666 54	N	−1.511 92	−2.014 29	−1.671 65
N	−1.681 59	−3.476 18	1.644 39	B	−1.683 80	−3.462 93	−1.647 42
B	−3.021 35	−4.044 54	1.622 52	N	−3.028 06	−3.997 42	−1.625 19
H	−3.185 29	−5.246 74	1.581 45	H	−3.159 38	−5.003 98	−1.579 46
B	−0.517 83	−4.338 81	1.606 77	N	−0.512 60	−4.301 99	−1.633 25
H	−0.661 33	−5.542 93	1.557 20	H	−0.632 70	−5.309 59	−1.577 07
N	0.816 12	−3.774 02	1.644 83	B	0.823 05	−3.761 85	−1.647 48
B	0.991 58	−2.320 60	1.666 89	N	0.996 73	−2.313 44	−1.671 35
N	−0.173 25	−1.449 29	1.673 16	B	−0.171 59	−1.442 46	−1.676 02
B	−0.000 68	−0.003 12	1.674 48	N	0.000 77	0.003 28	−1.678 79
N	1.337 80	0.570 42	1.674 76	B	1.338 89	0.576 66	−1.674 19
B	1.510 05	2.014 92	1.669 90	N	1.509 17	2.024 02	−1.668 39
N	2.856 37	2.589 67	1.649 32	B	2.850 29	2.597 73	−1.644 64
B	3.023 46	4.035 57	1.629 71	N	2.983 61	4.038 28	−1.622 54
N	1.858 50	4.866 01	1.667 02	B	1.857 80	4.921 38	−1.601 58
H	1.989 32	5.873 11	1.630 61	H	2.023 26	6.124 22	−1.560 22
H	4.130 45	4.532 36	1.590 71	H	3.912 11	4.448 52	−1.577 12
B	4.012 71	1.717 00	1.612 04	N	3.985 92	1.711 15	−1.631 92
H	5.127 26	2.194 79	1.563 59	H	4.918 62	2.110 98	−1.576 88
N	3.847 36	0.277 70	1.647 90	B	3.844 80	0.277 31	−1.645 53
B	2.502 66	−0.300 85	1.668 51	N	2.504 40	−0.298 08	−1.669 76
N	2.327 30	−1.744 90	1.679 57	B	2.335 74	−1.743 62	−1.663 31
B	3.494 54	−2.617 98	1.671 68	N	3.510 61	−2.622 50	−1.638 35
N	3.286 44	−4.048 05	1.662 14	B	3.331 72	−4.064 16	−1.607 12
B	1.984 75	−4.641 44	1.623 56	N	2.003 94	−4.597 48	−1.625 61
H	1.861 55	−5.848 51	1.582 95	H	1.894 99	−5.606 70	−1.579 92
H	4.093 05	−4.665 05	1.625 23	H	4.290 70	−4.808 91	−1.567 45
N	4.807 68	−2.014 71	1.663 77	B	4.843 07	−2.043 93	−1.605 65
H	5.627 23	−2.614 40	1.626 79	H	5.828 16	−2.753 74	−1.565 55
B	5.009 64	−0.598 34	1.627 16	N	4.979 88	−0.619 57	−1.623 99
H	6.132 78	−0.139 20	1.588 33	H	5.917 23	−0.230 07	−1.577 71

Table TS24: B97-D3/SVP optimized geometry of the Si_B(AB)

At.	X	Y	Z	At.	X	Y	Z
N	1.124 51	5.104 15	−1.526 52	B	3.683 31	3.752 15	1.763 28
B	−0.125 37	4.372 80	−1.587 14	N	3.728 37	2.299 37	1.742 34
N	−1.415 20	5.031 20	−1.527 04	B	2.481 12	1.530 32	1.685 29
B	−2.665 15	4.328 59	−1.467 13	N	1.198 91	2.217 05	1.659 73
N	−2.647 60	2.875 92	−1.544 00	B	1.156 34	3.673 94	1.688 52
B	−1.368 04	2.192 10	−1.726 68	N	2.407 22	4.399 95	1.729 99
N	−1.409 78	0.760 52	−1.931 59	H	2.391 75	5.415 75	1.727 85
Si	0.000 22	−0.000 64	−2.599 93	N	−0.125 51	4.331 21	1.678 53
N	0.046 34	−1.602 01	−1.930 91	H	−0.154 87	5.346 47	1.668 38
B	−1.214 28	−2.281 60	−1.725 65	B	−1.367 23	3.600 91	1.688 20
N	−2.485 83	−1.532 54	−1.714 24	N	−2.657 98	4.253 38	1.729 34
B	−2.583 48	−0.060 02	−1.726 30	H	−2.701 29	5.268 37	1.727 12
N	−3.857 45	0.634 17	−1.543 82	B	−3.894 51	3.532 87	1.762 37
B	−5.081 38	−0.148 49	−1.467 11	H	−4.950 56	4.128 29	1.805 49
N	−4.982 61	−1.578 96	−1.527 06	N	−3.855 55	2.079 97	1.741 54
B	−3.724 28	−2.295 76	−1.586 91	B	−2.565 93	1.384 28	1.684 81
N	−3.649 54	−3.741 94	−1.526 19	N	−1.325 55	2.143 99	1.659 53
B	−2.416 06	−4.473 03	−1.465 49	B	−0.042 31	1.454 09	1.637 02
N	−1.166 77	−3.731 50	−1.542 39	N	−0.000 29	0.000 37	1.617 33
B	0.126 73	−4.403 34	−1.484 86	B	1.279 72	−0.690 05	1.636 77
N	1.379 58	−3.658 42	−1.542 66	N	2.518 85	0.076 31	1.659 18
B	2.669 40	−4.326 99	−1.465 74	B	3.801 45	−0.616 04	1.687 75
N	3.858 89	−3.526 29	−1.526 40	N	5.011 88	0.175 58	1.729 30
B	3.850 54	−2.078 18	−1.586 97	B	5.006 13	1.606 69	1.762 89
N	2.570 32	−1.387 35	−1.714 60	H	6.049 79	2.223 52	1.806 44
B	2.583 22	0.088 38	−1.726 59	H	5.912 58	−0.294 34	1.727 15
N	3.815 13	0.854 57	−1.543 48	N	3.813 03	−2.056 54	1.677 91
B	3.750 13	2.310 66	−1.485 84	H	4.706 97	−2.538 71	1.667 68
N	2.478 55	3.023 13	−1.543 25	B	2.602 85	−2.838 00	1.688 13
B	2.412 69	4.474 42	−1.466 21	N	1.319 85	−2.146 44	1.659 41
H	3.419 41	5.144 82	−1.374 54	B	0.084 00	−2.913 48	1.685 45
B	1.240 42	2.266 96	−1.726 41	N	−1.194 10	−2.219 17	1.659 50
N	−0.083 58	2.918 71	−1.714 74	B	−1.238 24	−0.762 93	1.636 65
N	1.364 37	0.840 27	−1.931 93	N	−2.519 57	−0.069 50	1.658 87
H	4.778 25	2.944 39	−1.385 51	B	−3.760 06	−0.834 78	1.687 49
B	5.081 97	0.143 48	−1.466 53	N	−5.014 23	−0.114 41	1.728 65
N	5.065 60	−1.290 31	−1.526 74	B	−5.091 17	1.314 62	1.761 95
H	5.959 32	−1.772 13	−1.479 53	H	−6.168 75	1.870 11	1.805 00
H	6.133 30	0.741 54	−1.374 91	H	−5.886 24	−0.635 64	1.726 24
B	1.343 87	−2.208 13	−1.725 90	N	−3.688 33	−2.273 53	1.677 97
H	4.752 22	−4.008 84	−1.479 27	H	−4.552 85	−2.806 66	1.668 21
H	2.746 61	−5.534 02	−1.373 86	B	−2.435 00	−2.983 73	1.688 25
H	0.161 40	−5.610 59	−1.384 44	N	−2.354 66	−4.427 78	1.730 05
H	−2.423 83	−5.682 51	−1.373 59	B	−1.112 41	−5.138 35	1.763 77
H	−4.513 70	−4.274 96	−1.479 19	N	0.126 35	−4.378 12	1.742 87
H	−5.847 16	−2.111 37	−1.480 01	B	1.407 01	−5.065 45	1.763 88
H	−6.165 35	0.388 18	−1.375 94	N	2.606 12	−4.284 28	1.730 10
B	−3.876 17	2.091 64	−1.486 56	H	3.493 54	−4.778 81	1.728 08
H	−4.939 05	2.665 26	−1.386 71	H	1.464 75	−6.276 37	1.807 72
H	−3.708 73	4.940 11	−1.375 86	H	−1.100 04	−6.350 60	1.807 50
H	−1.444 70	6.046 09	−1.479 95	H	−3.211 97	−4.972 82	1.727 62
H	1.095 72	6.119 07	−1.479 33	H	4.703 13	4.407 66	1.806 71

Table TS25: B97-D3/SVP optimized geometry of the Si_B(AA')

At.	X	Y	Z	At.	X	Y	Z
N	1.655 89	4.952 57	−1.513 88	B	1.651 60	4.991 52	1.714 06
H	1.729 27	5.960 03	−1.400 29	H	1.767 23	6.200 89	1.701 68
B	0.337 73	4.359 61	−1.598 15	N	0.338 58	4.370 33	1.716 67
N	−0.873 16	5.148 92	−1.514 55	B	−0.863 42	5.185 82	1.712 89
H	−0.790 19	6.155 63	−1.400 99	H	−0.791 94	6.398 62	1.701 15
B	−2.186 98	4.581 20	−1.442 52	N	−2.139 58	4.538 45	1.727 32
H	−3.159 77	5.295 06	−1.311 18	H	−2.970 89	5.122 18	1.705 03
N	−2.325 41	3.136 50	−1.547 95	B	−2.288 60	3.099 35	1.712 40
B	−3.626 36	2.485 30	−1.461 81	N	−3.575 17	2.451 09	1.712 78
H	−4.620 65	3.167 35	−1.321 92	H	−4.413 18	3.025 38	1.689 32
N	−3.765 20	1.036 97	−1.546 56	B	−3.715 16	1.017 27	1.713 47
B	−5.062 90	0.387 46	−1.438 22	N	−5.003 33	0.358 72	1.729 87
H	−6.078 88	1.037 92	−1.304 75	H	−5.847 77	0.923 31	1.707 70
N	−5.119 37	−1.042 73	−1.509 76	B	−5.146 27	−1.065 06	1.716 66
H	−6.028 16	−1.482 89	−1.393 12	H	−6.251 41	−1.569 73	1.706 09
B	−3.946 95	−1.887 95	−1.594 82	N	−3.951 66	−1.891 37	1.720 09
N	−2.635 40	−1.260 90	−1.750 81	B	−2.628 19	−1.257 68	1.699 04
B	−2.575 53	0.213 30	−1.761 94	N	−2.512 68	0.192 46	1.689 86
N	−1.323 93	0.905 99	−1.984 11	B	−1.199 26	0.823 36	1.673 19
B	−1.128 51	2.323 00	−1.761 89	N	−1.085 09	2.276 01	1.688 85
N	0.225 14	2.910 00	−1.751 28	B	0.225 49	2.907 31	1.697 48
B	1.471 80	2.121 00	−1.761 77	N	1.423 62	2.082 28	1.689 44
N	2.779 99	2.739 86	−1.547 51	B	2.739 21	2.711 08	1.713 70
B	2.866 35	4.188 72	−1.441 93	N	2.813 08	4.155 90	1.730 09
H	3.937 71	4.743 74	−1.310 36	H	3.724 27	4.604 91	1.708 44
B	3.964 85	1.895 53	−1.462 24	N	3.910 97	1.873 08	1.714 30
H	5.052 62	2.415 80	−1.322 89	H	4.827 29	2.311 81	1.691 67
N	3.878 31	0.443 12	−1.547 19	B	3.829 12	0.434 82	1.713 08
B	2.575 47	−0.186 88	−1.761 46	N	2.514 36	−0.195 67	1.689 03
N	1.445 77	0.690 66	−1.983 20	B	1.313 26	0.629 36	1.673 29
Si	−0.000 99	−0.001 18	−2.653 65	N	0.001 09	0.001 17	1.657 05
N	−0.125 31	−1.600 25	−1.983 68	B	−0.111 00	−1.449 31	1.673 38
B	−1.450 19	−2.139 67	−1.761 43	N	−1.426 05	−2.076 82	1.690 66
N	−1.556 13	−3.582 82	−1.546 93	B	−1.537 33	−3.530 74	1.715 54
B	−2.876 44	−4.185 13	−1.438 83	N	−2.857 99	−4.121 37	1.732 82
N	−4.025 09	−3.331 15	−1.509 60	B	−4.056 82	−3.340 14	1.718 46
H	−4.938 23	−3.762 27	−1.393 19	H	−5.142 82	−3.884 79	1.708 36
H	−3.008 03	−5.384 37	−1.306 03	H	−2.947 60	−5.133 22	1.711 83
B	−0.341 56	−4.384 06	−1.462 55	N	−0.332 65	−4.320 77	1.715 40
H	−0.434 90	−5.586 19	−1.322 86	H	−0.410 88	−5.333 68	1.692 93
N	0.982 13	−3.780 12	−1.548 01	B	0.979 03	−3.725 03	1.713 65
B	1.100 45	−2.337 83	−1.762 21	N	1.092 21	−2.271 27	1.689 25
N	2.407 13	−1.652 64	−1.751 44	B	2.405 81	−1.646 30	1.697 13
B	3.606 15	−2.474 83	−1.597 25	N	3.616 39	−2.475 61	1.716 64
N	4.895 11	−1.820 80	−1.512 29	B	4.923 64	−1.842 43	1.713 60
B	5.060 30	−0.399 04	−1.440 57	N	5.000 97	−0.413 62	1.728 74
H	6.164 70	0.086 67	−1.308 31	H	5.922 09	0.014 60	1.706 88
H	5.725 11	−2.396 14	−1.396 85	H	5.938 23	−2.510 71	1.701 56
N	3.460 44	−3.912 92	−1.513 70	B	3.497 92	−3.923 34	1.714 07
H	4.296 13	−4.480 10	−1.398 91	H	4.487 50	−4.628 14	1.702 41
B	2.193 70	−4.579 29	−1.441 90	N	2.193 42	−4.511 38	1.729 53
H	2.138 59	−5.784 60	−1.310 23	H	2.126 67	−5.524 99	1.708 27

Table TS26: B97-D3/SVP optimized geometry of the Si_N(AB)

At.	X	Y	Z	At.	X	Y	Z
B	0.624 45	5.207 26	−1.385 27	N	3.273 13	4.056 53	1.827 61
N	−0.530 53	4.338 09	−1.575 69	B	3.484 55	2.626 27	1.788 82
B	−0.355 41	2.905 78	−1.852 66	N	4.800 80	2.028 20	1.828 06
N	1.026 35	2.378 46	−1.927 00	B	5.006 15	0.609 93	1.811 18
B	2.165 60	3.222 51	−1.566 71	N	3.845 88	−0.266 35	1.769 11
N	1.935 11	4.634 16	−1.364 12	B	2.501 39	0.307 52	1.713 31
H	2.717 93	5.246 52	−1.153 72	N	1.340 05	−0.567 48	1.671 49
N	3.472 44	2.611 91	−1.462 21	B	−0.000 05	0.001 74	1.658 85
H	4.257 40	3.202 85	−1.203 17	N	−1.163 20	−0.874 15	1.670 84
B	3.697 79	1.187 16	−1.566 72	B	−0.986 00	−2.317 34	1.713 05
N	4.990 62	0.575 35	−1.363 83	N	0.353 47	−2.884 03	1.726 31
H	5.795 71	1.158 24	−1.153 83	B	1.516 67	−2.010 72	1.714 04
B	5.178 59	−0.842 77	−1.383 73	N	2.863 15	−2.579 87	1.770 55
H	6.283 36	−1.311 58	−1.211 25	B	3.038 01	−4.023 25	1.814 35
N	4.023 76	−1.712 17	−1.573 52	N	1.874 73	−4.860 09	1.832 37
B	2.695 78	−1.147 79	−1.850 74	B	0.530 38	−4.328 11	1.791 87
N	2.571 38	0.325 79	−1.926 31	N	−0.645 82	−5.168 94	1.830 96
B	1.359 41	1.021 16	−2.353 90	B	−1.976 70	−4.637 70	1.812 15
Si	0.000 08	−0.002 54	−3.347 00	N	−2.155 37	−3.194 76	1.768 56
B	−1.565 68	0.663 68	−2.353 74	B	−3.487 56	−2.624 76	1.781 97
N	−1.569 36	2.061 18	−1.926 80	N	−3.667 61	−1.187 00	1.768 14
B	−2.878 26	2.605 87	−1.565 84	B	−5.005 05	−0.616 77	1.810 75
N	−2.994 76	4.031 42	−1.363 23	N	−5.148 20	0.809 09	1.828 59
B	−1.860 79	4.903 45	−1.384 93	B	−4.015 26	1.707 31	1.789 20
H	−2.007 19	6.094 67	−1.212 74	N	−2.676 24	1.138 52	1.725 20
H	−3.901 97	4.437 10	−1.152 47	B	−1.515 57	2.015 14	1.712 74
N	−3.999 38	1.698 39	−1.460 42	N	−1.690 92	3.466 52	1.768 13
H	−4.903 35	2.082 73	−1.200 53	B	−0.531 14	4.335 29	1.781 58
B	−3.874 93	0.261 47	−1.565 07	N	0.804 03	3.772 28	1.767 92
N	−2.574 41	−0.303 13	−1.925 57	B	1.966 69	4.645 44	1.810 26
B	−2.340 26	−1.763 21	−1.850 73	H	1.840 21	5.851 32	1.847 61
N	−1.001 85	−2.392 32	−1.924 85	B	0.984 50	2.321 54	1.712 55
B	0.206 60	−1.690 78	−2.352 51	N	2.322 37	1.751 00	1.724 86
N	1.548 47	−2.080 80	−1.924 89	N	−0.177 21	1.447 00	1.670 72
B	1.709 85	−3.489 09	−1.563 09	H	−0.678 34	5.537 36	1.811 88
N	3.047 52	−3.995 41	−1.360 39	B	−3.029 88	4.033 23	1.810 66
B	4.199 15	−3.146 87	−1.382 37	N	−4.155 48	3.146 27	1.828 24
H	5.303 34	−3.617 07	−1.210 03	H	−5.083 19	3.555 25	1.898 42
H	3.186 54	−4.979 35	−1.149 29	H	−3.198 13	5.234 00	1.848 15
N	0.527 46	−4.315 06	−1.457 55	B	−2.501 35	−0.305 46	1.712 73
H	0.646 39	−5.289 87	−1.196 67	H	−6.086 65	1.192 81	1.899 11
B	−0.818 95	−3.797 99	−1.563 25	H	−5.986 09	−1.329 24	1.848 81
N	−1.995 27	−4.611 54	−1.360 37	H	−4.454 96	−3.353 30	1.811 99
B	−3.317 45	−4.065 60	−1.382 06	H	−2.932 50	−5.383 70	1.850 07
N	−3.493 12	−2.630 98	−1.573 64	H	−0.536 29	−6.176 81	1.902 17
B	−4.823 33	−2.065 38	−1.383 35	H	2.011 61	−5.864 61	1.903 67
N	−4.982 35	−0.643 78	−1.362 27	H	4.145 52	−4.516 63	1.853 04
H	−5.904 09	−0.271 98	−1.152 02	B	4.018 35	−1.705 08	1.783 97
H	−5.782 79	−2.786 50	−1.211 63	H	5.132 97	−2.178 58	1.814 79
H	−4.275 81	−4.788 01	−1.209 63	H	6.130 16	0.155 31	1.849 33
H	−1.893 14	−5.599 90	−1.148 72	H	5.618 85	2.627 17	1.898 26
H	0.479 73	6.398 63	−1.212 86	H	4.074 67	4.677 43	1.897 68

Table TS27: B97-D3/SVP optimized geometry of the Si_N(AA')

At.	X	Y	Z	At.	X	Y	Z
B	−3.348 76	4.035 01	−1.379 16	N	−3.345 25	3.995 71	1.840 05
H	−3.874 18	5.116 70	−1.211 64	H	−3.797 97	4.905 44	1.838 93
N	−1.907 14	3.933 62	−1.567 67	B	−1.902 22	3.927 75	1.817 09
B	−1.093 80	5.128 16	−1.379 08	N	−1.060 90	5.102 07	1.840 92
H	−1.617 55	6.210 80	−1.212 31	H	−1.494 04	6.021 28	1.839 71
N	0.332 04	5.013 63	−1.369 63	B	0.367 00	5.031 72	1.779 03
H	0.876 57	5.846 10	−1.161 71	H	1.030 97	6.048 41	1.759 84
B	1.006 07	3.751 27	−1.567 49	N	1.006 24	3.725 18	1.752 86
N	2.440 29	3.596 48	−1.461 44	B	2.449 91	3.613 70	1.721 62
H	2.992 56	4.411 15	−1.206 35	H	3.131 45	4.618 21	1.686 25
B	3.115 04	2.321 42	−1.567 00	N	3.087 02	2.313 47	1.753 78
N	4.537 15	2.162 56	−1.368 55	B	4.537 32	2.202 36	1.780 70
H	5.108 85	2.976 66	−1.160 78	H	5.236 64	3.195 07	1.761 90
B	5.171 47	0.880 49	−1.377 69	N	5.130 29	0.901 56	1.843 19
H	6.371 06	0.794 81	−1.210 71	H	6.144 49	0.838 76	1.843 12
N	4.362 87	−0.317 37	−1.566 03	B	4.350 02	−0.314 19	1.819 78
B	2.922 54	−0.213 20	−1.844 94	N	2.897 81	−0.208 75	1.759 31
N	2.327 75	1.140 44	−1.922 61	B	2.266 67	1.101 20	1.736 65
B	0.955 40	1.405 98	−2.349 92	N	0.816 80	1.206 63	1.707 14
N	0.200 50	2.582 64	−1.923 20	B	0.182 99	2.514 84	1.735 97
B	−1.277 19	2.634 16	−1.846 44	N	−1.267 52	2.617 28	1.757 61
N	−2.152 08	1.442 18	−1.923 53	B	−2.086 43	1.415 66	1.735 85
B	−3.568 57	1.533 75	−1.568 24	N	−3.546 51	1.520 06	1.752 65
N	−4.142 08	2.844 81	−1.370 45	B	−4.175 32	2.831 71	1.778 57
H	−5.133 00	2.932 96	−1.162 92	H	−5.384 69	2.941 09	1.759 62
N	−4.335 55	0.312 01	−1.462 38	B	−4.353 99	0.318 14	1.721 86
H	−5.317 24	0.383 12	−1.207 42	H	−5.564 72	0.406 11	1.687 09
B	−3.752 43	−1.007 49	−1.567 00	N	−3.728 70	−0.987 87	1.753 81
N	−2.337 63	−1.121 09	−1.922 72	B	−2.268 87	−1.095 67	1.736 57
B	−1.695 96	0.120 70	−2.350 07	N	−1.452 83	0.107 34	1.707 20
Si	0.000 93	−0.002 30	−3.344 09	B	−0.000 65	0.001 81	1.700 32
B	0.742 68	−1.532 63	−2.348 90	N	0.634 02	−1.308 55	1.707 74
N	2.138 94	−1.467 09	−1.921 56	B	2.083 90	−1.413 76	1.737 45
B	2.748 09	−2.748 93	−1.565 39	N	2.720 45	−2.731 82	1.755 40
N	4.178 24	−2.796 44	−1.367 07	B	4.171 57	−2.831 38	1.782 52
B	4.990 47	−1.619 01	−1.376 58	N	4.946 36	−1.629 91	1.844 25
H	6.189 85	−1.706 78	−1.209 16	H	5.958 99	−1.714 34	1.844 17
H	4.626 80	−3.684 29	−1.159 03	H	4.720 13	−3.914 73	1.764 49
N	1.896 97	−3.913 64	−1.459 46	B	1.902 06	−3.926 34	1.724 16
H	2.326 30	−4.799 12	−1.203 79	H	2.431 29	−5.018 83	1.690 02
B	0.455 39	−3.860 36	−1.565 09	N	0.457 43	−3.828 01	1.755 11
N	−0.173 61	−2.588 17	−1.921 56	B	−0.182 21	−2.511 43	1.737 18
B	−1.643 36	−2.426 50	−1.844 71	N	−1.632 32	−2.403 04	1.758 94
N	−2.453 76	−3.621 59	−1.564 79	B	−2.449 78	−3.607 93	1.819 71
B	−3.894 84	−3.514 25	−1.375 68	N	−3.887 46	−3.466 50	1.843 74
N	−4.508 47	−2.222 17	−1.367 34	B	−4.540 51	−2.194 78	1.781 24
H	−5.501 66	−2.166 61	−1.159 37	H	−5.753 00	−2.128 14	1.762 74
H	−4.570 54	−4.508 94	−1.207 67	H	−4.466 92	−4.301 22	1.844 03
B	−1.820 73	−4.920 56	−1.375 10	N	−1.787 07	−4.891 54	1.843 97
H	−2.494 79	−5.916 41	−1.207 38	H	−2.348 55	−5.738 46	1.843 89
N	−0.393 28	−5.012 29	−1.365 61	B	−0.363 99	−5.028 41	1.782 28
H	0.025 86	−5.914 31	−1.157 18	H	0.146 07	−6.130 40	1.764 40

Table TS28: B97-D3/SVP optimized geometry of the V_B(AB)

At.	X	Y	Z	At.	X	Y	Z
B	−3.796 94	3.645 55	−1.614 16	N	−1.270 92	5.041 75	1.699 64
N	−3.799 24	2.192 14	−1.631 86	B	0.000 06	4.351 40	1.685 85
B	−2.527 82	1.459 84	−1.633 15	N	1.270 37	5.042 87	1.701 16
N	−1.266 83	2.185 37	−1.628 39	B	2.531 14	4.362 85	1.676 91
B	−1.267 74	3.642 51	−1.606 41	N	2.557 95	2.907 38	1.667 40
N	−2.540 64	4.331 43	−1.597 87	B	1.259 66	2.178 37	1.660 03
H	−2.555 11	5.346 72	−1.564 39	N	1.424 75	0.773 37	1.645 38
N	−0.005 76	4.338 35	−1.586 79	N	0.002 62	−1.539 53	1.661 02
H	−0.006 72	5.353 09	−1.539 95	B	1.291 70	−2.145 36	1.656 87
B	1.257 48	3.644 74	−1.605 74	N	2.567 49	−1.465 18	1.655 49
N	2.529 18	4.335 84	−1.596 40	B	2.583 32	−0.019 70	1.650 73
H	2.541 83	5.351 09	−1.561 53	N	3.855 61	0.737 76	1.655 09
B	3.786 68	3.652 18	−1.613 82	B	5.101 84	−0.011 11	1.650 35
H	4.825 47	4.279 35	−1.607 52	N	5.056 99	−1.445 41	1.668 66
N	3.791 59	2.198 80	−1.633 12	B	3.820 46	−2.198 05	1.662 89
B	2.521 46	1.464 26	−1.634 11	N	3.762 83	−3.642 51	1.678 24
N	1.259 20	2.187 60	−1.628 30	B	2.524 85	−4.361 01	1.662 49
B	−0.003 17	1.458 36	−1.630 33	N	1.261 90	−3.632 92	1.663 28
N	−0.001 88	0.004 64	−1.643 02	B	0.003 88	−4.347 63	1.657 89
B	−1.260 20	−0.722 97	−1.630 82	N	−1.254 77	−3.634 05	1.665 14
N	−2.523 25	0.004 87	−1.633 58	B	−2.517 07	−4.363 26	1.666 45
B	−3.784 32	−0.725 14	−1.620 00	N	−3.755 65	−3.645 85	1.683 84
N	−5.018 91	0.030 10	−1.616 43	B	−3.814 60	−2.201 43	1.667 82
B	−5.055 40	1.461 21	−1.624 42	N	−2.562 29	−1.467 45	1.658 36
H	−6.118 06	2.047 03	−1.619 72	B	−2.579 41	−0.021 97	1.651 86
H	−5.904 64	−0.466 77	−1.589 88	N	−3.852 37	0.734 32	1.657 07
N	−3.753 52	−2.166 18	−1.602 62	B	−3.817 85	2.185 14	1.654 40
H	−4.632 25	−2.674 23	−1.562 89	N	−2.556 64	2.905 12	1.666 13
B	−2.519 74	−2.910 63	−1.611 65	B	−2.531 08	4.360 63	1.674 50
N	−1.258 11	−2.181 27	−1.626 18	H	−3.567 56	4.991 78	1.677 10
B	0.000 67	−2.910 53	−1.627 78	B	−1.257 71	2.177 25	1.659 09
N	1.258 18	−2.179 09	−1.627 67	N	0.000 65	2.896 45	1.668 46
B	1.257 72	−0.720 77	−1.631 90	N	−1.421 50	0.772 07	1.643 45
N	2.519 48	0.009 28	−1.635 41	H	−4.859 28	2.804 62	1.651 00
B	3.781 84	−0.718 53	−1.623 66	B	−5.097 94	−0.015 65	1.654 99
N	5.015 10	0.038 88	−1.620 83	N	−5.051 78	−1.449 90	1.674 72
B	5.049 03	1.470 07	−1.627 44	H	−5.935 52	−1.950 90	1.700 68
H	6.110 65	2.057 78	−1.623 10	H	−6.164 75	0.563 07	1.653 64
H	5.901 73	−0.456 43	−1.595 49	B	−1.285 91	−2.146 53	1.658 48
N	3.753 59	−2.159 65	−1.607 23	H	−4.617 01	−4.184 05	1.710 62
H	4.633 26	−2.666 18	−1.568 87	H	−2.514 84	−5.576 62	1.670 03
B	2.521 10	−2.906 24	−1.614 94	H	0.004 42	−5.558 77	1.655 21
N	2.483 79	−4.353 31	−1.603 24	H	2.523 69	−5.574 36	1.665 71
B	1.262 95	−5.100 52	−1.611 26	H	4.624 70	−4.179 96	1.703 56
N	0.001 94	−4.377 96	−1.623 42	H	5.941 21	−1.945 63	1.692 91
B	−1.257 80	−5.102 71	−1.609 41	H	6.168 14	0.568 56	1.647 90
N	−2.479 93	−4.357 62	−1.599 72	B	3.819 79	2.188 52	1.654 00
H	−3.352 26	−4.877 51	−1.570 42	H	4.860 66	2.808 95	1.650 25
H	−1.279 47	−6.315 98	−1.600 80	H	3.567 11	4.994 83	1.681 23
H	1.286 75	−6.313 75	−1.602 67	H	1.281 58	6.058 45	1.733 17
H	3.357 06	−4.871 69	−1.575 07	H	−1.283 07	6.057 33	1.731 16
H	−4.836 85	4.270 86	−1.608 19				

Table TS29: B97-D3/SVP optimized geometry of the V_B(AA')

At.	X	Y	Z	At.	X	Y	Z
B	−3.789 28	−3.650 34	−1.590 26	N	−3.759 10	−3.633 31	1.674 89
H	−4.829 84	−4.277 06	−1.556 54	H	−4.621 98	−4.167 78	1.628 49
N	−3.796 61	−2.198 20	−1.620 90	B	−3.817 82	−2.190 06	1.683 26
B	−5.051 20	−1.465 65	−1.600 43	N	−5.053 08	−1.438 19	1.662 52
H	−6.114 07	−2.053 71	−1.568 71	H	−5.934 47	−1.941 52	1.612 68
N	−5.018 15	−0.035 29	−1.619 28	B	−5.098 77	−0.006 17	1.624 56
H	−5.903 10	0.462 79	−1.583 77	H	−6.164 37	0.573 97	1.571 86
B	−3.783 65	0.720 94	−1.633 18	N	−3.854 37	0.746 27	1.661 53
N	−3.754 71	2.161 54	−1.622 26	B	−3.816 71	2.195 97	1.619 75
H	−4.633 84	2.669 14	−1.574 89	H	−4.858 51	2.815 75	1.561 39
B	−2.521 32	2.907 01	−1.630 70	N	−2.557 80	2.917 93	1.661 55
N	−2.483 86	4.353 94	−1.612 69	B	−2.528 95	4.373 75	1.630 59
H	−3.357 59	4.871 11	−1.572 69	H	−3.565 83	5.003 94	1.583 66
B	−1.261 45	5.097 76	−1.594 43	N	−1.270 40	5.053 58	1.667 59
H	−1.283 67	6.312 14	−1.557 79	H	−1.278 88	6.068 62	1.620 88
N	0.000 05	4.377 67	−1.621 45	B	−0.000 54	4.363 90	1.681 52
B	0.000 06	2.909 24	−1.640 86	N	−0.000 39	2.907 69	1.702 93
N	−1.258 21	2.179 23	−1.646 95	B	−1.259 21	2.189 56	1.699 08
B	−1.259 45	0.721 13	−1.644 33	N	−1.424 58	0.784 69	1.728 77
N	−2.521 89	−0.009 22	−1.647 01	B	−2.581 65	−0.010 40	1.700 72
B	−2.524 87	−1.463 48	−1.638 29	N	−2.564 30	−1.455 79	1.705 54
N	−1.263 43	−2.188 20	−1.638 73	B	−1.289 01	−2.136 57	1.700 39
B	−1.262 02	−3.645 67	−1.617 73	N	−1.258 58	−3.624 27	1.674 21
N	−2.533 70	−4.337 03	−1.600 79	B	−2.522 90	−4.350 78	1.645 18
H	−2.544 66	−5.352 24	−1.559 67	H	−2.519 99	−5.564 28	1.605 98
N	0.000 09	−4.341 08	−1.602 86	B	0.000 40	−4.335 84	1.639 42
H	0.000 09	−5.356 11	−1.553 22	H	0.000 53	−5.547 89	1.595 45
B	1.262 20	−3.645 66	−1.617 79	N	1.259 23	−3.624 00	1.674 08
N	1.263 61	−2.188 20	−1.638 73	B	1.289 32	−2.136 30	1.700 40
B	0.000 09	−1.459 72	−1.639 14	N	0.000 09	−1.530 99	1.712 68
N	0.000 09	−0.005 91	−1.652 06	N	1.424 22	0.784 99	1.728 93
B	1.259 61	0.721 13	−1.644 17	B	1.258 57	2.189 82	1.699 24
N	1.258 35	2.179 23	−1.646 74	N	2.557 01	2.918 49	1.661 85
B	2.521 45	2.907 02	−1.630 27	B	2.527 86	4.374 30	1.630 87
N	2.483 95	4.353 95	−1.612 24	N	1.269 16	5.053 85	1.667 67
B	1.261 54	5.097 76	−1.594 19	H	1.277 43	6.068 90	1.620 88
H	1.283 75	6.312 14	−1.557 56	H	3.564 61	5.004 72	1.584 07
H	3.357 67	4.871 14	−1.572 02	B	3.816 07	2.196 81	1.620 11
N	3.754 84	2.161 55	−1.621 67	H	4.857 75	2.816 81	1.561 84
H	4.633 96	2.669 17	−1.574 21	N	3.854 02	0.747 12	1.661 84
B	3.783 82	0.720 96	−1.632 73	B	2.581 48	−0.009 83	1.700 97
N	2.522 07	−0.009 21	−1.646 76	N	2.564 45	−1.455 22	1.705 70
B	2.525 05	−1.463 47	−1.638 22	B	3.818 13	−2.189 21	1.683 31
N	3.796 79	−2.198 21	−1.621 01	N	3.759 74	−3.632 46	1.674 57
B	3.789 46	−3.650 37	−1.590 52	B	2.523 70	−4.350 22	1.644 82
N	2.533 89	−4.337 04	−1.600 96	H	2.521 08	−5.563 71	1.605 49
H	2.544 83	−5.352 26	−1.560 00	H	4.622 75	−4.166 72	1.628 13
H	4.830 02	−4.277 11	−1.556 99	N	5.053 22	−1.437 06	1.662 52
B	5.051 38	−1.465 66	−1.600 60	H	5.934 71	−1.940 17	1.612 29
H	6.114 27	−2.053 74	−1.569 38	B	5.098 57	−0.005 02	1.624 55
N	5.018 32	−0.035 29	−1.618 92	H	6.164 02	0.575 34	1.571 48
H	5.903 28	0.462 77	−1.583 34				

Table TS30: B97-D3/SVP optimized geometry of the V_N(AB)

At.	X	Y	Z	At.	X	Y	Z
N	−5.069 35	1.270 63	−1.550 02	B	−3.611 09	3.757 49	1.624 19
B	−4.378 64	0.000 14	−1.555 76	N	−2.160 04	3.716 24	1.595 56
N	−5.069 43	−1.270 33	−1.549 27	B	−1.443 30	2.440 95	1.702 02
B	−4.380 52	−2.526 74	−1.580 65	N	−2.203 55	1.240 13	1.960 56
N	−2.926 85	−2.528 58	−1.630 68	B	−3.668 49	1.254 76	1.869 94
B	−2.196 27	−1.260 42	−1.618 63	N	−4.332 26	2.526 98	1.738 06
N	−0.743 31	−1.259 62	−1.651 29	H	−5.346 42	2.559 55	1.687 97
B	−0.013 68	−0.000 05	−1.639 96	N	−4.370 02	−0.000 13	1.904 00
N	1.443 25	−0.000 12	−1.638 83	H	−5.382 10	−0.000 23	1.816 48
B	2.170 22	−1.258 83	−1.626 04	B	−3.668 37	−1.254 98	1.870 21
N	1.440 16	−2.515 89	−1.677 66	N	−4.332 04	−2.527 26	1.738 65
B	−0.014 98	−2.517 88	−1.686 60	H	−5.346 19	−2.559 93	1.688 53
N	−0.748 97	−3.782 95	−1.737 55	B	−3.610 76	−3.757 69	1.624 37
B	−0.017 39	−5.039 90	−1.778 81	H	−4.205 66	−4.810 33	1.535 04
N	1.415 53	−5.007 79	−1.777 58	N	−2.159 72	−3.716 27	1.595 59
B	2.169 88	−3.775 45	−1.705 54	B	−1.443 10	−2.440 92	1.702 11
N	3.615 77	−3.740 29	−1.668 84	N	−2.203 42	−1.240 18	1.960 84
B	4.359 17	−2.518 33	−1.582 42	B	−1.531 97	0.000 03	2.186 89
N	3.633 42	−1.257 34	−1.573 06	B	0.757 61	1.022 95	1.609 60
B	4.354 24	−0.000 23	−1.528 48	N	0.002 49	2.312 49	1.555 45
N	3.633 53	1.256 93	−1.573 42	B	0.726 14	3.602 55	1.499 73
B	4.359 37	2.517 87	−1.582 94	N	−0.003 31	4.852 51	1.423 48
N	3.616 05	3.739 89	−1.669 18	B	−1.420 71	4.953 40	1.452 51
B	2.170 16	3.775 18	−1.705 55	H	−1.979 17	6.025 60	1.367 17
N	1.440 36	2.515 66	−1.677 69	H	0.534 11	5.709 51	1.331 28
B	−0.014 77	2.517 78	−1.686 51	N	2.158 03	3.655 79	1.534 62
N	−0.748 65	3.782 91	−1.737 26	H	2.620 38	4.555 82	1.453 68
B	−2.197 23	3.781 02	−1.694 96	B	2.927 92	2.466 16	1.694 03
N	−2.926 63	2.528 69	−1.630 98	N	2.210 29	1.204 87	1.756 24
B	−4.380 32	2.526 99	−1.581 24	B	2.995 34	0.000 17	1.803 26
H	−5.005 03	3.566 97	−1.579 11	N	2.210 36	−1.204 57	1.756 44
B	−2.196 16	1.260 47	−1.618 77	B	0.757 71	−1.022 76	1.609 65
N	−2.922 60	0.000 06	−1.582 70	N	0.002 66	−2.312 36	1.555 45
N	−0.743 20	1.259 57	−1.651 30	B	0.726 41	−3.602 35	1.499 70
H	−2.802 64	4.830 43	−1.714 04	N	−0.002 94	−4.852 34	1.422 94
B	−0.017 00	5.039 83	−1.778 39	B	−1.420 32	−4.953 34	1.452 10
N	1.415 91	5.007 60	−1.777 23	H	−1.978 72	−6.025 55	1.366 58
H	1.911 55	5.893 25	−1.829 90	H	0.534 51	−5.709 29	1.330 40
H	−0.603 64	6.101 05	−1.821 27	N	2.158 29	−3.655 52	1.535 05
B	2.170 32	1.258 54	−1.626 17	H	2.620 70	−4.555 54	1.454 30
H	4.136 33	4.612 10	−1.705 71	B	2.928 06	−2.465 84	1.694 60
H	5.571 53	2.540 58	−1.538 69	N	4.375 77	−2.465 77	1.763 88
H	5.564 44	−0.000 26	−1.468 40	B	5.157 00	−1.269 85	1.843 45
H	5.571 31	−2.541 13	−1.537 88	N	4.452 63	0.000 20	1.853 86
H	4.135 97	−4.612 56	−1.705 27	B	5.156 94	1.270 28	1.842 85
H	1.911 07	−5.893 50	−1.830 34	N	4.375 64	2.466 14	1.762 89
H	−0.604 09	−6.101 07	−1.821 91	H	4.866 86	3.354 45	1.716 62
B	−2.197 54	−3.780 94	−1.695 02	H	6.368 39	1.319 06	1.888 66
H	−2.803 02	−4.830 31	−1.714 20	H	6.368 45	−1.318 52	1.889 40
H	−5.005 32	−3.566 68	−1.578 36	H	4.867 05	−3.354 08	1.718 02
H	−6.085 56	−1.285 02	−1.545 29	H	−4.206 06	4.810 12	1.535 02
H	−6.085 47	1.285 39	−1.545 90				

Table TS31: B97-D3/SVP optimized geometry of the V_N(AA')

At.	X	Y	Z	At.	X	Y	Z
N	3.641 47	3.742 63	−1.644 15	B	3.604 66	3.753 01	1.725 96
H	4.160 18	4.613 10	−1.566 47	H	4.198 25	4.811 94	1.764 89
B	2.197 21	3.776 89	−1.594 09	N	2.157 49	3.710 52	1.831 65
N	1.444 36	5.002 48	−1.449 32	B	1.415 10	4.946 71	1.962 41
H	1.943 91	5.881 77	−1.348 78	H	1.973 38	6.023 47	2.015 60
B	0.014 64	5.031 83	−1.394 02	N	−0.002 24	4.847 32	2.006 05
H	−0.573 29	6.085 47	−1.260 40	H	−0.539 79	5.709 36	2.035 06
N	−0.717 08	3.779 53	−1.512 79	B	−0.734 12	3.597 68	1.917 99
B	−2.166 50	3.773 28	−1.511 02	N	−2.163 75	3.656 62	1.815 51
H	−2.774 00	4.819 16	−1.409 67	H	−2.620 13	4.563 86	1.797 44
N	−2.894 21	2.528 08	−1.644 75	B	−2.931 38	2.466 08	1.646 09
B	−4.349 22	2.522 84	−1.686 91	N	−4.375 09	2.467 41	1.511 34
H	−4.975 84	3.561 66	−1.645 08	H	−4.860 99	3.359 06	1.473 87
N	−5.033 07	1.269 38	−1.792 61	B	−5.152 13	1.270 57	1.412 12
H	−6.049 20	1.280 47	−1.795 07	H	−6.360 92	1.319 65	1.304 28
B	−4.343 85	−0.001 29	−1.779 51	N	−4.454 21	−0.001 04	1.460 58
N	−2.887 77	−0.000 95	−1.736 01	B	−2.999 30	−0.000 60	1.564 88
B	−2.163 80	1.259 67	−1.689 70	N	−2.215 52	1.203 11	1.632 73
N	−0.710 11	1.259 90	−1.691 84	B	−0.768 21	1.014 88	1.831 95
B	0.015 72	2.517 17	−1.631 05	N	−0.009 96	2.306 37	1.895 41
N	1.469 57	2.517 45	−1.682 19	B	1.435 62	2.435 52	1.741 71
B	2.199 09	1.261 55	−1.766 89	N	2.193 05	1.237 90	1.467 93
N	3.663 59	1.259 95	−1.788 75	B	3.660 18	1.253 87	1.487 19
B	4.385 44	2.522 46	−1.719 15	N	4.327 38	2.526 59	1.587 95
H	5.599 65	2.543 22	−1.718 28	H	5.341 38	2.564 94	1.535 85
B	4.382 82	0.000 73	−1.790 66	N	4.360 73	0.001 54	1.406 43
H	5.596 58	0.001 01	−1.789 30	H	5.376 26	0.001 87	1.378 28
N	3.664 17	−1.258 82	−1.788 47	B	3.660 99	−1.251 25	1.487 26
B	2.199 67	−1.261 09	−1.766 61	N	2.193 88	−1.236 17	1.467 92
N	1.474 88	0.000 06	−1.795 18	B	1.512 27	0.000 63	1.269 05
B	0.018 12	−0.000 27	−1.722 37	B	−0.767 46	−1.014 55	1.831 93
N	−0.709 52	−1.260 78	−1.691 56	N	−2.214 68	−1.203 78	1.632 69
B	−2.163 22	−1.261 22	−1.689 39	B	−2.929 79	−2.467 17	1.646 39
N	−2.893 05	−2.529 95	−1.644 14	N	−4.373 55	−2.469 40	1.511 81
B	−4.348 06	−2.525 40	−1.686 25	B	−5.151 35	−1.273 10	1.412 33
N	−5.032 49	−1.272 27	−1.792 22	H	−6.360 10	−1.322 94	1.304 49
H	−6.048 61	−1.283 84	−1.794 61	H	−4.858 86	−3.361 38	1.474 60
H	−4.974 20	−3.564 49	−1.644 13	N	−2.161 52	−3.657 24	1.816 11
B	−2.164 76	−3.774 83	−1.510 58	H	−2.617 38	−4.564 75	1.798 34
H	−2.771 78	−4.820 98	−1.409 12	B	−0.731 93	−3.597 42	1.918 68
N	−0.715 34	−3.780 43	−1.512 61	N	−0.008 52	−2.305 73	1.895 66
B	0.016 88	−2.517 71	−1.630 71	B	1.437 11	−2.434 15	1.741 92
N	1.470 73	−2.517 31	−1.681 75	N	2.159 75	−3.708 70	1.832 24
B	2.198 94	−3.776 44	−1.594 04	B	3.606 94	−3.750 36	1.726 52
N	3.643 18	−3.741 55	−1.644 15	N	4.328 95	−2.523 54	1.588 21
B	4.386 59	−2.521 02	−1.719 01	H	5.342 98	−2.561 32	1.536 02
H	5.600 82	−2.541 23	−1.718 27	H	4.201 15	−4.808 92	1.765 64
H	4.162 32	−4.611 84	−1.567 31	B	1.418 08	−4.945 21	1.964 13
N	1.446 65	−5.002 44	−1.449 81	H	1.976 97	−6.021 58	2.019 03
H	1.946 60	−5.881 58	−1.350 10	N	0.000 68	−4.846 59	2.007 50
B	0.016 94	−5.032 48	−1.394 65	H	−0.536 40	−5.708 89	2.037 55
H	−0.570 49	−6.086 50	−1.261 85				

Table TS32: B97-D3/SVP optimized geometry of the Al_B

At.	X	Y	Z	At.	X	Y	Z
N	−1.116 311 9	−5.079 111 3	−0.144 217 4				
H	−1.067 317 4	−6.075 676 3	−0.341 707 7				
B	0.124 570 4	−4.349 569 8	0.030 992 2				
N	1.405 055 4	−5.007 036 7	−0.143 910 5				
H	1.412 836 7	−6.004 666 4	−0.342 036 8				
B	2.659 270 0	−4.319 026 4	−0.180 216 9				
H	3.687 774 9	−4.918 514 1	−0.417 684 0				
N	2.656 612 5	−2.886 255 3	0.065 538 0				
B	3.876 149 5	−2.092 390 1	−0.056 342 5				
H	4.922 244 7	−2.657 079 7	−0.301 160 7				
N	3.870 775 0	−0.637 280 9	0.066 287 2				
B	5.070 131 1	0.146 840 0	−0.178 566 6				
H	6.135 743 1	−0.383 806 6	−0.416 206 9				
N	4.957 010 4	1.572 927 5	−0.140 890 6				
H	5.795 493 9	2.114 007 0	−0.337 825 9				
B	3.704 508 9	2.282 530 1	0.033 777 8				
N	2.485 705 2	1.531 431 6	0.358 396 7				
B	2.621 710 3	0.043 737 5	0.424 010 3				
N	1.519 200 2	−0.820 335 6	0.783 892 5				
B	1.401 758 9	−2.215 862 2	0.423 041 4				
N	0.083 519 5	−2.918 590 2	0.356 567 9				
B	−1.272 811 8	−2.292 431 5	0.422 632 2				
N	−2.487 184 2	−3.033 383 9	0.064 817 6				
B	−2.407 865 2	−4.463 895 2	−0.180 776 6				
H	−3.400 321 3	−5.121 317 5	−0.418 192 3				
B	−3.750 120 5	−2.310 541 1	−0.057 180 6				
H	−4.762 143 9	−2.934 034 6	−0.302 388 1				
N	−3.827 858 9	−0.857 558 2	0.065 593 8				
B	−2.619 849 0	−0.106 229 3	0.423 462 4				
N	−1.469 723 9	−0.905 826 1	0.783 362 0				
Al	0.000 020 2	−0.000 207 8	1.079 167 7				
N	−0.049 454 5	1.725 604 3	0.784 563 1				
B	1.217 994 0	2.321 692 8	0.424 281 6				
N	1.171 226 4	3.743 676 8	0.066 594 2				
B	2.410 728 4	4.462 391 2	−0.178 458 0				
N	3.633 694 4	3.720 259 4	−0.141 032 4				
H	4.493 941 9	4.225 854 2	−0.338 469 4				
H	2.415 793 3	5.652 745 1	−0.416 229 4				
B	−0.126 011 4	4.402 848 2	−0.055 700 8				
H	−0.160 157 3	5.591 109 7	−0.300 538 8				
N	−1.383 481 0	3.670 643 1	0.066 604 4				
B	−1.348 888 9	2.248 364 2	0.424 178 5				
N	−2.569 283 3	1.386 839 4	0.358 021 5				
B	−3.829 074 6	2.066 975 2	0.033 421 7				
N	−5.038 811 2	1.286 860 4	−0.141 446 2				
B	−5.070 065 3	−0.143 347 7	−0.179 285 1				
H	−6.103 497 7	−0.734 106 3	−0.417 161 6				
H	−5.906 866 3	1.779 039 2	−0.338 478 9				
N	−3.840 669 3	3.506 507 9	−0.141 002 3				
H	−4.728 363 5	3.962 291 4	−0.338 208 9				
B	−2.662 078 7	4.317 330 6	−0.178 309 3				
H	−2.735 258 4	5.505 574 0	−0.415 579 1				

Table TS33: B97-D3/SVP optimized geometry of the Al_N

At.	X	Y	Z	At.	X	Y	Z
B	−4.277 121 9	2.974 649 2	0.239 084 0				
H	−5.056 569 8	3.872 255 2	0.489 761 1				
N	−2.875 774 2	3.259 774 3	−0.039 499 1				
B	−2.417 994 6	4.614 627 0	0.239 030 9				
H	−3.211 306 5	5.499 917 1	0.489 883 5				
N	−1.017 447 3	4.884 395 6	0.259 074 8				
H	−0.710 024 4	5.817 574 7	0.520 457 7				
B	−0.040 115 3	3.865 961 0	−0.049 036 4				
N	1.378 689 0	4.100 896 1	0.080 680 9				
H	1.686 730 5	5.016 965 9	0.397 579 3				
B	2.367 315 3	3.056 671 4	−0.050 185 8				
N	3.761 748 4	3.277 668 6	0.256 328 4				
H	4.080 804 5	4.207 035 0	0.517 321 4				
B	4.714 800 8	2.216 548 5	0.235 151 4				
H	5.882 004 7	2.442 689 3	0.485 270 0				
N	4.260 897 4	0.860 449 8	−0.043 587 6				
B	2.866 357 5	0.579 091 6	−0.441 168 2				
N	1.943 611 0	1.743 724 4	−0.548 024 5				
B	0.573 149 5	1.706 833 4	−1.084 346 3				
N	−0.496 119 1	2.564 039 1	−0.546 986 0				
B	−1.934 777 2	2.193 274 0	−0.438 260 6				
N	−2.482 070 4	0.811 976 6	−0.546 102 4				
B	−3.830 838 4	0.521 951 2	−0.048 397 6				
N	−4.719 487 0	1.618 680 3	0.259 447 5				
H	−5.683 798 5	1.430 037 9	0.520 566 7				
N	−4.240 843 8	−0.856 458 2	0.081 016 2				
H	−5.188 266 9	−1.048 056 5	0.397 574 5				
B	−3.328 101 8	−1.967 601 5	−0.050 135 5				
N	−1.972 475 1	−1.710 960 9	−0.547 503 4				
B	−1.764 364 0	−0.355 740 5	−1.082 900 7				
Al	−0.000 790 1	0.000 863 5	−2.109 490 3				
B	1.190 274 8	−1.349 477 6	−1.085 661 2				
N	2.467 899 9	−0.852 184 0	−0.549 549 4				
B	3.368 078 0	−1.898 287 9	−0.053 324 9				
N	4.739 099 3	−1.561 326 1	0.253 334 4				
B	5.205 652 0	−0.213 491 0	0.233 675 2				
H	6.369 150 4	0.030 877 7	0.483 828 2				
H	5.394 040 7	−2.294 245 6	0.513 454 1				
N	2.862 355 8	−3.244 546 5	0.076 180 7				
H	3.502 253 5	−3.969 327 3	0.391 871 5				
B	1.463 491 5	−3.578 479 3	−0.052 948 7				
N	0.537 711 7	−2.554 981 2	−0.549 286 9				
B	−0.932 076 3	−2.771 703 0	−0.440 586 0				
N	−1.385 204 0	−4.120 177 8	−0.042 958 2				
B	−2.787 412 4	−4.401 513 0	0.235 132 5				
N	−3.721 513 3	−3.323 646 1	0.256 036 0				
H	−4.683 505 8	−3.524 549 1	0.516 547 6				
H	−3.157 307 2	−5.531 500 2	0.484 882 9				
B	−0.437 462 9	−5.191 424 0	0.234 302 0				
H	−0.825 034 1	−6.315 430 9	0.484 235 3				
N	0.958 031 4	−4.896 641 6	0.254 050 0				
H	1.603 654 7	−5.637 678 6	0.514 181 1				

Table TS34: B97-D3/SVP optimized geometry of the C_B

At.	X	Y	Z	At.	X	Y	Z
N	−4.955 963 9	1.591 805 0	0.002 348 9				
B	−3.699 578 0	2.307 230 3	−0.003 708 7				
N	−3.609 916 8	3.750 311 0	0.002 556 4				
B	−2.358 891 2	4.442 319 5	0.005 504 3				
N	−1.122 244 4	3.668 658 0	−0.001 924 7				
B	−1.178 973 7	2.207 662 7	−0.012 067 6				
N	0.048 065 5	1.411 759 6	−0.020 767 5				
C	0.000 009 2	0.000 034 6	−0.049 548 9				
N	1.198 526 1	−0.747 469 7	−0.020 566 4				
B	2.501 314 3	−0.082 735 9	−0.011 493 5				
N	2.563 964 7	1.366 151 3	−0.013 041 6				
B	1.326 458 9	2.122 304 7	−0.011 696 0				
N	1.369 272 2	3.583 805 9	−0.001 576 5				
B	2.655 666 0	4.271 626 9	0.006 008 1				
N	3.856 733 8	3.496 148 1	0.003 288 5				
B	3.847 964 7	2.050 336 0	−0.002 871 5				
N	5.052 801 3	1.251 078 7	0.003 406 1				
B	5.026 565 3	−0.178 212 0	0.006 409 0				
N	3.738 207 1	−0.862 382 1	−0.001 119 1				
B	3.700 798 1	−2.308 029 3	0.002 891 0				
N	2.419 033 6	−2.977 647 8	−0.001 300 1				
B	2.371 481 1	−4.435 567 0	0.006 231 6				
N	1.099 375 7	−5.088 093 8	0.003 194 4				
B	−0.148 465 4	−4.357 547 0	−0.003 078 2				
N	−0.098 858 3	−2.903 488 3	−0.013 312 9				
B	−1.322 378 5	−2.124 831 0	−0.011 863 5				
N	−2.615 964 1	−2.806 264 2	−0.001 693 5				
B	−3.849 261 5	−2.051 068 6	0.002 014 3				
N	−3.788 266 0	−0.606 176 6	−0.002 053 1				
B	−5.027 135 4	0.164 029 3	0.005 253 8				
H	−6.102 742 2	−0.397 417 0	0.014 413 8				
B	−2.501 178 1	0.087 502 9	−0.012 126 6				
N	−2.465 067 6	1.537 335 7	−0.013 730 0				
N	−1.246 640 9	−0.664 232 1	−0.020 842 7				
H	−4.918 500 6	−2.620 869 6	0.009 402 5				
B	−2.667 686 7	−4.264 165 8	0.005 985 4				
N	−1.442 871 7	−5.001 329 8	0.003 202 8				
H	−1.497 053 2	−6.016 698 6	0.010 438 8				
H	−3.733 631 5	−4.843 754 3	0.015 351 7				
B	1.174 789 4	−2.209 824 1	−0.011 681 2				
H	1.084 323 9	−6.104 365 7	0.010 373 0				
H	3.395 520 8	−5.086 368 6	0.015 745 5				
H	4.728 889 8	−2.949 151 6	0.010 675 0				
H	6.061 469 8	−0.811 582 4	0.015 835 7				
H	5.959 046 3	1.711 846 0	0.010 464 5				
H	4.744 551 2	3.991 343 0	0.010 390 5				
H	2.707 196 0	5.483 841 4	0.015 243 7				
B	0.148 469 8	4.359 047 8	0.002 101 9				
H	0.189 649 8	5.569 985 7	0.009 466 7				
H	−2.328 045 9	5.655 271 3	0.014 836 1				
H	−4.461 947 1	4.304 693 5	0.009 677 8				
H	−5.828 881 5	2.113 144 0	0.009 351 8				

Table TS35: B97-D3/SVP optimized geometry of the C_BC_N

At.	X	Y	Z	At.	X	Y	Z
N	5.098 199 1	−2.490 254 9	0.015 566 1				
B	3.650 532 1	−2.533 671 5	0.008 775 7				
N	2.921 499 4	−1.273 795 6	0.001 508 2				
B	1.460 586 6	−1.297 293 4	−0.004 787 2				
N	0.734 110 0	−2.558 583 1	−0.003 008 5				
B	−0.705 085 1	−2.512 139 5	−0.005 574 2				
N	−1.439 039 3	−1.217 440 1	−0.010 549 7				
B	−2.903 081 9	−1.246 309 6	−0.014 533 2				
N	−3.623 856 1	−2.515 367 0	−0.014 422 3				
B	−5.081 793 9	−2.521 011 1	−0.019 061 0				
N	−5.779 274 4	−1.271 634 6	−0.023 754 7				
H	−6.795 775 8	−1.293 167 7	−0.026 294 8				
B	−5.096 224 6	−0.000 011 7	−0.022 376 0				
N	−3.636 488 6	−0.000 004 4	−0.018 231 8				
B	−2.903 085 0	1.246 303 2	−0.014 557 5				
N	−3.623 865 4	2.515 358 3	−0.014 481 5				
B	−5.081 801 1	2.520 994 5	−0.019 155 0				
N	−5.779 293 1	1.271 617 3	−0.023 822 8				
H	−6.795 775 0	1.293 149 3	−0.026 370 2				
H	−5.696 831 1	3.566 496 1	−0.018 750 1				
B	−2.903 260 3	3.773 409 1	−0.007 391 6				
H	−3.514 523 3	4.819 890 4	−0.006 058 5				
N	−1.460 193 9	3.769 046 1	−0.001 874 9				
B	−0.741 041 6	5.042 817 5	0.008 123 1				
H	−1.353 900 0	6.090 684 0	0.013 036 9				
N	0.687 985 1	5.044 160 1	0.012 115 3				
H	1.161 511 6	5.943 907 1	0.019 238 8				
B	1.464 241 4	3.820 602 5	0.006 412 6				
N	0.734 105 1	2.558 588 0	−0.002 878 9				
B	−0.705 087 0	2.512 145 4	−0.005 507 3				
N	−1.439 042 3	1.217 442 6	−0.010 544 1				
C	−0.716 891 5	0.000 002 2	−0.010 701 2				
C	0.666 566 4	0.000 004 8	−0.009 632 2				
B	1.460 582 3	1.297 304 9	−0.004 711 4				
N	2.921 495 6	1.273 805 6	0.001 625 5				
B	3.650 528 1	2.533 679 3	0.009 076 4				
N	2.903 932 9	3.777 119 9	0.010 613 9				
H	3.420 150 9	4.653 408 0	0.018 383 3				
N	5.098 201 5	2.490 264 7	0.015 909 8				
H	5.626 778 9	3.358 688 6	0.020 063 0				
B	5.834 751 2	1.261 169 2	0.018 885 1				
N	5.110 285 3	0.000 004 4	0.014 019 9				
B	3.638 950 2	0.000 005 7	0.005 117 2				
B	5.834 745 6	−1.261 164 9	0.018 714 3				
H	7.050 276 6	−1.280 485 8	0.025 389 8				
H	7.050 284 6	1.280 500 4	0.025 551 9				
H	−5.696 817 2	−3.566 514 4	−0.018 618 8				
B	−2.903 252 4	−3.773 414 7	−0.007 407 7				
N	−1.460 187 7	−3.769 043 2	−0.001 964 4				
B	−0.741 037 9	−5.042 808 7	0.007 905 5				
H	−1.353 889 6	−6.090 675 5	0.012 791 0				
N	0.687 995 3	−5.044 167 1	0.011 790 9				
B	1.464 245 7	−3.820 596 6	0.006 117 7				
N	2.903 934 4	−3.777 115 8	0.010 186 7				
H	3.420 153 3	−4.653 401 7	0.017 791 4				
H	1.161 492 2	−5.943 915 7	0.018 813 5				
H	−3.514 521 5	−4.819 893 9	−0.006 097 2				
H	5.626 795 4	−3.358 686 8	0.019 595 0				

Table TS36: B97-D3/SVP optimized geometry of the C_N

At.	X	Y	Z	At.	X	Y	Z
B	−5.157 741 5	−1.106 897 5	−0.001 685 4				
N	−3.950 678 7	−1.925 151 1	0.003 854 5				
B	−2.631 642 3	−1.282 423 6	0.008 339 9				
N	−2.559 561 3	0.185 923 1	0.009 358 1				
B	−3.765 405 3	1.003 044 0	0.002 169 9				
N	−5.044 408 0	0.321 896 7	−0.002 674 8				
H	−5.905 299 7	0.862 974 1	−0.008 687 7				
N	−3.621 861 1	2.444 766 8	−0.001 105 7				
H	−4.465 317 0	3.013 829 2	−0.007 140 0				
B	−2.338 520 9	3.116 998 4	0.001 724 1				
N	−2.184 916 3	4.557 815 1	−0.003 642 3				
H	−3.008 614 4	5.153 874 7	−0.009 836 7				
B	−0.902 130 0	5.197 215 9	−0.002 891 9				
H	−0.829 052 5	6.410 322 9	−0.007 557 2				
N	0.308 114 7	4.383 693 4	0.002 893 2				
B	0.205 311 6	2.920 110 3	0.007 764 1				
N	−1.129 656 7	2.304 178 5	0.009 003 1				
B	−1.264 918 3	0.853 664 3	0.011 760 0				
C	0.000 044 0	0.000 026 7	0.014 396 6				
B	−0.106 961 6	−1.522 178 7	0.011 823 1				
N	−1.430 692 3	−2.130 422 6	0.009 512 6				
B	−1.530 279 5	−3.583 602 2	0.002 567 6				
N	−2.854 938 7	−4.171 185 6	−0.002 123 1				
B	−4.050 088 0	−3.379 967 7	−0.001 370 3				
H	−5.137 197 2	−3.923 289 4	−0.005 601 0				
H	−2.959 338 5	−5.182 584 0	−0.007 913 1				
N	−0.306 364 7	−4.359 068 5	−0.000 659 2				
H	−0.377 897 2	−5.374 013 1	−0.006 481 7				
B	1.013 942 7	−3.762 478 5	0.001 872 3				
N	1.118 748 9	−2.309 584 7	0.009 020 5				
B	2.426 471 9	−1.637 740 3	0.007 610 0				
N	2.560 466 1	−0.173 825 7	0.009 011 6				
B	1.371 962 9	0.668 505 1	0.011 674 2				
N	1.440 890 4	2.123 598 3	0.009 133 3				
B	2.751 549 6	2.759 366 5	0.002 294 7				
N	2.801 070 3	4.207 583 1	−0.002 703 5				
B	1.620 293 2	5.020 056 4	−0.002 353 2				
H	1.717 484 5	6.231 459 4	−0.006 922 7				
H	3.700 019 1	4.682 579 0	−0.008 427 5				
N	3.928 365 5	1.914 285 3	−0.000 530 8				
H	4.842 867 9	2.360 061 7	−0.006 106 9				
B	3.868 779 2	0.466 701 7	0.002 047 8				
N	5.039 918 8	−0.386 646 6	−0.003 307 3				
B	4.952 239 4	−1.817 247 9	−0.002 975 9				
N	3.642 497 4	−2.458 659 8	0.002 554 1				
B	3.537 417 0	−3.913 076 2	−0.003 172 2				
N	2.243 343 2	−4.529 382 4	−0.003 662 1				
H	2.205 287 9	−5.545 435 9	−0.009 819 6				
H	4.537 783 6	−4.603 140 5	−0.008 000 8				
H	5.966 305 9	−2.487 045 1	−0.007 778 1				
H	5.967 875 1	0.028 753 4	−0.009 280 6				
H	−6.255 568 7	−1.628 236 3	−0.005 974 0				

Table TS37: B97-D3/SVP optimized geometry of the Mg_B

At.	X	Y	Z	At.	X	Y	Z
N	1.313 811 3	−4.994 487 3	−0.046 285 1				
B	2.069 028 7	−3.771 490 9	0.128 395 5				
N	3.506 829 2	−3.786 674 1	−0.037 926 1				
B	4.299 209 6	−2.601 306 8	−0.135 675 8				
N	3.640 206 3	−1.328 777 9	0.074 574 3				
B	2.219 453 4	−1.275 224 7	0.501 316 4				
N	1.741 757 4	0.003 799 6	0.954 518 0				
Mg	−0.017 434 5	−0.012 799 1	1.800 573 1				
N	−0.804 301 6	1.440 818 4	0.687 948 4				
B	0.061 445 8	2.548 815 4	0.349 604 4				
N	1.530 949 6	2.483 885 3	0.333 997 5				
B	2.316 269 5	1.221 049 3	0.467 149 9				
N	3.733 648 4	1.202 409 9	0.062 998 7				
B	4.444 283 8	2.439 909 0	−0.192 993 4				
N	3.704 701 5	3.664 962 9	−0.129 453 1				
B	2.267 682 4	3.713 538 5	0.041 182 9				
N	1.542 041 5	4.961 878 5	−0.101 720 7				
B	0.117 151 5	5.041 058 0	−0.129 717 9				
N	−0.644 711 4	3.814 689 3	0.054 361 4				
B	−2.091 691 1	3.813 539 7	−0.092 340 2				
N	−2.873 185 1	2.590 720 0	0.007 438 9				
B	−4.309 311 8	2.607 511 5	−0.227 973 3				
N	−5.006 345 8	1.361 784 8	−0.243 570 7				
B	−4.347 391 7	0.078 475 4	−0.091 253 1				
N	−2.924 300 1	0.037 508 7	0.245 437 0				
B	−2.283 707 6	−1.301 450 4	0.386 485 1				
N	−3.018 415 4	−2.508 070 6	−0.031 923 1				
B	−2.273 985 4	−3.753 124 1	−0.151 343 9				
N	−0.833 825 1	−3.789 670 7	0.033 938 0				
B	−0.109 882 8	−5.029 586 0	−0.159 849 7				
H	−0.684 899 6	−6.068 892 0	−0.414 147 9				
B	−0.122 215 3	−2.561 636 8	0.468 295 3				
N	1.374 207 0	−2.512 840 6	0.445 499 8				
N	−0.957 625 1	−1.471 195 8	0.900 665 2				
H	−2.864 882 6	−4.771 259 3	−0.450 028 5				
B	−4.435 734 8	−2.441 777 6	−0.331 685 2				
N	−5.071 680 8	−1.159 195 2	−0.292 489 0				
H	−6.062 264 0	−1.114 825 0	−0.517 900 0				
H	−5.071 977 0	−3.437 789 8	−0.611 834 2				
B	−2.191 956 7	1.312 402 0	0.304 347 5				
H	−6.001 726 6	1.378 286 3	−0.451 467 9				
H	−4.899 622 7	3.651 180 5	−0.420 002 5				
H	−2.659 813 7	4.857 186 6	−0.340 074 0				
H	−0.441 926 0	6.102 778 0	−0.316 324 9				
H	2.067 350 5	5.815 107 1	−0.275 952 1				
H	4.203 157 2	4.529 790 3	−0.323 668 9				
H	5.630 225 5	2.448 344 7	−0.455 679 2				
B	4.385 065 3	−0.091 177 9	−0.078 765 8				
H	5.564 013 8	−0.133 943 7	−0.367 737 8				
H	5.485 424 4	−2.670 254 6	−0.388 372 7				
H	3.963 265 7	−4.677 073 0	−0.219 755 9				
H	1.823 634 9	−5.856 906 2	−0.220 815 1				

Table TS38: B97-D3/SVP optimized geometry of the Mg_N

At.	X	Y	Z	At.	X	Y	Z
B	−1.520 28	−4.680 65	−1.102 26				
H	−2.117 65	−5.686 88	−1.422 62				
N	−2.206 03	−3.541 38	−0.528 42				
B	−3.613 63	−3.619 84	−0.191 69				
H	−4.251 54	−4.629 35	−0.407 98				
N	−4.249 65	−2.477 96	0.391 12				
H	−5.250 31	−2.526 16	0.559 45				
B	−3.549 16	−1.236 00	0.614 45				
N	−4.206 52	0.004 85	0.941 70				
H	−5.217 04	0.006 75	1.049 23				
B	−3.544 07	1.244 80	0.621 17				
N	−4.240 58	2.490 32	0.405 50				
H	−5.240 66	2.541 26	0.576 40				
B	−3.602 14	3.632 01	−0.174 75				
H	−4.236 93	4.644 65	−0.385 42				
N	−2.195 96	3.549 34	−0.516 35				
B	−1.441 73	2.325 26	−0.227 71				
N	−2.095 84	1.230 93	0.440 79				
B	−1.378 64	0.000 26	0.709 93				
N	−2.100 97	−1.226 25	0.435 79				
B	−1.448 84	−2.320 61	−0.234 14				
N	−0.041 95	−2.174 67	−0.567 17				
B	0.665 71	−3.440 02	−0.825 01				
N	−0.103 48	−4.596 82	−1.237 20				
H	0.394 34	−5.429 97	−1.537 39				
N	2.085 46	−3.556 71	−0.607 80				
H	2.530 70	−4.457 84	−0.753 96				
B	2.832 60	−2.451 32	−0.087 22				
N	2.135 24	−1.192 13	0.030 07				
B	0.727 28	−0.930 77	−0.336 12				
Mg	0.303 56	−0.014 01	2.586 44				
B	0.731 47	0.931 30	−0.340 05				
N	−0.036 34	2.176 45	−0.566 47				
B	0.673 93	3.439 80	−0.828 07				
N	−0.093 89	4.599 30	−1.234 87				
B	−1.509 56	4.687 39	−1.091 78				
H	−2.105 44	5.696 06	−1.407 19				
H	0.404 84	5.431 27	−1.536 82				
N	2.095 44	3.552 08	−0.619 88				
H	2.542 27	4.452 04	−0.768 33				
B	2.841 50	2.445 43	−0.100 39				
N	2.140 78	1.188 67	0.020 58				
B	2.915 20	−0.002 65	0.208 98				
N	4.339 22	−0.004 38	0.482 45				
B	5.006 56	−1.294 34	0.546 29				
N	4.242 45	−2.475 36	0.260 92				
H	4.740 91	−3.360 85	0.247 83				
H	6.189 91	−1.375 93	0.803 99				
B	5.011 59	1.283 42	0.537 12				
H	6.195 46	1.361 94	0.793 42				
N	4.252 01	2.465 78	0.245 07				
H	4.753 80	3.349 31	0.227 54				

Table TS39: B97-D3/SVP optimized geometry of the P_B

At.	X	Y	Z	At.	X	Y	Z
N	−1.415 544 5	5.012 696 1	0.063 610 0				
B	−2.172 066 5	3.785 210 1	−0.054 898 9				
N	−3.613 516 3	3.751 334 1	0.064 131 9				
B	−4.362 238 9	2.534 396 0	0.134 112 4				
N	−3.649 206 6	1.270 534 8	0.006 958 1				
B	−2.208 642 0	1.275 384 9	−0.252 433 5				
N	−1.546 236 1	0.003 878 0	−0.485 326 9				
P	−0.000 241 8	−0.000 526 3	−1.315 965 1				
N	0.769 754 6	−1.341 643 3	−0.485 538 2				
B	−0.000 268 1	−2.550 944 3	−0.251 757 3				
N	−1.465 231 7	−2.522 573 3	−0.259 537 3				
B	−2.215 471 0	−1.264 012 3	−0.252 196 9				
N	−3.656 062 7	−1.251 430 4	0.006 825 0				
B	−4.375 914 7	−2.511 446 8	0.133 646 0				
N	−3.633 568 1	−3.732 313 2	0.064 001 6				
B	−2.192 191 4	−3.773 853 0	−0.054 031 2				
N	−1.442 123 7	−5.005 207 7	0.065 517 1				
B	−0.013 897 3	−5.045 155 4	0.136 549 7				
N	0.724 180 0	−3.795 828 5	0.008 667 5				
B	2.172 992 3	−3.786 710 6	0.102 192 3				
N	2.911 673 2	−2.540 396 6	0.008 756 1				
B	4.362 830 3	−2.533 573 2	0.136 116 4				
N	5.048 870 9	−1.280 309 2	0.065 301 6				
B	4.364 175 7	−0.011 464 1	−0.054 356 4				
N	2.916 997 9	−0.007 733 5	−0.259 753 7				
B	2.209 129 1	1.275 102 6	−0.253 143 5				
N	2.925 220 9	2.525 227 0	0.005 561 5				
B	2.193 187 9	3.775 492 8	0.098 160 1				
N	0.744 366 1	3.791 962 8	0.005 679 0				
B	0.012 905 9	5.045 142 9	0.133 482 5				
H	0.598 818 6	6.094 702 0	0.299 158 5				
B	0.013 258 0	2.550 714 4	−0.253 454 3				
N	−1.451 821 7	2.530 024 9	−0.260 401 1				
N	0.776 824 6	1.337 232 4	−0.486 741 3				
H	2.796 379 3	4.813 593 6	0.267 988 4				
B	4.376 351 2	2.510 869 1	0.132 445 2				
N	5.055 670 9	1.253 875 9	0.063 182 2				
H	6.065 804 6	1.254 595 6	0.179 915 0				
H	4.997 922 3	3.540 011 3	0.296 742 0				
B	2.202 295 8	−1.286 792 1	−0.251 658 6				
H	6.058 934 4	−1.286 254 1	0.182 345 1				
H	4.978 933 8	−3.565 754 1	0.301 990 5				
H	2.770 543 7	−4.827 799 0	0.273 535 8				
H	0.566 285 5	−6.097 799 7	0.303 176 3				
H	−1.946 527 4	−5.880 312 4	0.182 864 9				
H	−4.144 015 1	−4.603 998 7	0.180 782 1				
H	−5.578 005 6	−2.528 939 3	0.298 201 2				
B	−4.366 139 1	0.011 474 1	0.099 420 0				
H	−5.566 811 9	0.014 711 9	0.269 050 8				
H	−5.564 102 6	2.558 337 6	0.299 188 2				
H	−4.119 242 6	4.625 740 7	0.181 130 4				
H	−1.915 220 2	5.890 525 6	0.180 809 0				

Table TS40: B97-D3/SVP optimized geometry of the P_N

At.	X	Y	Z	At.	X	Y	Z
B	5.072 985 3	1.307 434 9	0.181 768 3				
H	6.273 176 9	1.326 287 5	0.368 697 3				
N	4.368 157 2	0.046 864 2	−0.002 816 8				
B	5.099 881 3	−1.198 369 3	0.181 458 3				
H	6.300 222 3	−1.191 522 6	0.368 363 0				
N	4.382 496 6	−2.434 780 9	0.168 840 4				
H	4.904 218 0	−3.290 362 3	0.340 292 7				
B	2.954 019 7	−2.501 841 3	−0.046 420 9				
N	2.211 739 5	−3.737 701 9	0.027 763 2				
H	2.719 300 8	−4.594 923 3	0.232 466 4				
B	0.771 179 6	−3.793 703 7	−0.047 288 7				
N	0.024 758 0	−5.013 631 6	0.167 054 7				
H	0.523 573 9	−5.882 891 3	0.337 941 4				
B	−1.404 328 8	−5.047 294 9	0.179 568 3				
H	−1.988 181 3	−6.096 274 4	0.365 855 2				
N	−2.143 584 1	−3.806 386 7	−0.004 158 3				
B	−1.434 888 8	−2.547 966 7	−0.278 281 5				
N	0.034 879 6	−2.568 955 7	−0.361 023 8				
B	0.825 464 4	−1.394 611 7	−0.711 543 8				
N	2.235 099 2	−1.266 940 2	−0.360 371 2				
B	2.924 117 2	0.031 424 7	−0.277 407 9				
N	2.207 420 6	1.314 739 8	−0.360 192 9				
B	2.899 706 7	2.564 654 1	−0.045 926 2				
N	4.329 291 4	2.528 238 5	0.169 370 2				
H	4.832 626 6	3.394 709 3	0.341 062 9				
N	2.130 996 5	3.784 199 7	0.028 562 7				
H	2.619 922 1	4.652 078 4	0.233 439 9				
B	0.689 603 9	3.809 347 8	−0.046 446 8				
N	−0.020 245 4	2.569 296 1	−0.360 496 4				
B	0.795 379 1	1.412 413 6	−0.711 337 1				
P	0.000 364 9	0.000 324 7	−1.701 764 2				
B	−1.620 408 3	−0.017 127 8	−0.711 756 8				
N	−2.214 710 1	−1.301 916 3	−0.361 000 5				
B	−3.643 687 3	−1.307 232 4	−0.046 765 7				
N	−4.299 818 4	−2.577 921 2	0.168 397 2				
B	−3.587 910 2	−3.817 507 1	0.180 362 6				
H	−4.182 072 8	−4.860 673 7	0.366 878 7				
H	−5.301 575 2	−2.601 955 3	0.340 068 1				
N	−4.342 802 2	−0.046 552 2	0.027 836 4				
H	−5.338 757 5	−0.057 247 5	0.233 400 3				
B	−3.670 814 6	1.228 836 8	−0.046 564 4				
N	−2.242 097 2	1.254 440 0	−0.360 685 4				
B	−1.489 164 8	2.516 821 0	−0.277 676 3				
N	−2.224 705 7	3.759 526 1	−0.003 207 8				
B	−1.512 194 8	5.015 798 5	0.180 814 2				
N	−0.082 756 3	5.012 915 8	0.168 206 5				
H	0.397 107 6	5.892 660 4	0.339 252 6				
H	−2.118 534 6	6.051 763 7	0.367 415 2				
B	−3.668 893 2	3.739 521 3	0.181 162 7				
H	−4.285 382 0	4.769 519 8	0.367 946 4				
N	−4.354 056 8	2.485 041 2	0.168 712 3				
H	−5.356 118 5	2.487 434 3	0.340 174 8				

Table TS41: B97-D3/SVP optimized geometry of the Si_B

At.	X	Y	Z	At.	X	Y	Z
N	1.119 658 2	5.084 899 6	0.104 983 1				
B	−0.125 323 0	4.358 654 1	−0.032 738 6				
N	−1.409 902 2	5.012 382 2	0.104 297 4				
B	−2.653 645 1	4.306 232 3	0.148 659 3				
N	−2.632 930 2	2.861 570 9	−0.028 276 7				
B	−1.365 482 2	2.186 604 8	−0.315 058 1				
N	−1.415 978 1	0.764 146 2	−0.597 121 3				
Si	0.000 210 8	−0.000 193 6	−1.272 093 6				
N	0.046 232 2	−1.608 157 6	−0.595 828 0				
B	−1.210 855 1	−2.275 652 2	−0.313 793 2				
N	−2.483 874 1	−1.530 778 3	−0.283 164 3				
B	−2.577 368 4	−0.058 809 7	−0.315 027 5				
N	−3.837 244 7	0.630 184 8	−0.028 150 3				
B	−5.056 173 2	−0.145 493 6	0.148 700 1				
N	−4.963 693 5	−1.572 765 9	0.103 967 7				
B	−3.712 061 2	−2.287 725 7	−0.032 593 1				
N	−3.635 845 8	−3.726 983 5	0.105 625 3				
B	−2.402 351 0	−4.450 896 3	0.151 037 6				
N	−1.161 610 3	−3.710 654 6	−0.026 047 9				
B	0.125 959 7	−4.381 704 3	0.072 161 2				
N	1.372 918 5	−3.637 962 4	−0.026 496 7				
B	2.654 161 2	−4.305 822 3	0.150 032 9				
N	3.844 108 3	−3.512 352 8	0.104 289 4				
B	3.837 446 1	−2.070 995 5	−0.032 979 8				
N	2.567 735 5	−1.385 768 0	−0.283 183 5				
B	2.576 387 9	0.089 157 1	−0.314 432 2				
N	3.794 521 5	0.849 200 2	−0.027 251 6				
B	3.731 659 7	2.299 744 3	0.071 136 1				
N	2.464 065 3	3.007 759 8	−0.026 899 7				
B	2.401 849 3	4.451 172 3	0.150 051 4				
H	3.407 661 6	5.104 186 5	0.336 443 3				
B	1.237 592 4	2.261 295 1	−0.314 226 3				
N	−0.083 832 5	2.916 482 5	−0.283 391 8				
N	1.369 720 2	0.844 122 4	−0.596 257 1				
H	4.751 410 6	2.928 295 9	0.261 774 2				
B	5.055 984 8	0.144 828 0	0.149 500 1				
N	5.045 870 8	−1.285 375 0	0.104 201 4				
H	5.928 727 9	−1.767 779 0	0.252 800 0				
H	6.091 345 6	0.749 846 2	0.335 935 1				
B	1.339 596 1	−2.202 534 1	−0.314 003 6				
H	4.732 078 6	−3.985 442 4	0.252 638 5				
H	2.716 920 1	−5.503 331 4	0.336 537 4				
H	0.160 551 0	−5.579 141 0	0.263 071 2				
H	−2.396 131 0	−5.649 973 2	0.337 894 0				
H	−4.495 092 3	−4.250 250 6	0.254 617 5				
H	−5.817 315 7	−2.105 224 7	0.252 508 8				
H	−6.124 776 4	0.398 870 6	0.334 685 2				
B	−3.857 830 7	2.082 027 6	0.069 758 5				
H	−4.912 049 6	2.651 094 6	0.260 014 4				
H	−3.695 324 8	4.900 556 3	0.334 541 1				
H	−1.433 257 2	6.018 089 2	0.253 393 1				
H	1.085 574 6	6.090 364 2	0.253 759 5				

Table TS42: B97-D3/SVP optimized geometry of the Si_N

At.	X	Y	Z	At.	X	Y	Z
B	−3.335 694 5	4.022 672 0	0.212 153 2				
H	−3.853 438 4	5.098 022 0	0.438 912 9				
N	−1.901 369 4	3.922 132 9	−0.023 469 7				
B	−1.091 710 5	5.110 291 8	0.212 255 3				
H	−1.614 996 8	6.183 000 0	0.438 818 7				
N	0.331 968 9	4.994 950 5	0.211 628 8				
H	0.879 221 8	5.822 833 7	0.432 375 5				
B	1.002 466 3	3.742 713 1	−0.055 960 7				
N	2.435 200 3	3.591 525 7	0.048 390 9				
H	2.986 109 0	4.404 444 2	0.313 661 7				
B	3.106 030 0	2.316 524 5	−0.055 567 6				
N	4.517 414 7	2.157 261 1	0.212 189 8				
H	5.083 728 4	2.972 252 7	0.432 447 9				
B	5.151 422 4	0.877 445 2	0.213 556 6				
H	6.341 521 8	0.788 285 9	0.440 365 8				
N	4.347 408 5	−0.314 647 3	−0.021 584 0				
B	2.919 395 9	−0.211 522 9	−0.367 283 0				
N	2.329 752 5	1.144 918 1	−0.466 897 4				
B	0.958 681 4	1.413 399 7	−0.904 455 6				
N	0.201 683 4	2.587 739 9	−0.467 343 2				
B	−1.276 688 4	2.633 490 7	−0.368 535 2				
N	−2.156 834 9	1.444 833 4	−0.467 928 4				
B	−3.559 459 3	1.531 694 7	−0.056 429 9				
N	−4.127 082 9	2.833 720 1	0.211 289 0				
H	−5.115 950 0	2.916 678 3	0.432 542 1				
N	−4.328 375 1	0.313 323 8	0.048 249 7				
H	−5.307 630 9	0.384 181 2	0.313 868 6				
B	−3.742 898 4	−1.003 172 6	−0.055 133 9				
N	−2.342 218 9	−1.119 531 4	−0.466 434 6				
B	−1.703 990 7	0.122 948 6	−0.904 737 7				
Si	0.000 082 2	−0.000 794 4	−1.893 187 7				
B	0.745 401 8	−1.537 737 3	−0.903 939 3				
N	2.140 730 1	−1.468 980 8	−0.466 298 7				
B	2.740 287 3	−2.739 668 4	−0.053 770 1				
N	4.159 818 3	−2.785 089 0	0.214 576 9				
B	4.971 442 2	−1.609 802 4	0.215 133 9				
H	6.161 931 5	−1.692 613 2	0.442 411 1				
H	4.603 084 6	−3.672 776 9	0.436 246 9				
N	1.892 892 0	−3.904 727 6	0.051 130 6				
H	2.321 175 0	−4.788 078 2	0.317 275 0				
B	0.453 347 1	−3.848 116 6	−0.053 398 3				
N	−0.172 949 7	−2.590 281 9	−0.465 766 7				
B	−1.642 486 8	−2.422 540 7	−0.366 565 9				
N	−2.445 887 4	−3.607 470 8	−0.020 456 9				
B	−3.879 629 4	−3.500 232 2	0.215 251 0				
N	−4.491 841 8	−2.209 832 2	0.213 347 7				
H	−5.482 519 9	−2.149 990 8	0.433 718 7				
H	−4.546 707 6	−4.489 660 9	0.442 813 8				
B	−1.815 655 2	−4.899 609 5	0.215 999 3				
H	−2.487 998 8	−5.885 471 0	0.443 479 5				
N	−0.390 332 1	−4.990 519 8	0.214 973 4				
H	0.032 150 7	−5.888 414 8	0.436 080 3				

Table TS43: B97-D3/SVP optimized geometry of the V_B

At.	X	Y	Z	At.	X	Y	Z
N	−1.273 638 7	5.047 936 2	0.025 968 7				
B	−0.001 957 2	4.358 912 9	0.019 202 8				
N	1.269 122 9	5.049 026 0	0.027 358 8				
B	2.530 047 3	4.371 709 0	0.014 997 5				
N	2.558 373 7	2.914 427 9	−0.002 970 1				
B	1.258 677 3	2.184 474 0	−0.009 303 6				
N	1.424 846 1	0.779 887 5	−0.031 814 4				
N	0.000 718 4	−1.536 991 4	−0.007 254 0				
B	1.290 097 6	−2.141 532 8	−0.008 812 2				
N	2.566 223 7	−1.460 490 2	−0.010 587 1				
B	2.582 506 3	−0.013 990 6	−0.018 541 6				
N	3.856 329 5	0.743 964 1	−0.015 175 8				
B	5.103 401 3	−0.007 103 8	−0.009 541 1				
N	5.056 044 3	−1.440 217 4	−0.004 558 9				
B	3.820 437 3	−2.194 055 4	−0.003 972 1				
N	3.761 081 9	−3.638 715 3	0.002 868 4				
B	2.525 598 2	−4.358 616 5	0.001 451 8				
N	1.260 329 5	−3.630 804 8	−0.005 446 2				
B	0.001 938 8	−4.345 968 1	−0.005 404 4				
N	−1.257 064 8	−3.631 877 5	−0.003 462 7				
B	−2.521 676 2	−4.360 802 8	0.005 622 1				
N	−3.757 784 5	−3.641 983 4	0.008 733 4				
B	−3.818 431 5	−2.197 383 9	0.000 985 3				
N	−2.564 881 3	−1.462 702 4	−0.007 780 9				
B	−2.582 502 7	−0.016 223 3	−0.017 753 8				
N	−3.857 016 1	0.740 572 4	−0.013 548 1				
B	−3.822 917 2	2.192 156 0	−0.009 244 9				
N	−2.561 024 7	2.912 218 9	−0.003 907 8				
B	−2.533 978 4	4.369 531 4	0.013 320 4				
H	−3.571 178 0	5.000 823 3	0.018 340 0				
B	−1.260 696 2	2.183 407 2	−0.010 312 6				
N	−0.001 320 0	2.902 504 2	0.001 291 8				
N	−1.425 597 1	0.778 691 3	−0.034 465 7				
H	−4.865 325 0	2.812 161 7	−0.007 310 2				
B	−5.103 397 8	−0.011 610 3	−0.005 349 7				
N	−5.054 720 1	−1.444 671 4	0.001 281 1				
H	−5.939 023 6	−1.946 539 1	0.007 356 0				
H	−6.170 660 5	0.567 472 3	−0.002 672 0				
B	−1.288 146 5	−2.142 624 6	−0.007 144 8				
H	−4.620 176 8	−4.180 389 1	0.015 112 0				
H	−2.519 084 1	−5.574 843 7	0.011 270 1				
H	0.002 452 3	−5.558 334 0	−0.006 711 5				
H	2.524 079 5	−5.572 659 8	0.006 745 8				
H	4.623 957 1	−4.176 369 1	0.007 792 2				
H	5.940 814 1	−1.941 279 5	−0.000 185 2				
H	6.170 139 0	0.572 950 0	−0.007 651 5				
B	3.820 911 3	2.195 506 8	−0.009 640 4				
H	4.862 756 2	2.816 455 2	−0.008 101 2				
H	3.566 701 2	5.003 887 7	0.020 723 6				
H	1.280 026 2	6.065 600 4	0.040 027 1				
H	−1.285 411 8	6.064 503 8	0.038 175 7				

Table TS44: B97-D3/SVP optimized geometry of the V_N

At.	X	Y	Z	At.	X	Y	Z
B	3.572 638 0	3.676 158 9	−0.004 517 7				
H	4.208 456 2	4.697 925 3	0.161 864 8				
N	2.167 546 7	3.605 972 2	0.353 671 4				
B	1.469 284 4	4.758 084 0	0.899 148 6				
H	2.060 474 6	5.782 880 0	1.174 464 2				
N	0.054 578 4	4.657 642 8	1.061 815 1				
H	−0.450 756 7	5.493 967 7	1.343 215 7				
B	−0.708 761 4	3.480 107 4	0.680 167 3				
N	−2.129 274 1	3.563 867 6	0.461 765 4				
H	−2.600 251 0	4.453 760 6	0.601 527 5				
B	−2.842 701 1	2.444 799 4	−0.090 293 4				
N	−4.250 065 9	2.466 200 8	−0.457 555 2				
H	−4.753 848 5	3.349 190 3	−0.432 426 3				
B	−4.999 486 8	1.283 235 4	−0.778 706 3				
H	−6.182 099 4	1.356 961 5	−1.049 940 3				
N	−4.318 873 8	−0.001 353 5	−0.727 825 0				
B	−2.892 588 2	−0.000 882 8	−0.437 544 3				
N	−2.125 162 1	1.194 084 7	−0.227 605 0				
B	−0.721 431 0	0.962 106 0	0.154 248 7				
N	0.014 011 7	2.219 784 9	0.439 286 7				
B	1.416 091 5	2.372 339 7	0.116 272 8				
N	2.073 456 5	1.233 076 7	−0.489 320 4				
B	3.523 747 1	1.249 463 3	−0.704 520 3				
N	4.210 621 1	2.511 418 6	−0.549 988 2				
H	5.208 238 7	2.563 277 3	−0.739 510 4				
N	4.188 608 9	0.001 089 4	−0.988 992 0				
H	5.197 436 6	0.001 404 7	−1.118 283 7				
B	3.524 538 1	−1.247 605 3	−0.704 177 8				
N	2.074 264 7	−1.232 102 6	−0.489 020 5				
B	1.352 317 1	0.000 240 4	−0.591 685 9				
B	−0.720 724 3	−0.962 421 6	0.154 427 7				
N	−2.124 359 3	−1.195 338 3	−0.227 396 0				
B	−2.841 173 4	−2.446 426 2	−0.089 721 0				
N	−4.248 544 9	−2.468 796 8	−0.456 935 5				
B	−4.998 683 7	−1.286 365 9	−0.778 393 9				
H	−6.181 234 2	−1.360 936 8	−1.049 622 7				
H	−4.751 751 8	−3.352 119 1	−0.431 559 8				
N	−2.127 108 0	−3.564 936 8	0.462 672 0				
H	−2.597 575 7	−4.455 081 1	0.602 624 1				
B	−0.706 645 3	−3.480 303 5	0.681 085 8				
N	0.015 423 2	−2.219 657 8	0.439 863 2				
B	1.417 582 3	−2.371 564 6	0.116 884 9				
N	2.169 765 8	−3.604 658 1	0.354 646 8				
B	3.574 877 0	−3.674 078 8	−0.003 496 5				
N	4.212 156 0	−2.509 109 4	−0.549 286 4				
H	5.209 800 8	−2.560 432 8	−0.738 722 2				
H	4.211 288 4	−4.695 405 5	0.163 218 1				
B	1.472 130 9	−4.756 988 3	0.900 426 1				
H	2.063 884 6	−5.781 368 9	1.176 004 6				
N	0.057 376 0	−4.657 287 9	1.063 067 2				
H	−0.447 494 8	−5.493 817 1	1.344 678 1				

Table TS45: B97-D3/SVP optimized geometry of the BN-Layer(N in center)

At.	X	Y	Z	At.	X	Y	Z
N	−4.146 746 0	−3.160 291 4	−0.008 214 7				
H	−5.075 651 3	−3.573 832 4	−0.014 641 1				
B	−4.014 157 0	−1.720 321 5	−0.002 937 2				
N	−5.148 546 4	−0.823 406 7	−0.006 998 4				
H	−6.088 478 2	−1.211 185 0	−0.013 531 6				
B	−5.013 515 6	0.601 677 4	−0.003 384 3				
H	−5.997 772 3	1.311 895 8	−0.006 681 4				
N	−3.675 625 1	1.178 840 0	0.003 865 4				
B	−3.500 581 1	2.618 612 9	0.001 973 0				
H	−4.471 569 8	3.345 158 1	−0.003 856 9				
N	−2.168 753 3	3.192 986 3	0.005 503 2				
B	−1.992 709 2	4.639 387 4	−0.000 063 1				
H	−2.951 803 2	5.383 225 1	−0.003 626 7				
N	−0.663 673 1	5.171 251 3	−0.001 729 7				
H	−0.557 025 1	6.182 448 3	−0.007 046 6				
B	0.517 044 7	4.336 328 6	0.002 411 3				
N	0.344 324 4	2.888 120 4	0.009 821 4				
B	−0.994 580 4	2.317 763 4	0.010 430 1				
N	−1.166 583 9	0.872 628 6	0.013 626 1				
B	−2.504 414 4	0.299 631 3	0.008 699 2				
N	−2.673 568 2	−1.145 777 3	0.006 285 6				
B	−1.510 051 9	−2.020 043 2	0.007 455 4				
N	−1.680 888 4	−3.474 445 4	0.001 236 7				
B	−3.021 493 9	−4.045 212 8	−0.005 951 3				
H	−3.186 129 3	−5.247 726 4	−0.010 272 8				
B	−0.517 469 8	−4.340 596 8	−0.001 938 9				
H	−0.661 132 5	−5.544 745 9	−0.009 221 1				
N	0.816 945 3	−3.772 305 1	0.001 955 5				
B	0.992 775 5	−2.318 494 3	0.008 170 9				
N	−0.172 470 3	−1.446 467 0	0.012 489 8				
B	−0.000 029 7	0.000 014 3	0.014 364 8				
N	1.338 967 1	0.573 842 5	0.014 280 9				
B	1.511 585 1	2.018 899 3	0.011 006 3				
N	2.858 607 5	2.593 455 7	0.006 559 4				
B	3.027 709 7	4.040 674 7	0.000 953 9				
N	1.861 012 6	4.870 172 9	−0.001 167 7				
H	1.995 261 3	5.878 077 8	−0.006 409 8				
H	4.134 894 7	4.537 885 4	−0.002 275 4				
B	4.018 034 5	1.721 995 9	0.003 547 1				
H	5.132 687 5	2.199 652 2	−0.001 947 2				
N	3.849 620 7	0.281 436 9	0.005 565 3				
B	2.504 568 6	−0.297 686 1	0.009 968 9				
N	2.329 189 2	−1.742 354 4	0.007 647 9				
B	3.497 149 1	−2.616 048 7	−0.001 155 8				
N	3.287 457 7	−4.046 776 5	−0.006 605 0				
B	1.985 810 8	−4.642 328 9	−0.004 781 6				
H	1.862 786 2	−5.849 824 7	−0.009 136 8				
H	4.093 223 8	−4.666 984 5	−0.012 969 2				
N	4.810 518 1	−2.010 991 9	−0.004 728 9				
H	5.632 998 2	−2.608 847 9	−0.010 970 8				
B	5.014 308 1	−0.594 145 5	−0.001 239 8				
H	6.137 938 8	−0.135 222 1	−0.004 334 4				

Table TS46: B97-D3/SVP optimized geometry of the BN-Layer(BN in center)

At.	X	Y	Z	At.	X	Y	Z
B	−5.840 290 8	−1.262 072 6	−0.030 331 6				
H	−7.055 012 7	−1.285 187 1	−0.044 866 1				
N	−5.095 246 6	−2.484 396 9	−0.020 294 1				
H	−5.616 369 3	−3.357 318 1	−0.024 852 3				
B	−3.648 024 4	−2.523 938 3	−0.008 675 0				
N	−2.919 305 9	−1.259 108 5	−0.002 280 7				
B	−3.650 737 5	−0.000 006 2	−0.008 305 3				
N	−5.118 231 1	−0.000 015 6	−0.023 086 2				
B	−5.840 295 8	1.262 051 3	−0.030 276 0				
H	−7.055 022 4	1.285 150 4	−0.044 695 9				
N	−5.095 252 3	2.484 350 1	−0.020 309 6				
H	−5.616 391 6	3.357 300 8	−0.024 833 6				
B	−3.648 038 7	2.523 934 7	−0.008 805 4				
N	−2.900 498 9	3.757 410 7	−0.005 545 1				
H	−3.408 262 0	4.638 363 2	−0.010 801 8				
B	−1.459 708 0	3.786 057 4	−0.000 957 7				
N	−0.698 652 1	5.018 493 3	−0.002 812 8				
H	−1.192 600 7	5.907 095 0	−0.008 108 1				
B	0.731 333 9	5.050 809 4	0.000 344 1				
H	1.321 966 2	6.111 750 4	−0.004 068 1				
N	1.461 026 2	3.789 775 3	0.007 935 8				
B	0.723 551 6	2.522 999 1	0.009 139 5				
N	−0.730 287 3	2.521 323 6	0.004 667 1				
B	−1.462 375 0	1.260 771 4	0.004 348 1				
N	−2.919 308 3	1.259 093 2	−0.002 354 6				
N	−0.732 693 2	0.000 002 8	0.008 682 5				
B	−1.462 369 4	−1.260 768 4	0.004 472 1				
N	−0.730 283 7	−2.521 324 9	0.004 972 6				
B	−1.459 686 9	−3.786 051 7	−0.000 536 3				
N	−2.900 500 9	−3.757 445 2	−0.005 251 2				
H	−3.408 243 2	−4.638 361 9	−0.010 425 9				
N	−0.698 648 6	−5.018 508 1	−0.002 139 1				
H	−1.192 565 8	−5.907 089 1	−0.007 357 5				
B	0.731 361 4	−5.050 801 0	0.001 225 9				
N	1.461 041 4	−3.789 769 4	0.008 692 1				
B	0.723 561 2	−2.522 992 9	0.009 568 0				
N	1.451 998 2	−1.262 041 8	0.014 444 5				
B	0.722 532 5	0.000 002 9	0.012 890 0				
N	1.451 994 6	1.262 051 1	0.014 206 9				
B	2.906 720 4	1.262 019 2	0.015 687 0				
N	3.638 378 7	2.531 260 0	0.015 881 5				
B	2.910 423 4	3.785 714 8	0.013 586 2				
H	3.517 481 1	4.835 927 7	0.016 112 3				
B	5.094 831 3	2.527 828 2	0.013 761 5				
H	5.720 170 8	3.568 506 7	0.013 375 0				
N	5.780 216 4	1.271 002 4	0.010 968 0				
B	5.090 155 0	0.000 008 6	0.012 709 8				
N	3.632 103 9	0.000 006 3	0.015 932 6				
B	2.906 724 4	−1.262 011 0	0.016 002 5				
N	3.638 388 3	−2.531 245 3	0.016 589 6				
B	2.910 441 4	−3.785 703 1	0.014 540 7				
H	3.517 507 7	−4.835 911 1	0.017 450 8				
B	5.094 839 6	−2.527 805 3	0.014 590 0				
N	5.780 216 2	−1.270 979 3	0.011 444 3				
H	6.796 887 6	−1.284 012 1	0.008 315 6				
H	5.720 180 5	−3.568 481 2	0.014 585 7				
H	6.796 879 8	1.284 027 6	0.007 759 2				
H	1.321 989 8	−6.111 741 8	−0.002 911 4				

Table TS47: B97-D3/SVP optimized geometry of the BN-Layer(N in center)

At.	X	Y	Z	At.	X	Y	Z
B	3.685 479 4	3.754 687 1	0.001 404 2				
N	3.732 926 9	2.301 647 9	−0.000 323 4				
B	2.483 078 5	1.531 069 2	−0.002 473 4				
N	1.200 127 8	2.219 305 4	−0.002 995 6				
B	1.157 166 7	3.678 059 3	−0.000 141 7				
N	2.410 197 9	4.404 326 8	0.001 240 4				
H	2.395 357 2	5.420 822 2	0.002 460 0				
N	−0.125 470 4	4.336 443 0	0.001 599 4				
H	−0.154 915 4	5.352 780 6	0.005 055 2				
B	−1.367 907 7	3.605 023 2	−0.000 742 1				
N	−2.660 822 3	4.257 665 5	−0.000 065 7				
H	−2.704 759 3	5.273 350 2	0.000 909 6				
B	−3.896 456 7	3.535 460 6	−0.000 260 5				
H	−4.955 086 6	4.132 294 1	0.000 882 2				
N	−3.859 800 7	2.082 078 9	−0.001 571 3				
B	−2.567 485 4	1.385 016 9	−0.003 189 0				
N	−1.326 443 3	2.146 231 6	−0.003 467 1				
B	−0.042 132 1	1.455 912 4	−0.004 023 5				
N	0.000 021 9	0.000 020 5	−0.004 903 2				
B	1.281 850 0	−0.691 442 0	−0.004 026 7				
N	2.521 867 3	0.075 607 0	−0.003 190 3				
B	3.805 922 6	−0.617 890 3	−0.000 641 6				
N	5.017 607 1	0.175 505 4	0.000 410 6				
B	5.009 876 2	1.606 724 3	0.000 842 3				
H	6.056 017 4	2.225 133 1	0.002 341 4				
H	5.919 105 7	−0.294 322 7	0.001 267 0				
N	3.818 213 6	−2.059 638 7	0.001 175 6				
H	4.713 038 1	−2.542 409 9	0.004 429 4				
B	2.606 670 1	−2.841 226 3	−0.000 621 9				
N	1.321 914 7	−2.149 049 5	−0.003 255 1				
B	0.084 406 6	−2.916 030 5	−0.002 527 3				
N	−1.195 370 6	−2.221 823 8	−0.003 108 8				
B	−1.239 706 2	−0.764 338 1	−0.004 097 9				
N	−2.521 988 8	−0.070 245 9	−0.003 543 6				
B	−3.763 805 8	−0.836 813 1	−0.000 785 7				
N	−5.019 324 0	−0.114 794 5	−0.000 216 2				
B	−5.094 435 7	1.314 473 3	−0.000 385 7				
H	−6.174 583 0	1.871 342 5	0.000 667 3				
H	−5.892 212 4	−0.635 927 7	0.000 674 4				
N	−3.692 676 3	−2.276 839 1	0.001 712 9				
H	−4.558 173 7	−2.810 456 4	0.005 383 0				
B	−2.438 003 4	−2.987 081 9	−0.000 118 6				
N	−2.356 723 2	−4.433 146 7	0.001 374 9				
B	−1.113 421 7	−5.142 104 2	0.001 698 7				
N	0.126 875 3	−4.383 742 9	−0.000 218 4				
B	1.408 878 7	−5.069 227 4	0.001 024 4				
N	2.609 177 8	−4.289 619 2	0.000 544 1				
H	3.496 947 5	−4.785 007 5	0.001 486 6				
H	1.466 636 7	−6.283 142 8	0.002 642 3				
H	−1.101 125 6	−6.357 341 8	0.003 810 3				
H	−3.214 395 3	−4.978 976 5	0.002 685 4				
H	4.707 863 9	4.411 658 0	0.003 172 7				

References

(1) Trofimov, A. B.; Schirmer, J. *J. Phys. B* **1995**, *28*, 2299–2324.

(2) Stanton, J. F.; Bartlett, R. J. *J. Chem. Phys.* **1993**, *98*, 7029–7039.

(3) Dreuw, A.; Head-Gordon, M. *J. Am. Chem. Soc.* **2004**, *126*, 4007–4016.
