# Supplementary figures and images for: Influence of the second layer on geometry and spectral properties of doped two-dimensional hexagonal boron nitride
Source: J Mol Model. 2020 Jul 27;26(8):216. doi: 10.1007/s00894-020-04456-8 (PMC7384999; doi:10.1007/s00894-020-04456-8)

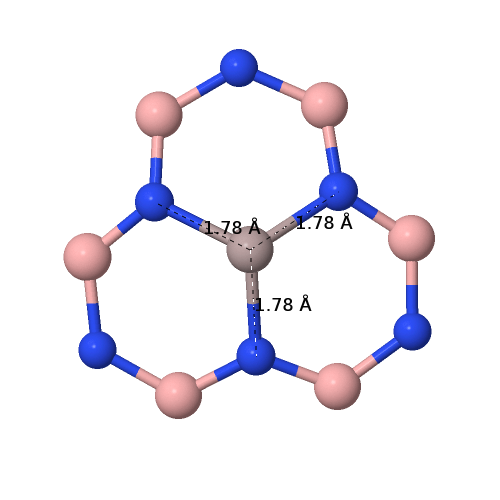

Supplement: Supplementary file 2 — (ZIP 3.16 MB) [file 894_2020_4456_MOESM2_ESM.zip › albb_52_52_-z.png]

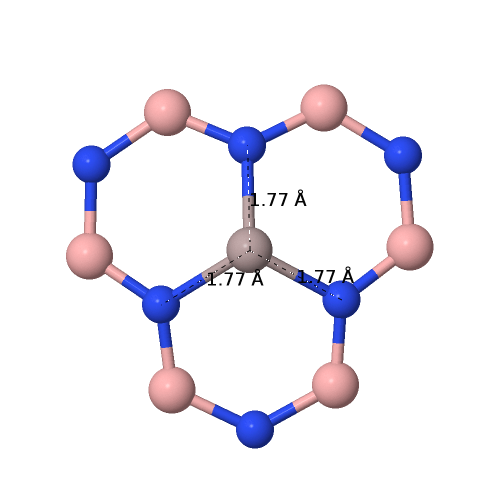

Supplement: Supplementary file 2 — (ZIP 3.16 MB) [file 894_2020_4456_MOESM2_ESM.zip › albp_52_52_-z.png]

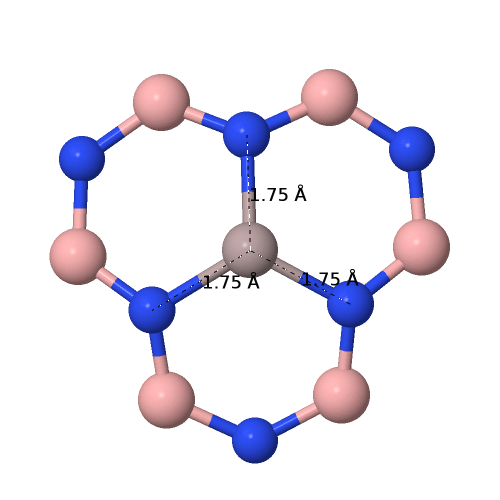

Supplement: Supplementary file 2 — (ZIP 3.16 MB) [file 894_2020_4456_MOESM2_ESM.zip › alb.png]

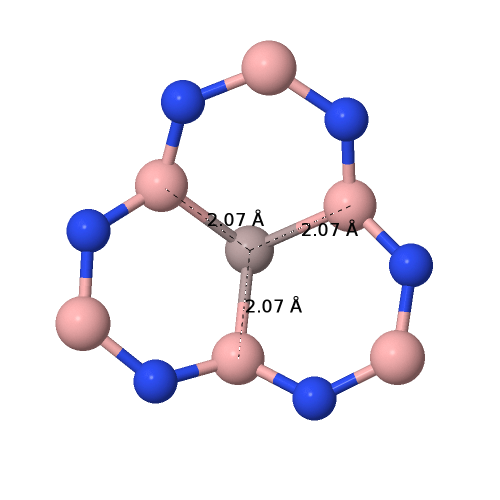

Supplement: Supplementary file 2 — (ZIP 3.16 MB) [file 894_2020_4456_MOESM2_ESM.zip › alnb_52_52_-z.png]

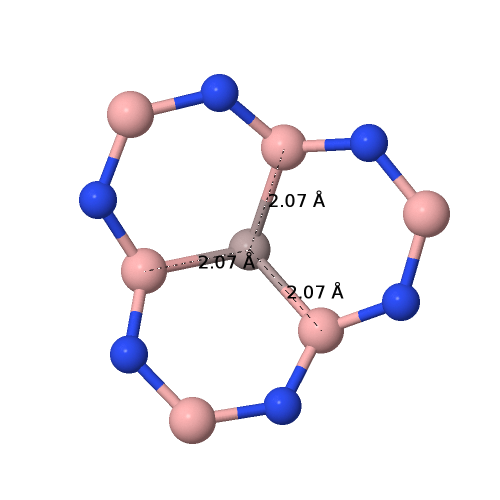

Supplement: Supplementary file 2 — (ZIP 3.16 MB) [file 894_2020_4456_MOESM2_ESM.zip › alnp_52_52_-z.png]

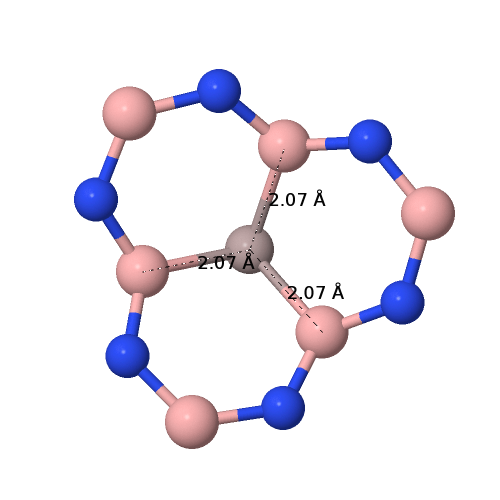

Supplement: Supplementary file 2 — (ZIP 3.16 MB) [file 894_2020_4456_MOESM2_ESM.zip › aln.png]

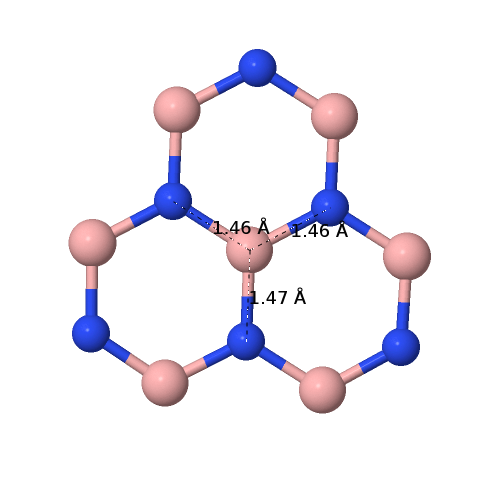

Supplement: Supplementary file 2 — (ZIP 3.16 MB) [file 894_2020_4456_MOESM2_ESM.zip › b_52_52_-z.png]

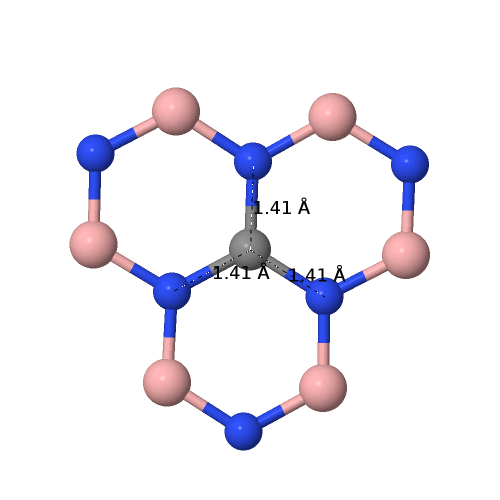

Supplement: Supplementary file 2 — (ZIP 3.16 MB) [file 894_2020_4456_MOESM2_ESM.zip › cbb_52_52_-z.png]

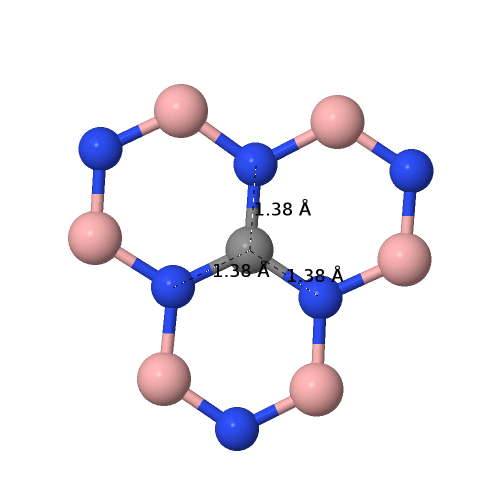

Supplement: Supplementary file 2 — (ZIP 3.16 MB) [file 894_2020_4456_MOESM2_ESM.zip › cbcnb_52_52_-a-z.png]

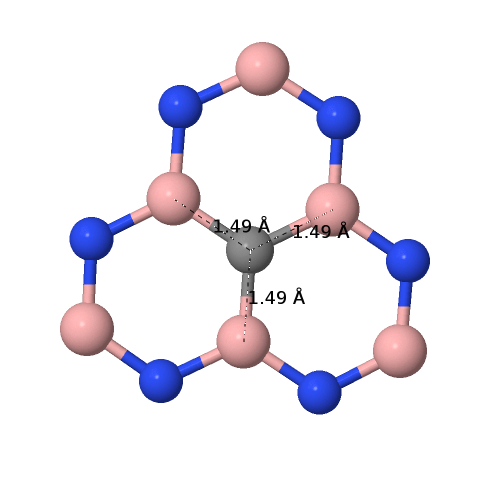

Supplement: Supplementary file 2 — (ZIP 3.16 MB) [file 894_2020_4456_MOESM2_ESM.zip › cbcnb_52_52_-b-z.png]

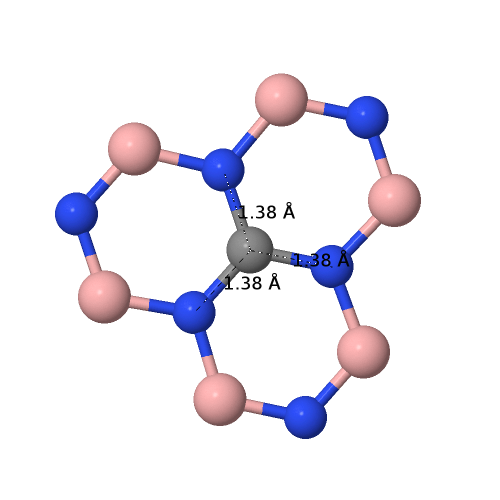

Supplement: Supplementary file 2 — (ZIP 3.16 MB) [file 894_2020_4456_MOESM2_ESM.zip › cbcnp_52_52_-a.png]

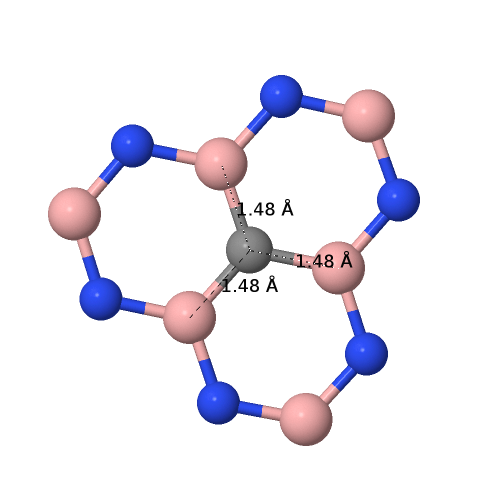

Supplement: Supplementary file 2 — (ZIP 3.16 MB) [file 894_2020_4456_MOESM2_ESM.zip › cbcnp_52_52_-b.png]

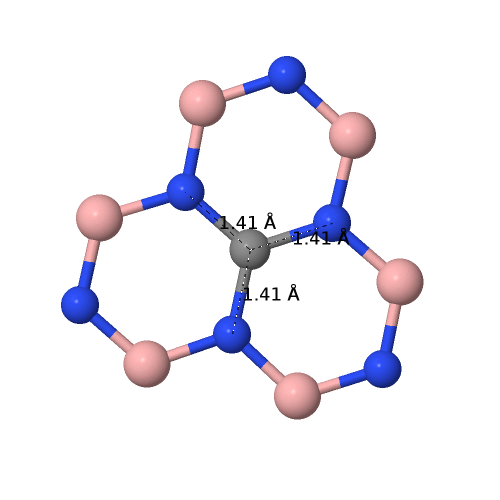

Supplement: Supplementary file 2 — (ZIP 3.16 MB) [file 894_2020_4456_MOESM2_ESM.zip › cbp_52_52_-z.png]

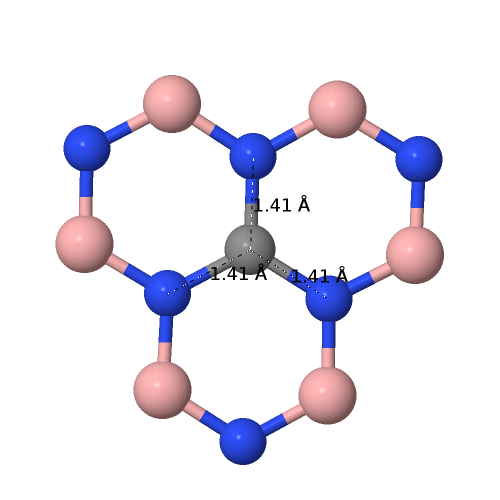

Supplement: Supplementary file 2 — (ZIP 3.16 MB) [file 894_2020_4456_MOESM2_ESM.zip › cb.png]

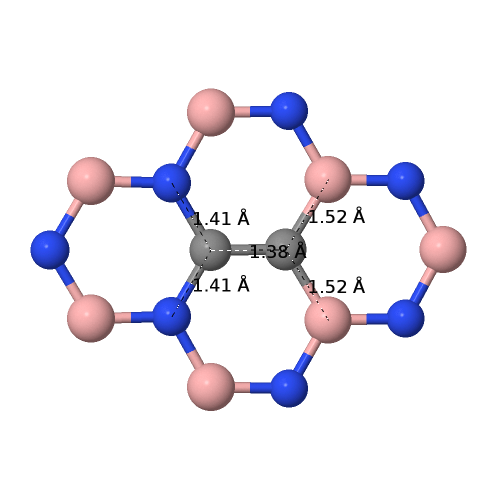

Supplement: Supplementary file 2 — (ZIP 3.16 MB) [file 894_2020_4456_MOESM2_ESM.zip › ccbnb_58_58_-z.png]

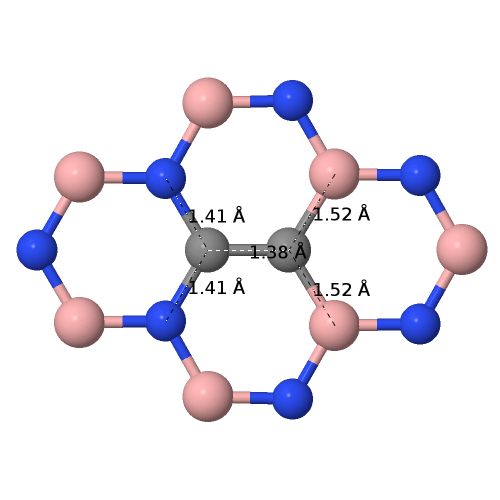

Supplement: Supplementary file 2 — (ZIP 3.16 MB) [file 894_2020_4456_MOESM2_ESM.zip › ccbnp_58_58_-z.png]

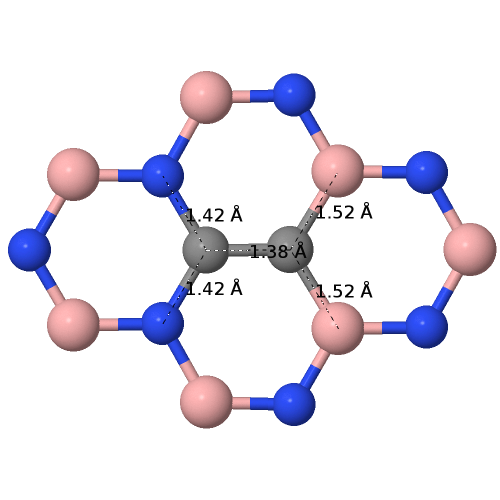

Supplement: Supplementary file 2 — (ZIP 3.16 MB) [file 894_2020_4456_MOESM2_ESM.zip › ccbn.png]

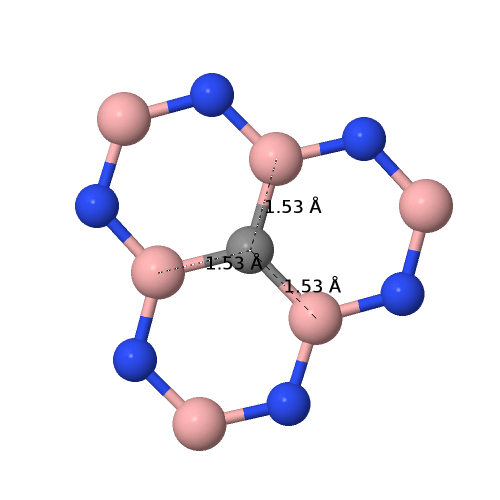

Supplement: Supplementary file 2 — (ZIP 3.16 MB) [file 894_2020_4456_MOESM2_ESM.zip › cnb_52_52_-z.png]

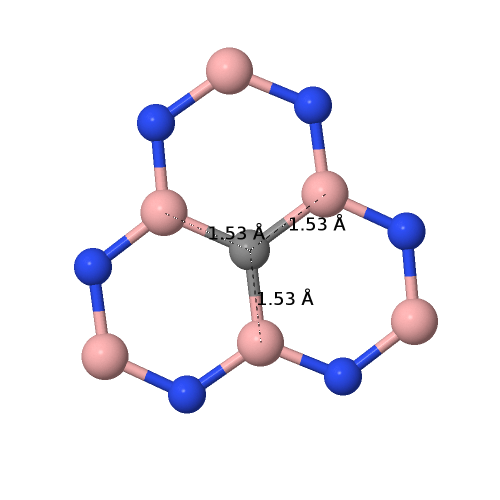

Supplement: Supplementary file 2 — (ZIP 3.16 MB) [file 894_2020_4456_MOESM2_ESM.zip › cnp_52_52_-z.png]

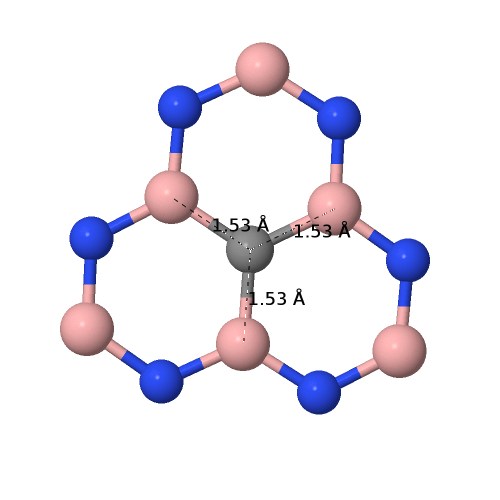

Supplement: Supplementary file 2 — (ZIP 3.16 MB) [file 894_2020_4456_MOESM2_ESM.zip › cn.png]

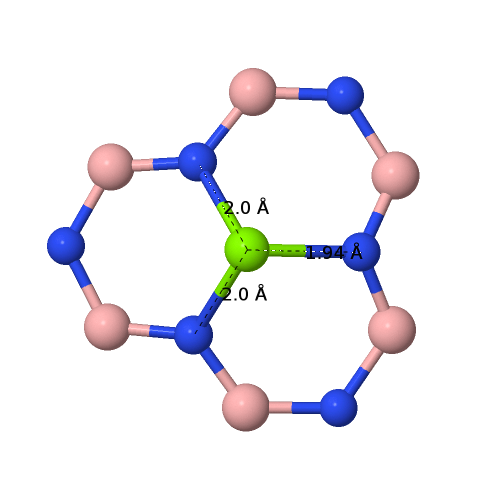

Supplement: Supplementary file 2 — (ZIP 3.16 MB) [file 894_2020_4456_MOESM2_ESM.zip › mgbb_52_52_-z.png]

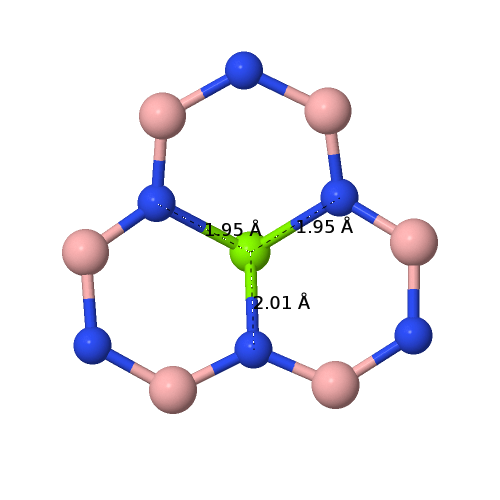

Supplement: Supplementary file 2 — (ZIP 3.16 MB) [file 894_2020_4456_MOESM2_ESM.zip › mgbp_52_52_-z.png]

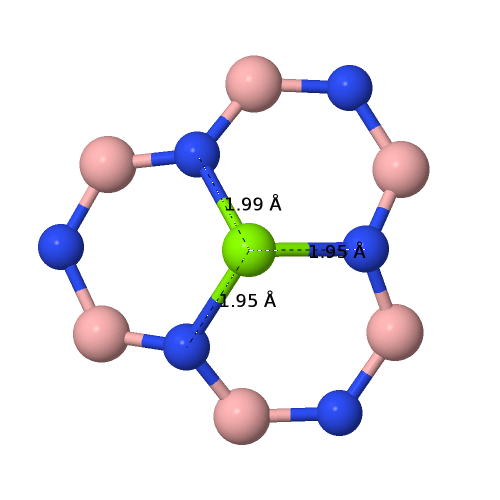

Supplement: Supplementary file 2 — (ZIP 3.16 MB) [file 894_2020_4456_MOESM2_ESM.zip › mgb.png]

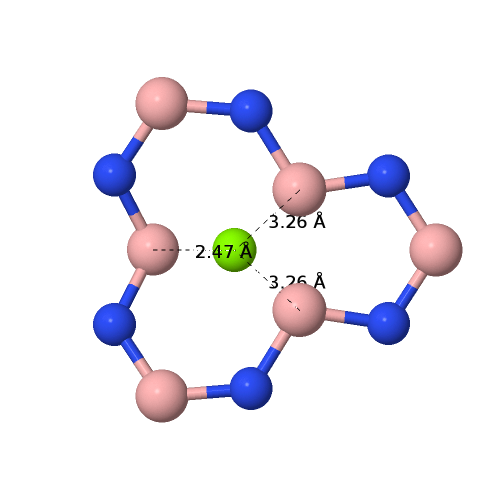

Supplement: Supplementary file 2 — (ZIP 3.16 MB) [file 894_2020_4456_MOESM2_ESM.zip › mgnb_52_52_-z.png]

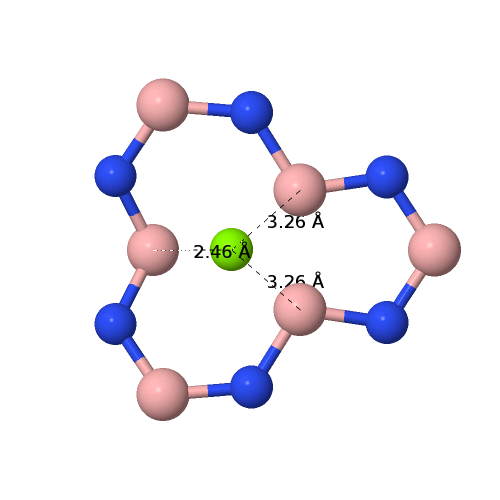

Supplement: Supplementary file 2 — (ZIP 3.16 MB) [file 894_2020_4456_MOESM2_ESM.zip › mgnp_52_52_-z.png]

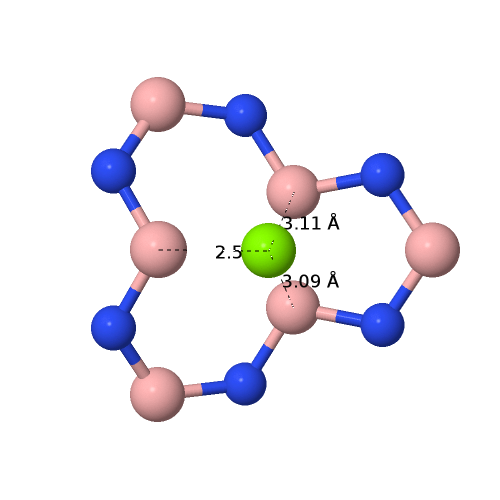

Supplement: Supplementary file 2 — (ZIP 3.16 MB) [file 894_2020_4456_MOESM2_ESM.zip › mgn.png]

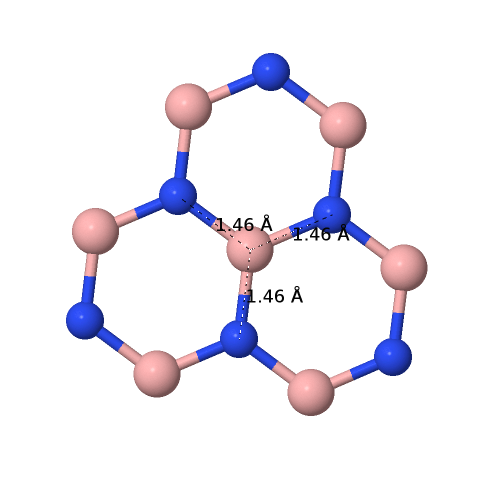

Supplement: Supplementary file 2 — (ZIP 3.16 MB) [file 894_2020_4456_MOESM2_ESM.zip › p_52_52_-z.png]

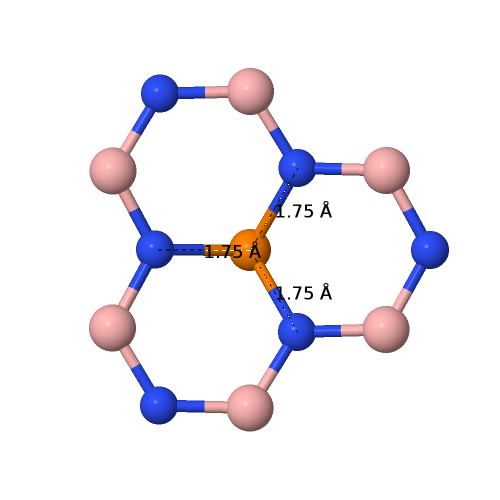

Supplement: Supplementary file 2 — (ZIP 3.16 MB) [file 894_2020_4456_MOESM2_ESM.zip › pbb_52_52_-z.png]

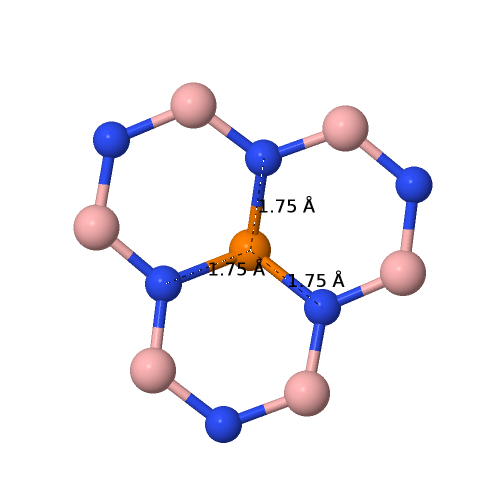

Supplement: Supplementary file 2 — (ZIP 3.16 MB) [file 894_2020_4456_MOESM2_ESM.zip › pbp_52_52_-z.png]

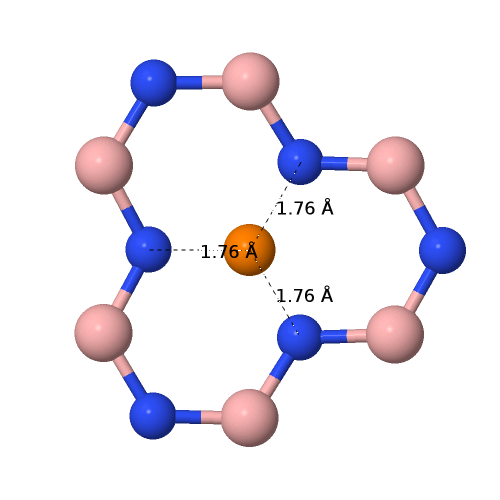

Supplement: Supplementary file 2 — (ZIP 3.16 MB) [file 894_2020_4456_MOESM2_ESM.zip › pb.png]

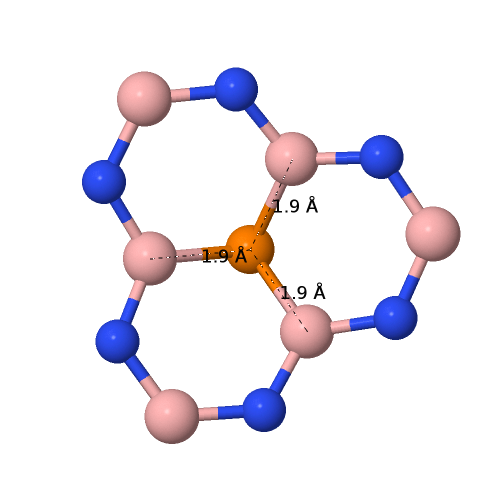

Supplement: Supplementary file 2 — (ZIP 3.16 MB) [file 894_2020_4456_MOESM2_ESM.zip › pnb_52_52_-z.png]

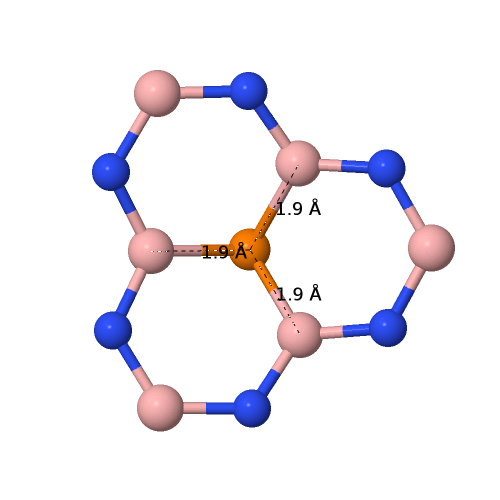

Supplement: Supplementary file 2 — (ZIP 3.16 MB) [file 894_2020_4456_MOESM2_ESM.zip › pnp_52_52_-z.png]

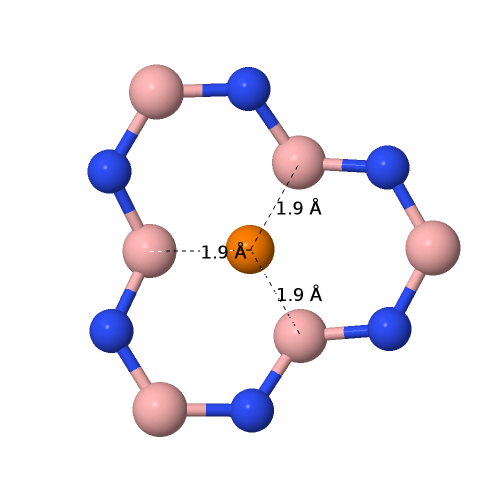

Supplement: Supplementary file 2 — (ZIP 3.16 MB) [file 894_2020_4456_MOESM2_ESM.zip › pn.png]

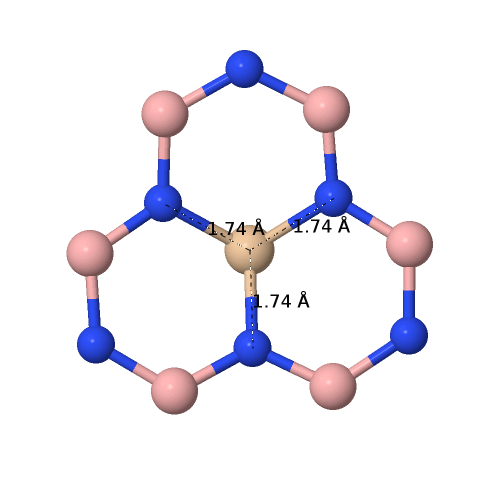

Supplement: Supplementary file 2 — (ZIP 3.16 MB) [file 894_2020_4456_MOESM2_ESM.zip › sibb_52_52_-z.png]

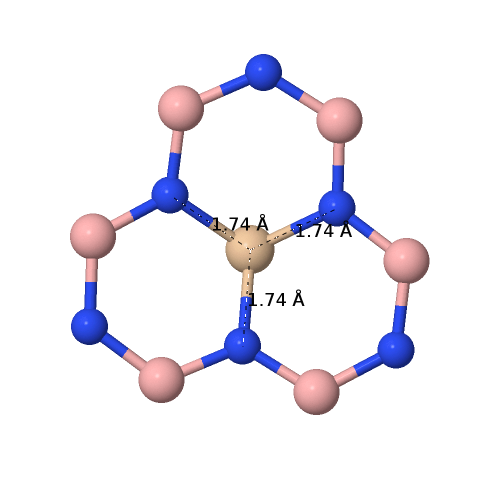

Supplement: Supplementary file 2 — (ZIP 3.16 MB) [file 894_2020_4456_MOESM2_ESM.zip › sibp_52_52_-z.png]

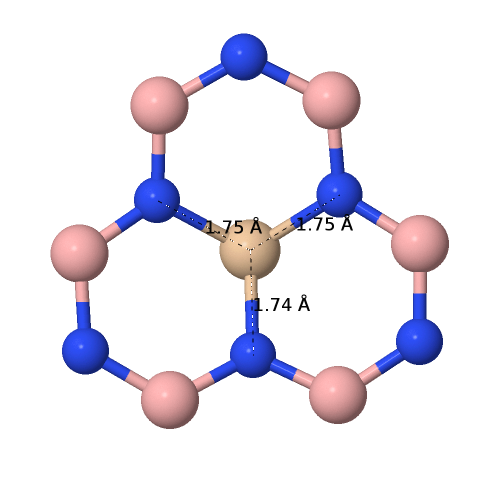

Supplement: Supplementary file 2 — (ZIP 3.16 MB) [file 894_2020_4456_MOESM2_ESM.zip › sib.png]

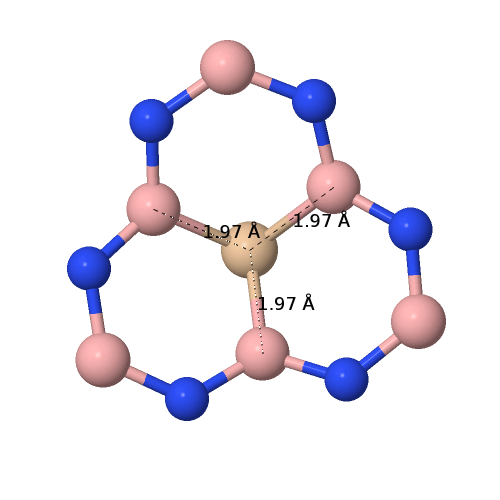

Supplement: Supplementary file 2 — (ZIP 3.16 MB) [file 894_2020_4456_MOESM2_ESM.zip › sinb_52_52_-z.png]

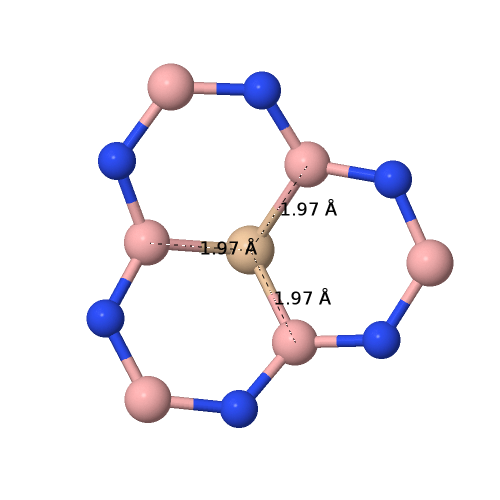

Supplement: Supplementary file 2 — (ZIP 3.16 MB) [file 894_2020_4456_MOESM2_ESM.zip › sinp_52_52_-z.png]

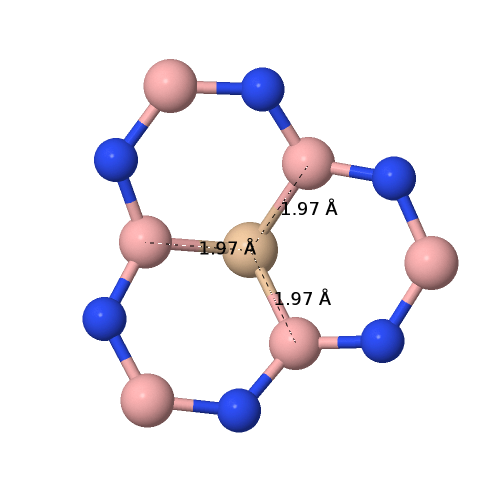

Supplement: Supplementary file 2 — (ZIP 3.16 MB) [file 894_2020_4456_MOESM2_ESM.zip › sin.png]

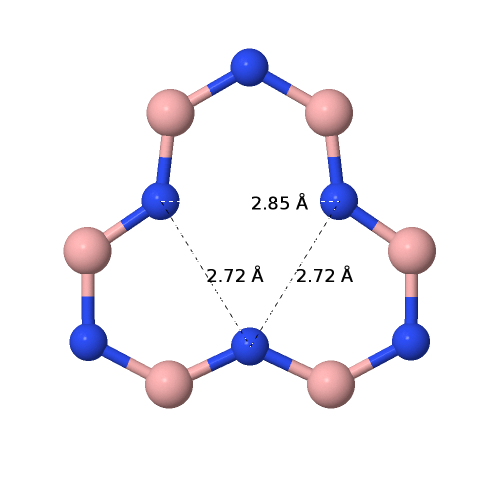

Supplement: Supplementary file 2 — (ZIP 3.16 MB) [file 894_2020_4456_MOESM2_ESM.zip › vbb.png]

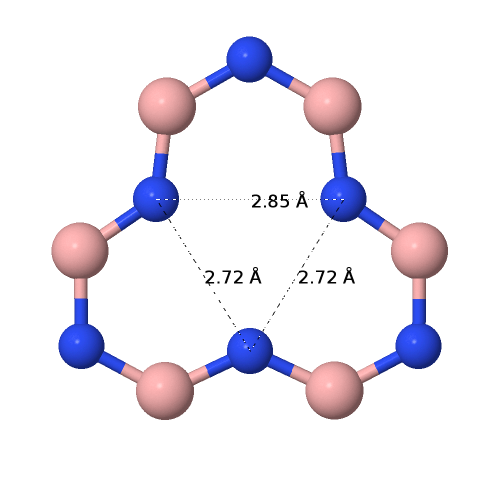

Supplement: Supplementary file 2 — (ZIP 3.16 MB) [file 894_2020_4456_MOESM2_ESM.zip › vb.png]

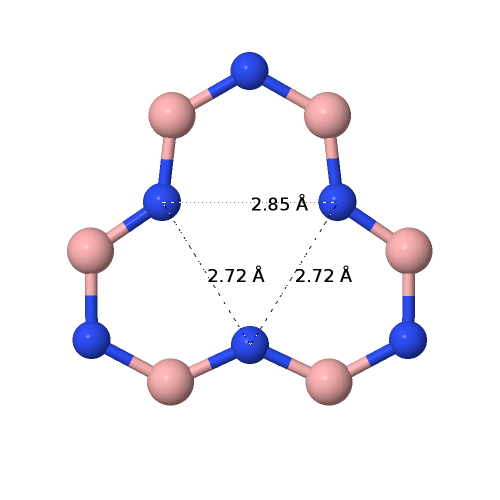

Supplement: Supplementary file 2 — (ZIP 3.16 MB) [file 894_2020_4456_MOESM2_ESM.zip › vbp.png]

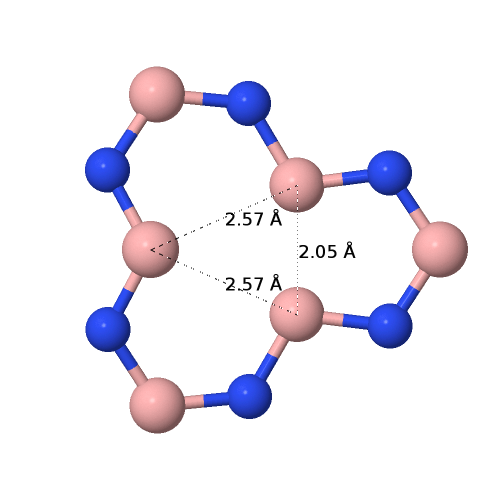

Supplement: Supplementary file 2 — (ZIP 3.16 MB) [file 894_2020_4456_MOESM2_ESM.zip › vnb.png]

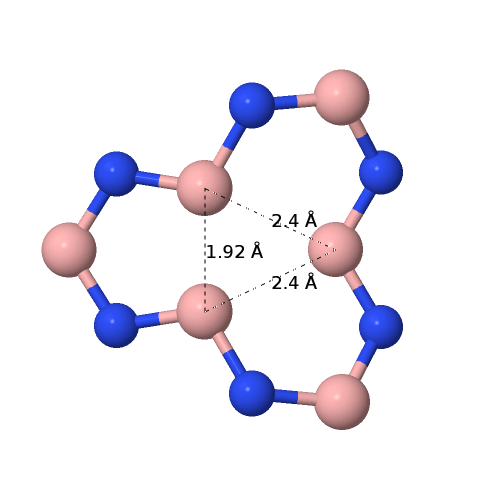

Supplement: Supplementary file 2 — (ZIP 3.16 MB) [file 894_2020_4456_MOESM2_ESM.zip › vn.png]

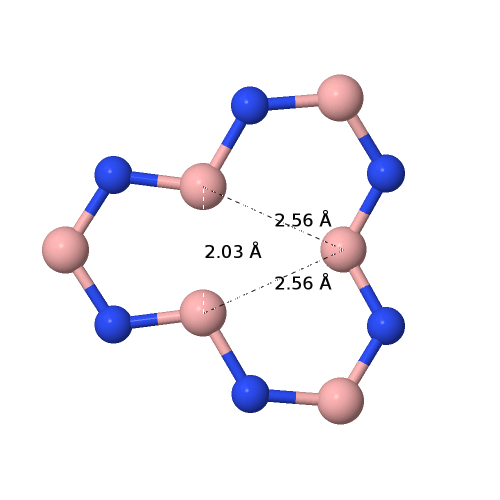

Supplement: Supplementary file 2 — (ZIP 3.16 MB) [file 894_2020_4456_MOESM2_ESM.zip › vnp.png]
